# *Thymus hirtus* Willd. ssp. *algeriensis* Boiss. and Reut: A Comprehensive Review on Phytochemistry, Bioactivities, and Health-Enhancing Effects

**DOI:** 10.3390/foods11203195

**Published:** 2022-10-13

**Authors:** Radhia Aitfella Lahlou, Nsevolo Samba, Pedro Soeiro, Gilberto Alves, Ana Carolina Gonçalves, Luís R. Silva, Samuel Silvestre, Jesus Rodilla, Maria Isabel Ismael

**Affiliations:** 1 Chemistry Department, University of Beira Interior, 6201-001 Covilhã, Portugal; 2 Fiber Materials and Environmental Technologies (FibEnTech), University of Beira Interior, 6201-001 Covilhã, Portugal; 3 Biology Department, Faculty of Sciences, University of M’Hamed Bougara, Boumerdes 35000, Algeria; 4 CICS-UBI—Health Sciences Research Center, University of Beira Interior, Av. Infante D. Henrique, 6200-506 Covilhã, Portugal; 5 CPIRN-UDI/IPG—Centro de Potencial e Inovação em Recursos Naturais, Unidade de Investigação Para o Desenvolvimento do Interior do Instituto Politécnico da Guarda, 6300-559 Guarda, Portugal; 6 CNC—Center for Neuroscience and Cell Biology, University of Coimbra, Rua Larga, 3004-517 Coimbra, Portugal

**Keywords:** *Thymus algeriensis*, distribution, botanical aspects, folk medicine, phytochemistry, pharmacological reports, bioactivities, health promotion

## Abstract

Members of the Lamiaceae family are considered chief sources of bioactive therapeutic agents. They are important ornamental, medicinal, and aromatic plants, many of which are used in traditional and modern medicine and in the food, cosmetic, and pharmaceutical industries. In North Africa, on the Mediterranean side, there is the following particularly interesting Lamiaceous species: *Thymus hirtus* Willd. sp. *Algeriensis* Boiss. Et Reut. The populations of this endemic plant are distributed from the subhumid to the lower arid zone and are mainly employed as ethnomedicinal remedies in the following Maghreb countries: Algeria, Libya, Morocco, and Tunisia. In fact, they have been applied as antimicrobial agents, antispasmodics, astringents, expectorants, and preservatives for several food products. The species is commonly consumed as a tea or infusion and is used against hypercholesterolemia, diabetes, respiratory ailments, heart disease, and food poisoning. These medicinal uses are related to constituents with many biological characteristics, including antimicrobial, antioxidant, anticancer, anti-ulcer, anti-diabetic, insecticidal, and anti-inflammatory activities. This review aims to present an overview of the botanical characteristics and geographical distribution of *Thymus algeriensis* Boiss. Et Reut and its traditional uses. This manuscript also examines the phytochemical profile and its correlation with biological activities revealed by in vitro and in vivo studies.

## 1. Introduction

Lamiaceae (= Labiatae Adans., the mint family) is one of the groups of plants most widely distributed worldwide, with about 6900–7200 species [1,2]. It is a large family composed of annual, biennial, and perennial herbs, subshrubs, shrubs or trees, large forest forms, and even woody climbers [3]. It includes 242 genera with 200 or even more species, such as Salvia (986), Scutellaria (468), Stachys (375), and Plectranthus L’Hér. (325), Thymus (315), Hyptis Jacq. (295), Teucrium (287), Nepeta (251), and Vitex (223) [4], which are distributed in warm and temperate regions worldwide [5]. In addition, this family has great diversity with a cosmopolitan distribution [6]. They can adapt to different ecosystems and be cultivated easily, but without inhabiting the coldest high-altitude or high-latitude regions [7]. Worldwide, there are the following seven regions of high Lamiaceae diversity: (1) the Mediterranean and southwestern Central Asia; (2) Africa south of the Sahel and Madagascar; (3) China; (4) Australia; (5) South America; (6) North America and Mexico; (7) Indo-Malesia region (Southeast Asia) [3,8].

Lamiaceae is considered one of the most advanced plant families in floral structures [9]. They are plants with a quadrangular, usually fragrant stem, and their leaves are usually opposite without stipules [10]. Lamiaceae are plants that are usually fragrant and aromatic, especially *Nepetoideae* [3]. They are used in traditional and modern medicine, food, flavoring, perfumery, pesticide, and pharmaceutical industries [11,12,13,14,15]. Indeed, more than 150 plants containing essential oils, oleoresins (solvent-free), and natural extracts (including distillates) have been recognized by the US Food and Drug Administration as safe for human consumption, with no consumption limitations [16]. In this context, the Lamiaceae family deserves special mention as it is endowed with antioxidant [17], anti-inflammatory [18], hepatoprotective [19], antitumor [20], anti-mutagenic [21], radioprotective [22], antibacterial [23], antifungal [24], antiviral [25], and insecticidal properties [14], mainly due to its high content of phenolic compounds. Extracts of these plants, and their essential oils, are rich in a wide variety of secondary metabolites that have also shown potential properties for the treatment of depression [26,27] and pain [28], as well as diabetes [29,30] and cardiovascular disorders [11].

In Northwest Africa, more precisely in the greater Maghreb, represented by Algeria, Libya, Morocco, Mauritania, and Tunisia, the Lamiaceae are composed of a multitude of species distributed almost throughout the region [31,32]. The Maghreb is situated at the floristic crossroads between the Atlantic Ocean and the Mediterranean Sea and is separated from the Middle East by a broad band of Libyan and Egyptian deserts. Botanical influences from different directions could explain the Maghreban floristic biodiversity. Several regions of the Mediterranean climate are found, from desert to mountain, with transitions from plains to steppes [33,34]. The Maghreb has about 8000 km of coastline, bathed by the Atlantic Ocean to the west and the Mediterranean Sea to the north. The temperature and humidity differences between these two currents are the reason for vegetation and climate contrasts between the west and the north of the Maghreb. Boreal Europe lies to the north, Africa to the south, and Asia to the southeast. This particular geographical situation makes northern Africa one of the main routes for the dispersion of flora and fauna.

The Maghreb has a large and diversified heritage of aromatic and medicinal plants widely used in traditional medicine by local populations. This richness is also reflected in a wide culture of phytopharmacology, particularly among herbalists and folk healers. The Maghreb countries have a shared cultural heritage. Traditional, religious, and socio-economic associations mean that the way of life is comparable in all four territories. This similarity is reflected in the traditional uses of plants belonging to the Lamiaceae family. Indeed, its species are the most frequently cited in ethnopharmacological surveys, particularly in Algeria, Morocco, and Tunisia [33,34,35,36,37]. The Lamiaceae are represented by 29 genera in Algeria, 11 in Mauritania, 32 in Morocco, 22 in Libya, and in Tunisia by 26 genera [31,32]. In this review, we consider specifically the genus *Thymus* L., widespread in many territories in the Mediterranean regions, with much endemism [38,39,40,41,42] as those that grow in Northwest Africa and distributed on the whole coastline until the arid zones [31,43]. It is represented by 17 species and subspecies in Algeria, of which three are endemic [32,44]. These species are present from the north of Algiers to the Saharan Atlas and from the Constantine region to Oran [45]. Twenty-nine Moroccan species and subspecies, of which sixteen are endemic, are listed and found mainly in the plains or mountains, in rocky areas, scrublands, lawns, or scrub [32,46]. Only three Tunisian *Thymus* species grow at altitudes ranging from 120 to 1100 m in a subhumid or lower arid bioclimate [32,46,47]. Finally, in Mauritania, the *Thymus* genera do not exist [32,46].

According to taxonomic studies [31,43,46], twelve species and subspecies are distributed between Algeria and Morocco. In contrast, there are only three common species between Tunisia and Algeria and only one between the four countries, namely, *Thymus algeriensis* Boiss. and Reut. Many studies have reported that the by-products and compounds isolated from this species exhibit various biological activities, such as antimicrobial, anti-inflammatory, antioxidant, and cytotoxic [48,49,50,51,52,53,54,55,56,57,58,59,60,61,62,63]. In short, this review will help provide information on the traditional uses, phytochemistry, and pharmacology of *T. algeriensis*. These data will be valuable to discover gaps and explore potentials that require further research on the chemistry and health benefits of this species, and to generate more interest in the species *T. algeriensis* in the future.

## 2. Methodology

The updated literature was obtained from the following databases: PubMed, Web of Science, Elsevier, ACS Publications, Springer, NCBI, Wiley Online Library, google scholar, and other published documents (books and Ph.D. and MSc theses). The keywords used to search the literature sources include “Thymus”, “*Thymus algeriensis*”, and “*Thymus hirtus algeriensis*” in combination with Maghreb, Algeria, Libya, Morocco, Tunisia, “botanical description”, “phytochemical compounds”, “traditional uses”, and “pharmacological activities”. We collected all published works from the library from January 1900 to 30 June 2022. Only published data were included in this study, and all reports in all languages were included without exception.

Occurrence data were downloaded from the Global Biodiversity Information Facility (www.GBIF.org (accessed on 8 June 2022) GBIF Occurrence Download (https://doi.org/10.15468/dl.75vs2f (accessed on 8 June 2022), and distribution maps were taken from the site (https://africanplantdatabase.ch/ (accessed on 21 June 2022), whose data were confirmed with that of the Global Biodiversity Information Facility and that of the Maghreb eflora project website (https://efloramaghreb.org/ (accessed on 21 June 2022).

Species data were collected from Euro+Med PlantBase and the website (https://europlusmed.org/ (accessed on 17 June 2022). Scientific names and synonyms were validated by the Kew Plants of the World database (www.plantsoftheworldonline.org (accessed on 16 June 2022).

We used chemical structures of PubChem (https://pubchem.ncbi.nlm.nih.gov/ (accessed 10 July 2022) and SciFinder (www.scifinder.cas.org (accessed 15 July 2022).

## 3. *Thymus* Genera: An Overview

The word Thymus comes from the Greek “*thyo*”, which means “*offering*” (to be burnt) and “*perfume*” because of the pleasant smell that the plant gives off naturally when burnt [64,65]. In ancient times, the Sumerians and Egyptians used it for embalming their dead (the mummification process). The Romans burned thyme to purify the air and keep pests away [66,67]. The name *“Thyme”* comes from the Greek word “*Thymos*” [68], meaning smell. In the Azores, Madeira and the western part of the Iberian Peninsula, Thyme has the Portuguese names “*tomentelo*” or “*tormentelo*”, “tomelo do pais”, “*tomentelo do pais*” or “*tomilho*” [65]. In the mountains of Ethiopia, it is known as *“rausi”*. Moreover, the African northwest has the following several Arabic vernacular names: *“Djertil”, “Hamzoucha”,* “*Mezouqach*”, and “*Khieta*”, and Berber names such as “*Azoukni*”, “*Tazuknite*”, “*Rebba*”, “*Djouchchen*”, and “*Touchna*” [69]. In Morocco, it is also called “*azukenni*” [65].

The genus *Thymus,* described by Carl Linnaeus in *Species Plantarum,* belongs to the monophyletic group of the subfamily *Nepetoideae* Kostel, the tribe *Mentheae* Dumort and the subtribe *Menthinae* Endl [3,70]. The species can be sub-shrubs or shrubs, often herbaceous above, usually gynodioic and aromatic. They grow spontaneously on dry, rocky slopes and in scrubland. The stems of species are generally ± quadrangular, hairy all round, on two opposite sides, or only on the corners. The leaves are small, entire, and frequently revolute and form compact, highly branched clumps that rise to about 20 cm above the ground [45]. They have inflorescences of whorls forming a terminal, condensed, often spiciform or interrupted thyrse [46]. These characteristics can have a high degree of polymorphism, demonstrating the complexity of the genus *Thymus* from a taxonomic and systematic point of view. Indeed, this hybridization has been observed between species belonging to different sections and between species with varying ploidy levels, resulting in different chemical compositions [65,71]. Eight sections can be distinguished in the genus from a taxonomic and geographical point of view, as follows: *Micantes*, *Mastichina*, *Piperella*, *Teucrioides*, *Pseudothymbra*, *Thymus*, *Hyphodromi,* and *Serpyllum*. Five of them (sections *Micantes*, *Mastichina*, *Piperella*, *Pseudothymbra,* and *Teucrioides*) are endemic to the West Mediterranean area (Iberian Peninsula, Northwest Africa) [72].

## 4. *Thymus algeriensis* Boiss. and Reut.

### 4.1. Distribution

**Figure 1:**
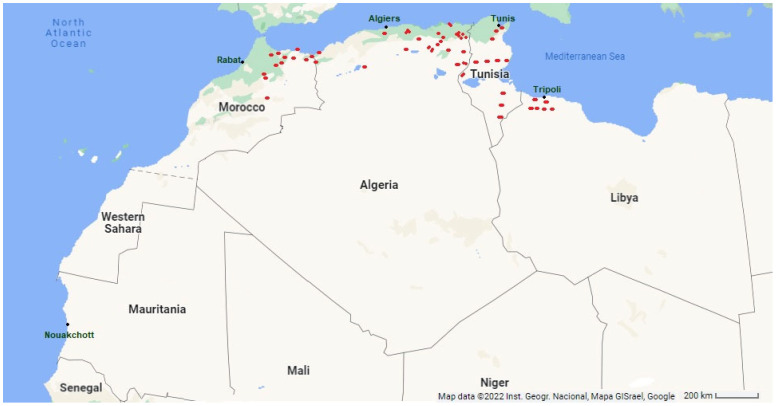
In Red *Thymus algeriensis* Boiss. and Reut. Maghreb distribution (Coordinates N26° 14.526120 E5° 9.313440) [43].

The medicinal properties of *Thymus* species have been investigated in numerous scientific studies, including in vitro et in vivo experiments. The results reveal that they have a unique combination of beneficial functions due to the Mediterranean climate [73,74,75]. In this geographical area, the western Mediterranean, under the influence of the Atlantic Ocean, the climatic conditions are favorable for thyme vegetation. Therefore, they are mainly found on the Mediterranean coast [76]. *Thymus* species are sun-loving, heliophilous plants, a fact that reflects the ecology of the genus. *Thymus* plants frequently live on rocks or stones, and the soil must be well-drained [70]. They need very different substrates. *T. algeriensis* usually lives on calcareous soils, characteristic of the Maghreb [77]. The Algerian species are found in the eastern Tell, in the bedrock areas, and on the high mountain plateaus up to the border of the pre-Sahara Tassili [43,78]. They are mainly present in subhumid and arid zones from the North-East of Algeria to the Tunisian border [45]; a few isolated individuals occur in Mascara province and from the Oran region to the Moroccan borders (Figure 1).

The Moroccan *T. algeriensis* is found in the Mediterranean part of the country, in the Tangier-Tetouan-Al Hoceïma and Fez-Meknes regions, and more particularly in the so-called Oriental area (Béni Snassen forest), in the northeast near the Algerian border [43,78,79]. A few specimens have been found in the Middle Atlas, in the so-called Béni Mellal-Khénifra region (Figure 1). In Tunisia, the species is found in almost all bioclimates. It occurs in the north-eastern Mediterranean in the sub-humid zones of the Boukournine Mountains at 200 m altitude, up to the upper semi-arid zones at the foot of the Reças Mountains (Lead Mountain) at 150 m altitude and the Zaghouane Mountains at about 300 m altitude (Figure 1). Many populations of *T. Algerians* have been observed in the lower arid bioclimate. They formed a kind of vegetative belt from the city of Sfax to the Magel Bel Abbes near the Algerian border. In the southeastern part of the country, however, it occurs in the upper and lower Saharan bioclimate regions of Ksar Jedid and Remada [43,78]. In the Libyan zone, *T. algeriensis* only appears in the north-western part of the country (Figure 1), specifically in the Mediterranean coastal bioclimate and the coastal steppes climate in Qsar bin ghashir and Abu ar Rish. In the highlands, it has been detected in the Beni Walid, El Urban, Al Urqub, and Rahiba regions [43,78].

### 4.2. Systematic Classification and Botanical Aspects

**Figure 2:**
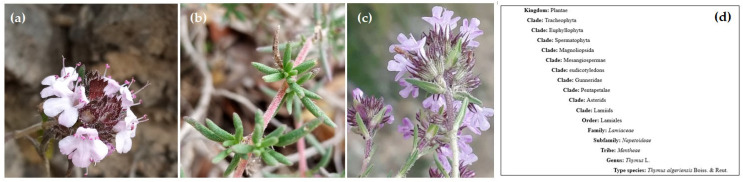
Systematic classification and botanical aspects of *Thymus algeriensis* Boiss. and Reut flowers and leaves. (**a**,**b**) from Tunisia, (**c**) from Algeria, (**d**) Systematic classification of *T. algeriensis* [82].

According to Morales [65], *T. algeriensis* Boiss. and Reut. are classified in section *Hyphodromi* (A. Kerner) Haläcsy and subsection *Subbracteati* (Klokov) Jalas [80,81]. It belongs to the order Lamiale, subfamily Nepetoideae, and tribe Menthae (Figure 2).

*T. algeriensis* is a subshrub that can reach a height of 50 cm. It is a short-lived diploid species (2*n* = 2x = 30), which is also aromatic, perennial, and gynodioic [80,83,84] It is characterized by small, dark green, opposite, lanceolate, short petiole leaves. It reproduces by seed through vegetative means. *T. algeriensis* is hermaphroditic (male fertile) or female (male sterile) [83]. Pollination is usually via bees (allogamous species). Self-pollination can also occur in hermaphrodite plants [85]. The vegetative stage occurs in January and February, and its flowering occurs between April and June [86]. Its flowers are small (5–6 mm) with a glandular calyx, oval-shaped bracteoles, and a pinkish-purple corolla.

### 4.3. Uses in Folk Medecine

In traditional Algerian medicine, *T. algeriensis* has been used as an astringent, expectorant, and healing agent and a blood circulation stimulant and aphrodisiac [87,88]. Infusion, decoction, and powder of the aerial parts are used in Naâma, southwest Algeria for treating colds as an anti-inflammatory, to manage hypercholesterolemia and menstrual cycle problems, and recently against COVID-19 [34]. El Kantara’s area (Algerian Sahara gate) is traditionally employed to flavor coffee, buttermilk, and tea. Infusing leaves and flowers are used against abdominal stomach pain, wound infections, and food poisoning. It is also antihypertensive and manages heart diseases [89]. In Morocco, *T. algeriensis* is a medicinal species indicated in the traditional treatment of diabetes [90]. It is a tonic stimulant against cough, fever, and wound infections [91,92]. It also treats asthma, bad breath, chest pain, lung disorders, and rhinosinusitis. In addition, some of his by-products were used as antitussives [33] and anti-inflammatory agents by topical or oral administration [93,94,95]. Traditionally in Tunisia, *T. algeriensis* is used as a culinary herb, fresh or dried [96], or as condiments or flavoring mainly added to black tea [35]. It is widely used in popular Tunisian medicine as a protective treatment against digestive tract diseases and abortion [97].

### 4.4. Phytochemistry

#### 4.4.1. Essential oil Chemical Composition

The species belonging to the Lamiaceae family are reservoirs of molecules with high chemical diversity [12,98,99,100,101]. Many compounds have been identified to understand the relationship between their structures with biological activities and therapeutic potential. This significant chemical variability is mainly present in the species of the genus *Thymus* and also within the same species depending on the environmental, plant, and soil characteristics in which they grow [81,102,103]. These are several horticultural species, most of which are used as culinary herbs to flavor food. The flavoring comes from the fact that they produce essential oils with particular spicy aromas and flavors that are in demand by the cosmetic, nutrition, and health industries [104,105,106,107,108].

Essential oils are colorless and lipophilic liquids. They are odoriferous as a combination of volatile compounds derived from the secondary metabolism of plants [109]. They are mainly monoterpenes, sesquiterpenes, and diterpenes. Phenylpropanoids, fatty acids and their esters, alcohols, acids, epoxides, aldehydes, ketones, amines, and sulphides were also identified [110,111]. According to Venditti et al. [112], these compounds can be accompanied by polysaccharides and polyphenols.

Plants synthesize and emit a wide variety of volatile organic compounds that act as chemical signals, controlling their external environment. Stresses such as pathogen infections [113] or subterranean microbial communities [114] can induce the particular emission of volatile compounds from plants. They attract insect pollinators and repel predators [109]. Some of these signals are generated and emitted directly after herbivores damage plants. The volatile substances released often serve as indirect defenses, attracting insects and mites. The last attack or parasitize the herbivores and thus reduce further damage to the plant [115]. Additionally, essential oils inhibit seed germination and help plants communicate with each other. Indeed, several scientific reports have shown that plants can perceive other volatile substances emitted by nearby plants under attack by herbivores [116,117,118]. They respond to the information emitted by activating their defenses. In some cases, high expression of several genes involved in defense metabolism has been observed [119,120,121].

All species of *Thymus* produce essential oils [81]. They synthesize and store them in specialized anatomical structures called glandular trichomes [122]. These epidermal outgrowths contain special secretory cells heterogeneously distributed on all parts of the plant as follows: flowers, buds, seeds, leaves, twigs, bark, herbs, wood, fruits, and roots [123]. In the study of Guesmi et al. [86], scanning electron microscopy analysis demonstrated that *T. algeriensis* leaves contain glandular and non-glandular trichomes. Classifications can be based on the following two points: the cell number and the configuration of their walls in the head of the gland [124]. The glandular trichomes of *T. algeriensis* are of the peltate and capitate types. In peltate trichomes, the secretory material accumulates in the subcutaneous space and is finally released by rupturing the cuticle [125]. These structures are considered the sites of essential oil production [126]. In contrast, the secretory material is extruded through the cuticle in capitate trichomes [125]. They can produce essential oils and polysaccharides [126].

Numerous studies have been conducted on *Thymus* species to identify their chemical composition. The outcome is that they are rich in essential oils, characterized by a remarkable variability in chemical composition [127,128]. Oil chemotypes comprise monoterpenes, sesquiterpenes, and their oxygenated and hydrocarbon derivatives. An essential oil may contain one, two, or three of these main types of compounds and thus constitute a particular chemotype [129].

**Figure 3:**
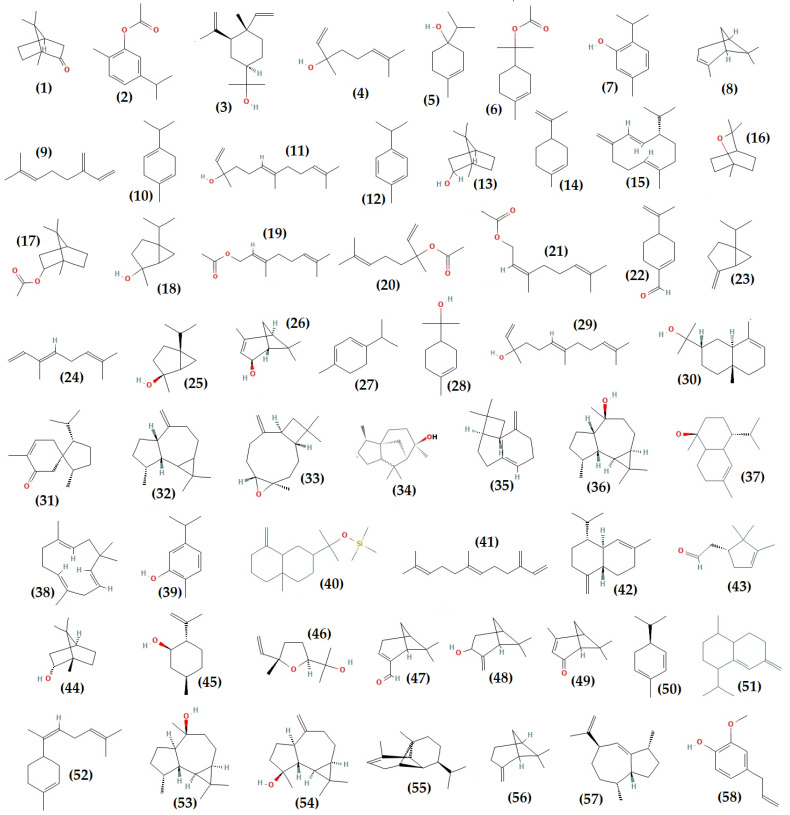
Chemical structures of the phenolic and volatile compounds (1–58) identified from *Thymus algeriensis* Boiss. and Reut: camphor (**1**), carvacrol acetate (**2**), elemol (**3**), linalool (**4**), terpinene-4-ol (**5**), *α*-terpinyl acetate (**6**), thymol (**7**), *α*-pinene (**8**), *β*-myrcene (**9**), *γ*-terpinene (**10**), (trans)-nerolidol (**11**), cymene (**12**), borneol (**13**), limonene (**14**), germacrene d (**15**), 1,8-cineol (**16**), bornyl acetate (**17**), *cis*-sabinene hydrate (**18**), geranyl acetate (**19**), linalyl acetate (**20**), neryl acetate (**21**), perilla aldehyde (**22**), sabinene (**23**), trans-ocimene (**24**), trans-sabinene hydrate (**25**), trans-verbenol (**26**), *α*-terpinene (**27**), *α*-terpineol (**28**), (e)-nerolidol (**29**), 7-epi-*α*-eudesmol (**30**), acorenone (**31**), allo-aromadendrene (**32**), caryophyllene oxide (**33**), 5-neo-cedranol (**34**), *trans*-caryophyllene(**35**), viridiflorol (**36**), *α*-cadinol (**37**), *α*-caryophyllene (**38**), carvacrol (**39**), *β*-eudesmol (**40**), *β*-farnesene (**41**), *δ*-cadinene (**42**), campholenal (**43**), *endo*-borneol (**44**), *iso*-pulegol (**45**), linalool oxide (**46**), myrtenal (**47**), pinocarveol (**48**), verbenone (**49**), *α*-phellandrene (**50**), (+)-*epi*-bicyclosesquiphellandrene (**51**), *cis*-*α*-bisabolene (**52**), epiglobulol (**53**), spathulenol (**54**), *α*-copaene (**55**), *β*- pinene (**56**), *γ*-gurjunene (**57**), *p*-eugenol (**58**).

The structures of some (58) of the volatile and phenolic compounds identified in the essential oils of *T. algeriensis* are shown in Figure 3. GC-MS allowed the identification of an average of 43 volatile compounds in the Algerian *T. algeriensis* essential oils. They are mainly monoterpenes and sesquiterpenes responsible for the perfume [51,54,55,61,130,131,132,133,134,135,136,137]. Camphor, carvacrol acetate, elemol, linalool terpinene-4-ol, α-terpinyl acetate, thymol, α-pinene, β-myrcene, and γ-terpinene are the main constituents of Algerian essential oils, extracted by hydrodistillation, steam distillation, or by microwave (Table 1 and Figure 3). They are primarily found in the aerial parts of the plants. According to their location, there are three categories of essential oils. Based on major compounds, the populations of the northern region (Blida, Ain Defla, Medea) are characterized by the chemotypes linalool, terpinene-4-ol/camphor, terpinyl acetate/(*trans*)-nerolidol, thymol, and γ-terpinene/cymene [134,135,136,137]. The *T. algeriensis* of central regions (M’sila, Laghouat) contains camphor, camphor/borneol, carvacrol acetate/limonene, and α-terpinyl acetate as the main chemotypes [61,130,132,138]. Camphor, elemol/camphor, β-myrcene/camphor, germacrene D, and α-pinene are the chemotypes of the eastern region plants (Batna, Biskra, Guelma, Oum El Bouaghi, Soukahras) [51,53,54,55,131,139,140]. Monoterpenes such as 1,8-cineole, bornyl acetate, *cis*-sabinene hydrate, geranyl acetate, linalyl acetate, neryl acetate, perilla aldehyde, sabinene, *trans*-ocimene, *trans*-sabinene hydrate, *trans*-verbenol, α-terpinene, and α-terpineol have also been identified (Figure 3). Sesquiterpenes like the-nerolidol, 7-epi-α-eudesmol, acorenone, allo-aromadendrene, caryophyllene oxide, 5-neo-Cedranol, *trans*-caryophyllene, viridiflorol, α-cadinol, α-caryophyllene, β-caryophyllene, β-eudesmol, β-farnesene, and δ-cadinene are among the volatile oil components detected in Algerian oils (Figure 3).

According to this research (Table 1), there is a clear chemical variability between specimens of *T. algeriensis* collected in the different Algerian regional groups. These include the North (bioclimate Mediterranean sub-humid) [134,135,136,137], the East (Mediterranean sub-humid, arid to super arid bioclimate) [51,53,54,55,131,139,140] and the Centre (super arid, sub-Saharan to Saharan bioclimate) [60,130,132]. It is even present between provinces belonging to the same bioclimatic group. The chemical composition of the essential oils of *T. algeriensis* collected in the Aurès region of Algeria is one example among many others. It is unique compared to other oils from the same region. Indeed, it is characterized by its high sesquiterpene content (67.0%). The main compounds of the oil were germacrene D (29.6%), *β*-caryophyllene (11.0%), and E-*β*-farnesene (7.8 According to Kebbi et al. [140], this is the first time that these compounds have been described with high content in *T. algeriensis* oil.

Furthermore, the essential oils of *T. algeriensis* showed different chemical profiles even for samples taken from the exact location at different levels. In the study by Hazzit et al. [135] the sample collected at 800 m altitude in Chrea National Park is characterized by thymol as the predominant component. In contrast, the sample taken at the same location at 1500 m altitude showed a predominance of terpinyl acetate/nerolidol/α-pinene/borneol/bornyl acetate [135]. Touhami et al. [141] also reported a difference in the chemical composition according to the developmental stage. The plants collected at the flowering stage had high proportions of oxygenated monoterpenes (77.56%) and oxygenated sesquiterpenes (5.98%). On the other hand, at the pre-flowering stage, their levels were lower (61.86%) and (2.10%), respectively. There was also a decrease in the monoterpenes (25.36%) and sesquiterpenes (10.68%) from the pre-blooming stage (16.46%) to the blooming stage (0.00%), respectively. According to Touhami et al. [141], these changes may be due to an increase in photosynthetic activity that induces a high rate of biosynthesis of volatile compounds (mainly phenolic compounds) at the complete flowering stage. Indeed, various studies have shown that the secretion of essential oils and their chemical composition in volatile compounds depends on several factors. They can be the development stage of the plant, its age, plant organ, species, seasonality, circadian rhythms, geographical location, and genetics [142,143,144,145,146]. Other factors related to the harvesting period [147], storage conditions [148], and extraction technique [149] also influence the nature of the essential oil.

Monoterpenes (1,8-cineole, camphor, *cis*-sabinene hydrate, linalool, terpinen-4-ol, terpinyl acetate, *α*-pinene), phenol-terpene thymol, and sesquiterpenes (caryophyllene oxide, viridiflorol) are the main compounds identified in the different Tunisian populations of *T. algeriensis* (Table 1). Some chemotypes are similar to those of the Algerian oils, such as camphor [49,143], linalool [48,85,150,151], thymol [85], and *α*-pinene [85,152]. Others are different such as 1,8-cineole, 1,8-cineole/camphor, 1,8-cineole/*α*-pinene, 4-terpineol/camphor, camphor/4-terpineol, caryophyllene oxide, *cis*-sabinene hydrate, *cis*-sabinene hydrate/1,8-cineole, eucalyptol, eucalyptol/2-carene, eucalyptol/viridiflorol, terpinen-4-ol, terpinyl acetate/1,8-cineole, viridiflorol, viridiflorol/cyclo-hexene,1-(1-butenyl), and viridiflorol/*α*-pinene [48,50,86,143,150,151,152,153].

Monoterpenes (2-carene, borneol, bornyl acetate, camphene, campholenal, *cis*-sabinene hydrate, *endo*-borneol, geraniol, *iso*-pulegol, linalool oxide, linalyl acetate, myrtenal, *o*-cymene, *p*-cymene, pinocarveol, sabinene, terpinyl acetate, verbenone, *α*-phellandrene, *α*-terpineol, *α*-terpinylacetate, *β*-ocimene, *β*-phellandrene, *β*-pinene, and *γ*-terpinene) have been identified in Tunisian populations of *T. algeriensis* (Figure 3). The (+)-*epi*-bicyclosesquiphellandrene, *allo*-aromadendrene, *cis*-*α*-bisabolene, elemol, epiglobulol, germacrene B, ledol, spathulenol, *α*-cadinol, *α*-copaene, *α*-humulene, *β*-caryophyllene, *β*-eudesmol, *γ*-cadinene, and *γ*-gurjunene are the sesquiterpenoid compounds that have also been detected in plants [48,50,86,143,150,151,152,153]. The phenolic compound carvacrol was only seen at 2.55% in the flowers and leaves of a species at the vegetative and flowering stage in the Oued Oum Ali region [143]. Furthermore, the phenylpropanoid compound, *p*-Eugenol (Figure 3), was only present in the leaves (tr-14.40%) and roots (tr-15.80%) of a population collected in Korbous, Jdidi Jebel Mountain, and Hammem Sousse [152].

**Table 1:** Main components of the essential oils isolated from different Maghreb populations of *Thymus algeriensis* Boiss. and Reut (Algeria, Libya, Morocco, and Tunisia).

R/P *	PP	P/H	S	EX	NC	MC	Ref.
Algeria							
Biskra province	AP	n.m, Apr	Eo	HDGC-MS	34	Camphor (37.29%); 1,8-Cineole (11.12%); Camphene (7.81%); Myrcene (7.13%); Borneol (5.54%); Limonene (3.44%); Germacrene-D (2.31%); *β*-Caryophyllene (2.30%)	[51]
El Hadjeub and El Ghicha/Laghouat	n,m	n.m, Jun	Eo	HDGC-FID, GC-MS	n.m	Carvacrol acetate (14.16%); Limonene (11.49%); *α*-Pinene (9.26%)	[130]
El-Guetfa/M’sila	AP (S, L, F) L	BFlo Flo Aflo Flo, Mar	(1) BFEo (2) Feo (3) AFEo (4) LMAD (5) LHD (6) LSD	HD, GC-MS, GC-FID HD, GC-MS, GC-FID HD, GC-MS, GC-FID HD, SD, MAD GC-FID, GC-MS	85	BFEo: Camphor (17.45%); Borneol (13.90%); Camphene (10.73%); Acorenone (8.03%); 1,8-Cineole (5.16%); *α*-Pinene (4.56%); Geranyl acetate (4.26%); *α*-Cadinol (4.14%); Bornyl acetate (3.86%); *trans*-Sabinene hydrate (2.40%) Feo: Camphor (22.60%); Camphene (12.75%); Borneol (11.16%); 1,8-Cineole (5.94%); Acorenone (5.85%); *α*-Pinene (5.00%); Bornyl acetate (3.86%); Geranyl acetate (2.65%); 7-*epi*-*α*-Eudesmol (2.63%) AFEo: Camphor (34.31%); Borneol (14.48%); Camphene (12.86%); 1,8-Cineole (11.21%); Bornyl acetate (4.278%); *α*-Pinene (2.80%) LMAD: Camphor (20.74%); Borneol (16.74%); Camphene (8.73%); 1,8-Cineole (7.01%); 5-neo-cedranol (6.03%); Bornyl acetate (5.70%); 7-*epi*-*α*-Eudesmol (4.13%); Geranyl acetate (3.78%); *α*-Pinene (3.03%) LHD: Camphor (32.56%); Borneol (17.13%); Camphene (14.88%); 1,8-Cineole (7.88%); Bornyl acetate (5.21%); *α*-Pinene (3.74%) LSD: Camphor (24.25%); Borneol (22.20%); Perilla aldehyde (13.21%); Bornyl acetate (7.92%); 1,8-Cineole (7.72%); Camphene (7.53%)	[61]
El Kantara area/Biskra	AP	n.m, Apr	Eo	HD GC/MS	35	Camphor (52.16%); Borneol (12.72%); L-*α*-terpineol (5.46%); Terpinen-4-ol (4.04%); Germacrene D (3.94%); Linalool (3.71%); Bornyl acetate (2.57%); Caryophyllene (2.55%)	[54]
Aures/Batna	AP	n.m/Jun	Eo	SD GC-MS, GC-FID	35	Germacrene D (29.60%); β-Caryophyllene (11.00%); -*β*-Farnesene (7.80%); *β*-Eudesmol (5.30%); *δ*-Cadinene (4.00%); Bicyclogermacrene (4.40%); *α*-Humulene (3.50%); *α*-Guaiene (2.30%); *α*-Bulnesene (2.40%); E-Nerolidol (2.40%); Phytol (2.30%)	[140]
Djemorah/Biskra	S, L, F, Fr	Flo, Apr/Ma	Eo	HS-SPME GC-MS	39	β-Myrcene (13.78%); Camphor (12.29%); Linalyl acetate (9.11%); 1,8-Cineole (6.31%); *β*-Farnesene (5.23%); *α*-Terpineol (5.07%); Camphene (4.61%); *α*-Pinene (4.65%); Bornyl acetate (4.79%)	[131]
Laghouat province	L	Flo, Apr/Ma	Eo	HD GC-MS	29	α-Terpinyl acetate (47.40%); Neryl acetate (9.60%); *α*-Pinene (6.80%); *α*-Terpineol (4.90%); 1,8-Cineole (4.10%); Nerolidol (3.5%); Bornyl acetate (3.1%); Limonene (2.70%)	[132]
National Park of Bellazma/Batna	S, L, F	Flo, Mar/Apr	Eo	SD GC-MS, GC-FID	30	Elemol (18.38%); Camphor (14.22%); *β*-Eudesmol (11.50%); *α*-Caryophyllene (9.68%); Borneol (6.44%); Germacrene isomer (4.55%); Caryophyllene oxide (3.51%); Bornyl acetate (2.41%)	[55]
Ain Beida/Oum El Bouaghi	S, F, L, Fr	Flo, n.m	Eo	HD GC-MS	36	Camphor (13.62%); 1,8-Cineole (6.00%); Borneol (5.74%); Viridiflorol (4.00%); Linalool (3.93%); *α*-Terpineol (3.80%); Caryophyllene oxide (3.50%)	[139]
Mekhatri/Ain-Defla	AP	n.m, Jun	Eo	HD GC-MS, GC-FID	34	*γ*-Terpinene (14.90 ± 2.80%); Cymene (14.70 ± 2.60%); Carvacrol (8.40 ± 4.20%); Thymol (5.60 ± 1.80%); *β*-Myrcene (2.70 ± 0.90%)	[134]
Selaoua Anouna/Guelma	L, F	Pflo, Mar/Apr Flo, Ma/Jun	SAPFlo SAFlo	HD GC-MS	19	SAPFlo: Camphor (33.30%); O-Cymene (6.36%); Isolimonene (5.50%); Eucalyptol (5.31%); Limonene (5.13%); Linalool (4.68%) SAFlo: Verbenone (13.18%); *p*-Cimene-7-ol (26.98%); Methyl ter buthy ether (19.63%); *β*-Cymene (7.74%); *γ*-Terpinene (5.64%); Camphor (3.64%); *α*-Pinene (3.08%); Pinocarveol (2.67%); Mertenyl acetate (2.59%)	[154]
Sidi Aissa/M’sila	AP (L, F, S)	Flo, Apr	Eo	HD GC-MS, GC-FID	71	Camphor (22.61%); Camphene (12.78%); Borneol (11.16%); 1,8-Cineole (5.94%); Acorenone (5.84%); *α*- Pinene (5.01%); Bornyl acetate (3.86%); Geranyl acetate (2.65%); 7-*epi-α* -Eudesmol (2.63%);	[138]
Chrea National Park/Blida 800 m altitude (CHR1) Chrea National Park/Blida 1500 m altitude (CH2) El-Asnam/Blida (ALAS)	AP	Flo, July	(1) CHR1Eo (2) CHR2Eo (3) ALASEo	HD GC-MS, GC-FID	53 49 46	CHR1Eo: Thymol (29.50%); *p*-Cymene (13.00%); *γ*-Terpinene (6.90%); *α*-Pinene (5.80%); *β*-Caryophyllene (5.00%); Caryophyllene oxide (5.00%); *β*-Pinene (3.70%); Linalool (3.60%); Carvacrol (3.30%) CHR2Eo: Terpinyl acetate (18.00%); (*trans*)-Nerolidol (12.60%); *α*-Pinene (11.10%); Borneol (9.00%); Bornyl acetate (7.70%); Camphene (5.90%); *β*-Pinene (3.20%); Limonene (2.80%); Camphor (2.30%) ALASEo: Terpinen-4-ol (10.60%); Camphor (10.10%); *p*-Cymene (9.90%); *α*-Pinene (6.50%); 1,8-Cineole (6.50%); *γ*-Terpinene (5.50%); Caryophyllene oxide 3.90%); *trans*-Verbenol (3.60%); *α*-Terpinene (2.80%); Camphene (2.30%); *cis*-Sabinene hydrate (2.30%)	[135]
Khedara/Soukaharas (KH) Fatoum Souda/Soukaharas (FAT)	L	Flo, Mar	(1) KHEo (2) FATEo	HD GC-MS	54	KHEo: *α*-Pinene (27.14%); Camphor (8.77%); 1,8-Cineole (7.69%); Sabinene (5.25%); *δ*-Cadinene (3.39%); *Allo*-Aromadendrene (3.12%); *trans*-Ocimene (2.84%); β-Pinene (2.66%); β-Caryophyllene (2.54%); Limonene (2.41%); Borneol (2.40%) FATEo: α-Pinene (25.52%); Camphor (8.45%); 1,8-Cineole (7.68%); Sabinene (5.61%); Allo-Aromadendrene (3.52%); *δ*-Cadinene (3.14%); *β*-Pinene (3.12%); Limonene (2.46%)	[53]
Media province	AP	Flo, Ma	Eo	HD GC-MS	55	Linalool (47.30%); Thymol (29.20%); *p*-Cymene (6.80%); *β*-Caryophyllene (2.90%)	[136]
Blida province	n.m	n.m	Eo	HD GC-MS, GC-FID	25	Linalool (40.20%); Thymol (33.70%); *p*-Cymene (5.50%); *γ*-Terpinene (3.20%); *β*-Caryophyllene (2.70%)	[137]
**Tunisia**							
Mount Orbata/Gafsa	AP	Flo, Apr	Eo	HD GC-MS	52	Viridiflorol (9.72%); Cyclo-hexene, 1-(1-butenyl) (9.71%); *iso*-Pulegol (8.27%); α-Terpinylacetate (4.93%) Camphre (4.89%); Terpinen-4-ol (4.50%); *β*-Ocimene (4.11%); 6-ethenyl-6,9,9-trimethyl-4-methylidenebicyclo [5,2,0] nonane (3.12%); 1-Borneol (3.07%); *β*-Phellandrene (2.94%); Camphene (2.90%)	[50]
Mount Orbata/Gafsa	AP	n,m	Eo	HD GC-MS, FTIR	n.m	Thymol; (+)-*epi*-bicyclosesquiphellandrene; Ledol; Camphor; Linalool; 2-Carene; Terpinen-4-ol; *Endo*-borneol; Eucalyptol; *α*-Pinene	[153]
Mount Orbata/Gafsa	AP	L (Veg, Jan) L, F (Flo, Mar) L (Frui, Apr, Ma)	(1) TeoBF (2) TeoF (3) TeoAF	HD GC-MS	32 46 43	TeoBF: Eucalyptol (13.37%); *Endo*-Borneol (9.45%); *α*-Pinene (8.13%); Camphor (6.50%); Terpinen-4-ol (3.99%); β-Pinene (2.72%); Linalyl acetate (2.70%); Camphene (2.65%) TeoF: Eucalyptol (9.30%); 2-Carene (6.42%); Linalool (6.08%); Terpinen-4-ol (6.10%); Camphor (5.92%); Viridiflorol (4.52%); Linalool oxide (2.57%); *α*-Terpineol (3.41%) TeoAF: Eucalyptol (10.34%); Viridiflorol (8.69%); Camphor (8.23%); Terpinen-4-ol (6.14%); *endo*-Borneol (4.93%); Thymol (4.01%); *α*-Pinene (3.48%); *β*-Pinene (2.51%)	[86]
Korbous (KOR) Jdidi Jebel Mountain (JDID) Hammem Sousse (HAM)	L, S, R	Veg, n,m	(1) EoR (KOR, JDID, HAM) (2) EoS (KOR, JDID, HAM) (3) EoL (KOR, JDID, HAM)	HD GC-MS	35 46 48	EoR: Viridiflorol (tr–39.70%); Caryophyllene oxide (18.50–25.30%); *α*-Pinene (2.70–15.20%); 1,8-Cineole (1.20–12.80%); *p*-Eugenol (tr–15.80%); Geraniol (tr–7.10%); *cis*-*α*-bisabolene (tr–10.60%) EoS: Caryophyllene oxide (9.70–24.20%); Elemol (8.10–13.10%); Viridiflorol (6.40–9.00%); Camphor (2.00–10.30%); *α*-Pinene (5.80–8.80%); Linalyl acetate (tr–7.20%); *γ*-Gurjunene (tr–7.20%) EoL: *α*-Pinene (13.60–23.20%); 1,8-Cineole (7.40–17.80%); Caryophyllene oxide (4.30–17.80%); Camphor (4.10–14.80%); Linalool (3.20–14.50%); Camphene (2.70–5.90%); *p*-Eugenol (tr–14.40%).	[152]
Mount Orbata/Gafsa	AP	n,m	Eo	SD GC-MS	13	Linalool (18.05%); Camphor (13.03%); Terpinen-4-ol (11.20%); Viridiflorol (11.71%); Bornyl acetate (5.41%); 1,8-Cineole (3.45%); *p*-Cymene (3.22%); Spathulenol (2.80%); γ-Terpinene (2.43%);	[150]
Gafsa (MG) Tamerza (MT) Kairouan (MOK)	AP	Flo, Mar	(1) MGEo (2) MTEo (3) MOKEo	HD GC-MS	25	MGEo: Terpinen-4-ol (33.34%); 1,8-Cineole (14.12%) MTEo: Linalool (18.05%); Camphor (13.03%) MOKEo: 1,8-Cineole (19.96%); Camphor (19.20%)	[48]
Zannouch (ZAN) Oued Om Ali (OUE) Ayaycha (AYA) Sidi Harrath (SID) Dachra (DAC) Djebel Slata (DJE) Haydra (HAY) Kalaat Senan (KAL)	F, L	Veg, Dec Flo, Apr	ZAN (Veg, Flo) OUE (Veg, Flo) AYA (Veg, Flo) SID (Veg, Flo) DAC (Veg, Flo) DJE (Veg, Flo) HAY (Veg, Flo) KAL (Veg, Flo)	HD GC-FID, GC-MS	63 61 58 59 58 57 49 49 44 48 48 48 43 60 39 48	ZAN-Veg: 1,8-Cineole (10.91%); *α*-Pinene (10.49%); Camphor (10.23%); Borneol (4.58%); 4-Terpineol (4.36%); Camphene (3.84%); Viridiflorol (3.62%); Sabinene (3.37%); Linalool (2.95%); cis -Sabinene hydrate (2.83%); β-Pinene (2.78%); Bornyl acetate (2.32%) ZAN-Flo: 1,8-Cineole (15.79%); α-Pinene (9.68%); Camphor (9.40%); Borneol (5.19%); 4-Terpineol (4.57%); Viridiflorol (4.24%); Camphene (3.89%); Bornyl acetate (3.28%); Linalool (2.69%) OUE-Veg: *cis*-Sabinene hydrate (9.86%); 1,8-Cineole (7.55%); *α*-Pinene (7.41%); Camphor (6.80%); Viridiflorol (5.69%); 4-Terpineol (5.30%); *β*-Pinene (4.03%); *α*-Cadinol (3.58%); Borneol (3.47%); Camphene (3.22%); Sabinene (3.15%); *γ*-Terpinene (3.15%); Carvacrol (2.55%); *γ*-Cadinene (2.44%) OUE-Flo: Viridiflorol (11.49%); *α*-Pinene (9.80%); 1,8-Cineole (8.73%); Camphor (8.17%); Sabinene (4.40%); *β*-Pinene (4.29%); Camphene (3.51%); α-Cadinol (3.4%); Borneol (3.33%); 4-Terpineol (3.32%); Caryophyllene oxide (2.66%); Bornyl acetate (2.61%); *cis*-Sabinene hydrate (2.59%); *γ*-Cadinene (2.58%) AYA-Veg: *cis*-Sabinene hydrate (12.95%); Camphor (9.93%); 1,8-Cineole (9.00%); *α*-Pinene (8.97%); 4-Terpineol (8.34%); Borneol (4.09%); Camphene (3.48%); Sabinene (2.90%); *β*-Pinene (2.86%); *γ*-Terpinene (2.65%) AYA-Flo: 4-Terpineol (11.86%); Camphor (11.72%); 1,8-Cineole (10.87%); *α*-Pinene (5.60%); *γ*-Terpinene (5.42%); *p*-Cymene (4.2%); Borneol (4.18%); Camphene (4.16%); *α*-Terpinene (3.48%); Viridiflorol (3.25%); *cis*-Sabinene hydrate (2.79%); Bornyl acetate (2.60%) SID-Veg: 1,8-Cineole (18.02%); Camphor (12.02%); Terpinyl acetate (8.88%); Borneol (6.86%); *α*-Pinene (6.58%); Bornyl acetate (4.36%); Camphene (4.11%); Caryophyllene oxide (3.90%); 4-Terpineol (2.87%); Myrtenal (2.40%) SID-Flo: Terpinyl acetate (14.92%); 1.8-Cineole (13.82%); Camphor (8.16%); Bornyl acetate (7.56%); Caryophyllene oxide (5.55%) Borneol (5.40%) DAC-Veg: Camphor (19.39%); 1,8-Cineole (14.44%); *α*-Pinene (9.18%); Camphene (5.59%); Borneol (5.37%); Terpenyl acetate (3.22%); Myrtenal (3.16%); Caryophyllene oxide (2.96%); 4-Terpineol (2.94%); Bornyl acetate (2.88%); Campholenal (2.76%); Verbenone (2.55%) DAC-Flo: 1,8-Cineole (14.73%); Camphor (14.37%); *α*-Pinene (13.25%); Borneol (4.69%); Camphene (4.01%); *β*-Pinene (3.40%); *β*-Eudesmol (2.67%); Caryophyllene oxide (2.30%) DJE-Veg: Camphor (19.93%); 1,8-Cineole (17.90%); *α*-Pinene (11.74%); Borneol (6.21%); Camphene (6.06%); Caryophyllene oxide (3.32%); *β*-Pinene (2.72%); 4-Terpineol (2.70%); Myrtenal (2.62%) DJE-Flo: 1,8-Cineole (18.46%); Camphor (15.69%); *α*-Pinene (10.34%); Borneol (6.14%); Camphene (5.43%); Caryophyllene oxide (3.87%); *β*-Pinene (3.00%); 4-Terpineol (2.38%); Myrtenal (2.30%) HAY-Veg: 1,8-Cineole (22.07%); Camphor (17.49%); *α*-Pinene (13.44%); Camphene (5.58%); Borneol (5.04%); *β*-Pinene (2.37%); 4-Terpineol (2.36%) HAY-Flo: Camphor (13.64%); 1,8-Cineole (12.45%); 4-Terpineol (8.56%); *α*-Pinene (6.38%); Borneol (4.60%); Camphene (4.38%); *p*-Cymene (3.68%); *γ*-Terpinene (3.63%); Bornyl acetate (3.00%) KAL-Veg: 1,8-Cineole (20.48%); Camphor (18.59%); *α*-Pinene (13.94%); Camphene (6.35%); Borneol (5.94%); Caryophyllene oxide (2.72%); Myrtenal (2.69%); Pinocarveol (2.42%); *β*-Pinene (2.41%); Verbenone (2.39%) KAL-Flo: 1,8-Cineole (15.36%); Camphor (14.00%); *α*-Pinene (12.40%); Borneol (4.98%); Camphene (4.94%); Caryophyllene oxide (4.42%); *β*-Pinene (3.22%); *β*-Eudesmol (2.65%); Linalool (2.42%); Myrtenal (2.42%)	[143]
Sabbah Jebel Mountain (SJM) Bahra (BAH) Mansour Jebel Mountain (MJM) Chaambi Jebel Mountain (CHJM) Chrechira Jebel Mountain (CJM) Toujene Matmata (TME) Ouled Bou Saad (OBS) Douaou Jebel Mountain (DJM)	AP	n.m	(1) SJMEo (2) BAHEo (3) MJMEo (4) CHJMEo (5) CJMEo (6) TMEo (7) OBSEo (8) DJMEo	HD GC-MS	25 30 32 38 35 18 32 32	SJMEo: Caryophyllene oxide (18.80%); 1,8-Cineole (15.80%); *α*-Pinene (14.30%); Camphor (9.20%); *allo*-Aromadendrene (5.40%); *α*-Humulene (4.10%) BAHEo: 1.8-Cineole (23.40%); *α*-Pinene (14.30%); Camphor (9.10%); *Allo*-Aromadendrene (4.50%); *γ*-Terpinene (4.80%); *α*-Humulene (4.20%); Camphene (3.30%); Linalool (2.60%) MJMEo: 1,8-Cineole (20.90%); *α*-Pinene (11.30%); Camphor (7.40%); Methyl eugenol (6.90%); Linalyl acetate (6.40%); *allo*-Aromadendrene (5.60%); α-Humulene (5.50%); Camphene (3.90%); *β*-Pinene (2.50%) CHJMEo: 1,8-Cineole (24.10%); α-Pinene (16.90%); Camphor (10.60%); Linalyl acetate (6.40%); Borneol (5.00%); *allo*-Aromadendrene (3.9%); Camphene (3.40%); *γ*-Terpinene (2.50%); Linalool (2.40%); *α*-Humulene (2.40%); *α*-Phellandrene (2.30%) CJMEo: 1,8-Cineole (24.10%); *α*-Pinene (18.40%); Camphor (12.70%); Methyl eugenol (2.50%); Linalyl acetate (6.40%); Borneol (5.00%); *allo*-Aromadendrene (4.20%); Camphene (5.60%); γ-Terpinene (2.90%); *β*-Pinene (2.40%); *α*-Humulene (3.20%); *α*-Phellandrene (2.30%) TMEo: Thymol (54.90%); *p*-Cymene (6.60%); Germacrene B (6.10%); *γ*-Terpinene (6.23%); 1,8-Cineole (4.30%); *α*-Humulene (3.50%); *β*-Caryophyllene (3.10%); *α*-Pinene (2.40%) OBSEo: Linalool (22.40%); 1,8-Cineole (10.10%); *α*-Pinene (9.30%); *α*-Copaene (7.60%); *γ*-Terpinene (6.50%); Camphor (6.00%); Viridiflorol (5.50%) DJMEo: *α*-Pinene (21.50%); 1,8-Cineole (21.20%); Camphor (9.20%); Camphene (4.80%); Viridiflorol (3.40%); *α*-Gurjunene (3.30%); *β*-Pinene (3.2%); Borneol (3%); *allo*-Aromadendrene (3.10%); Sabinene (2.70%); *γ*-Terpinene (2.70%); *α*-Humulene (2.40%)	[85]
Ayaycha mountain/Gafsa	AP	Flo, Apr	Eo	HD GC-MS, FID	57	Camphor (7.82%); 4-Terpineol (7.36%); 1,8-Cineole (5.54%); *cis*-Sabinene hydrate (5.29%); Viridiflorol (3.94%); Linalool (3.65%); *γ*-Terpinene (3.50%); Borneol (3.49%), Camphene (2.88%); *p*-Cymene (2.57%); Sabinene (2.49%); *α*-Terpinene (2.46%); *trans*-*β*-Ocimene (2.40%)	[49]
3end/Gafsa	AP	n.m, Ma	Eo	HD GC-MS; FID	39	Linalool (17.62%); Camphor (13.82%); Terpinen-4-ol (6.80%); *α*-Terpineol (6.41%); *α*-Terpenyl acetate (6.27%); Borneol (5.71%); Linalyl acetate (4.63%); Sabinene hydrate (4.15%); 1,8-Cineole (4.12%); Epiglobulol (3.98%); *o*-Cymene (3.44%); Bornyl acetate (2.61%)	[151]
**Morocco**							
Al Hoceima province	S, L	Flo, Mar/Apr	Eo	HD GC-MS	18	Thymol (33.24%); *γ*-Terpinene (25.23%); *p*-Cymene (13.89%); Carvacrol (7.96%); (+)−4-Carene (4.50%); *α*-Caryophyllene (3.66%); *β*-Myrcene (2.53%); Linalool (2.41%)	[155]
Imizar-Azilal region	S, L, F	Flo, Mar	Eo	HD GC-MS; FID	21	Thymol (46.03%); Borneol (20.38%); Carvacrol (5.86%); *δ*-3-Carene (3.10%); *β*-Ocimene (E) (2.80%); 1,8-Cineole (2.63%); *α*-Terpinene (2.30%)	[63]
Al Hoceima National Park	S, L, F	Flo, Jun	Eo	HD GC-MS	10	Geranyl acetate (80.00%); Geraniol (7.30%); *β*-Caryophyllene (2.40%)	[156]
Oujda	AP	Flo, Mar	Eo	HD GC-MS	41	Borneol (18.30%); Camphene (11.80%); Camphor (10.00%); Geranyl acetate (6.90%); Myrcene (8.60%); *α*-Pinene (6.00%); 1,8-Cineole (4.90%); *β*-Pinene (3.00%); Limonene (3.10%); *p*-Cymene (2.50%)	[157]
Rchida,	S, L, F	Flo, Apr	Eo	HD GC-MS	48	Camphor (27.7%); *α*-Pinene (20.5%); *α*-thujene (9.64%); *β*-Pinene (8.02%); 1,8-Cineole (7.69%); Limonene (4.85%); Sabinene (3.84%)	[52]
Mergchoum Mountain (Taourirt City)	AP	NI	Eo	HD GC-MS	65	Borneol (23.48%); Linalool (8.99%); Camphene (6.90%); Carvacrol (7.76%). *β*-Caryophyllene (6.39%)	[158]
Imizar- Azilal (IAZ) Ait AatabAzilal (AAZ)	L, F	Flo, Jun	(1) IAZEo (2) AAZEo	HD GC-MS	18 10	IAZEo: Carvacrol (80.40%); *p*-Cymene (4.98%); Thymol (3.39%) AZZEo: Carvacrol (49.33%); *p*-Cymene (2.61%)	[56]

* R/P: region/province; PP: part of plant; P/H: plant stage/harvest time; SA: Sample, EX: the extraction method and technic analysis, NC: number of compounds reported; MC: main compounds. AP: arial part; AFlo: after flowering; BFlo: before flowering; Apr: April; Eo: essential oil; EoL: essential oil of leaves; EoR: essential oil of roots; EoS: essential oil of stems; F: flowers; Flo: flowering and reproductive stage; Fr: fruits; Frui: fruiting stage; GC-FID: gas chromatography coupled to a flame ionization detector; GC-MS: gas chromatography–mass spectrometry analysis; HD: hydrodistillation; HS-SPME: solid phase micro-extraction; Jan: January; L: leaves; LFHD: essential oil extracted from leaves (during flowering) by hydrodistillation; LFMAD: essential oil extracted from leaves (during flowering) by microwaves distillation; LFSD: essential oil extracted from leaves (during flowering) by steam distillation; Ma: May; MAD: microwaves distillation; Mar: March; n,m: not mentioned; R: roots; S: stems; SD: steam distillation; TEoAF: Eo of thyme extracted after flowering stage; TEoBF: Eo of thyme extracted before flowering stage; TEOF: Eo of thyme at flowering stage; Veg: vegetative stage.

According to Table 1, a high chemical differentiation among populations was observed. In the study by Guesmi et al. [86], the different developmental phases greatly influenced the chemical composition of the essential oils of *T. algeriensis.* It was found that oils from leaves collected during the vegetative phase contained the highest level of volatile compounds and were at their lowest in older leaves collected in the post-flowering period. Monoterpene hydrocarbons and oxygenated monoterpenes decreased from the vegetative to the fruiting period. In contrast, the content of oxygenated sesquiterpenes was significantly lower during the vegetative stage.

In the studies by Zouari et al. [143], the upper parts of *T. algeriensis* were collected from different locations during the vegetative cycle. The results showed that 18 of the 71 compounds had a statistically significant variation between population locations and phenological stages. The chemical differentiation between the populations observed in the study was high. According to the authors, the distribution of observed chemotypes was related to population location and not to bioclimate, indicating that local selective environmental factors were responsible for the diversity of chemotypes [143].

In the study by Ben-El Hadj Ali et al. [152], qualitative and quantitative differences between different organs and samples were revealed. A large chemical variability was observed in collected plants and oil compound classes. The detection of distinct oil compounds was only in stems (*cis*-sabinene hydrate, *β*-gurjenene, and *α*-humulene), leaves (i.e., verbenone, γ-terpinene), and roots (geraniol and *trans*-*α*-bisabolene). According to the authors, this divergence could be due either to differential oil accumulation or physiological and biochemical interactions within and between organs during morphogenesis. It could also result from differential gene expression of organs and metabolic processes [85].

Nevertheless, this variability has been explained by geographical regions, local abiotic (topography, moisture, temperature, and edaphic factors), and selective biotic factors (associated fauna and flora) as well as genetic factors [152,159]. The latter was evaluated in eight Tunisian populations of *T. algeriensis* using 47 terpenoids and 154 RAPD (random amplified polymorphic DNA) markers amplified by seven selected primers. The populations were collected in different geographical regions of the sub-humid, upper semi-arid, middle semi-arid, lower semi-arid, and upper arid bioclimates. High genetic diversity within populations and high genetic differentiation between them, based on RAPDs, were revealed due to habitat fragmentation, the small size of most populations, and the low level of gene flow between them. Genetic and chemical structures are consistent with geographical distances indicating isolation by distance [85].

Moroccan essential oils are characterized by the chemotype camphor, carvacrol, borneol, thymol, and geranyl acetate (Table 1) [52,56,62,155,156,157,158]. The latter is present but in low levels (from 2.65 to 4.26%) only in the aerial parts (stems, flowers, and leaves) of the Algerian species from the El-Guetfa region (M’sila) and was extracted by hydrodistillation. It is also present in plant leaves but extracted by microwave distillation [61].

#### 4.4.2. Phenolic Compounds

Plants produce an incredible variety of natural products with very different structures. These products are commonly referred to as “secondary metabolites” as opposed to “primary metabolites”, which are essential for plant growth and development [160]. They play a dynamic role in adapting plants to their environment [161]. In addition to their physiological function in plants, natural products also strongly impact human culture and have been used throughout history as condiments, pigments, and pharmaceuticals [7]. In recent years, rapid progress has been made in understanding the genomics and synthesis of natural products, their regulation and function, the evolution of metabolic diversity, and the investigation of the biological activities of secondary metabolites [162,163,164,165]. In addition, interest and sales of alternative therapies and medicines rich in phytochemicals have grown significantly and have been a fast-growing market in all parts of the industrialized world [166].

**Figure 4:**
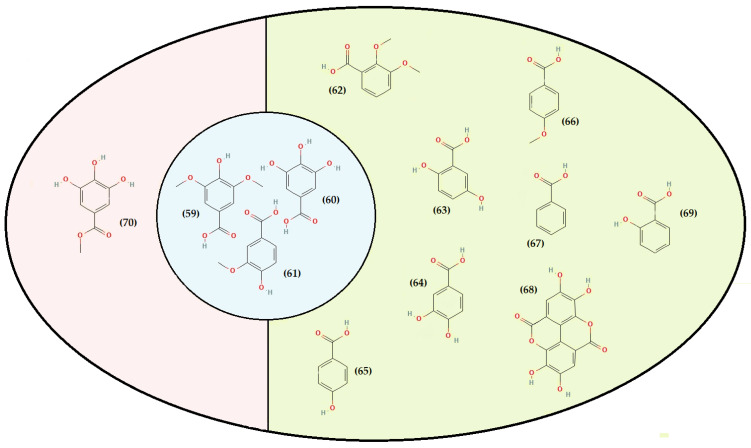
Representation of the hydroxybenzoic acids’ structure identified in *T. algeriensis*. Green from Algeria, red from Tunisia, and blue common between the three countries (Algeria, Tunisia, and Morocco). Syringic acid (**59**), gallic acid (**60**), vanillic acid (**61**), 2,3-dimethoxybenzoic acid (**62**), 2,5 dihydroxybenzoic acid (**63**), 3,4-dihydroxybenzoic acid (**64**), 4-hydroxybenzoic acid (**65**), anisic acid (**66**), benzoic acid (**67**), ellagic acid (**68**), salicylic acid (**69**), and methyl galate (**70**).

**Figure 5:**
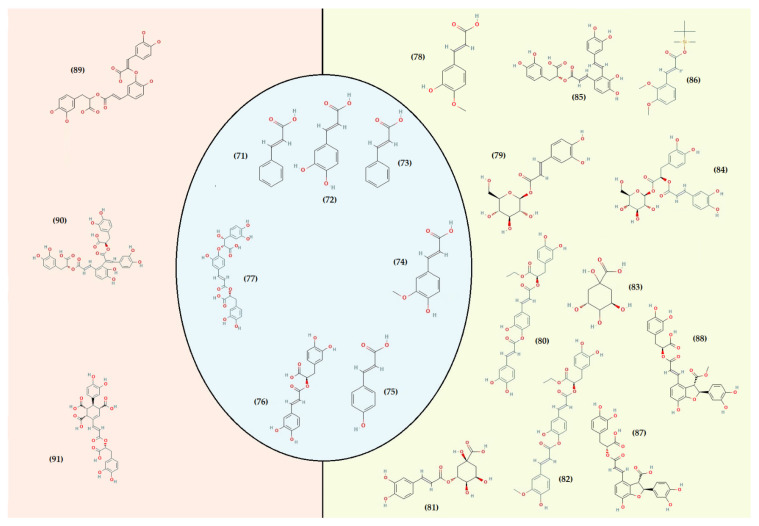
Representation of the hydrocinnamic acid’s structure identified in *T. algeriensis*. Green from Algeria, red from Tunisia, and blue common between the three countries (Algeria, Tunisia, and Morocco). Caffeic acid (**71**), cinnamic acid (**72**), ferulic acid (**73**), p-coumaric acid (**74**), rosmarinic acid (**75**), salvianolic acid K (**76**), *trans*-cinnamic acid (**77**), 3-hydroxy-4-methoxycinnamic acid (**78**), caffeic acid glucoside (**79**), caffeoyl ethylrosmarinate (**80**), chlorogenic acid (**81**), feruloyl ethylrosmarinate (**82**), quinic acid (**83**), rosmarinic acid glucoside (**84**), salvianolic acid A (**85**), *trans*-2.3-dimethoxycinnamic acid (**86**), lithospermic acid A (**87**), monomethyl lithospermate (**88**), caffeoyl rosmarinic acid (**89**), salvianolic acid E (**90**), yunnaneic acid E (**91**).

**Figure 6:**
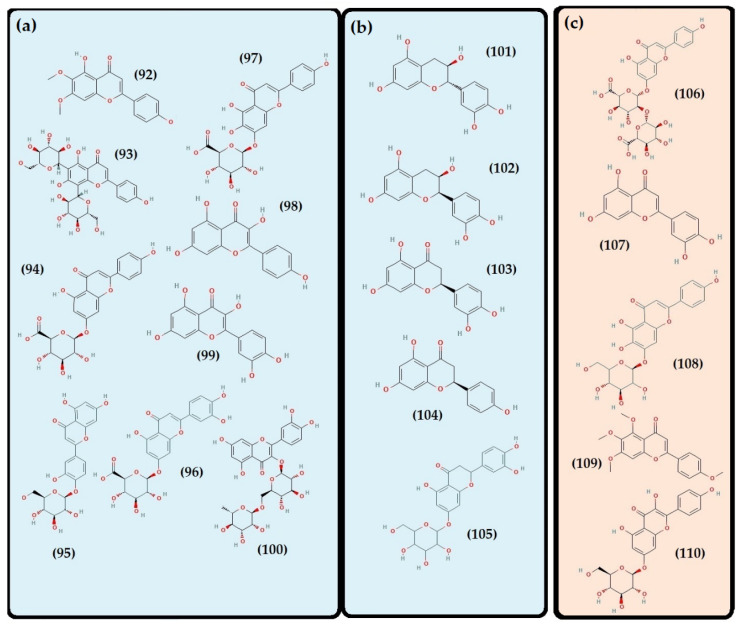

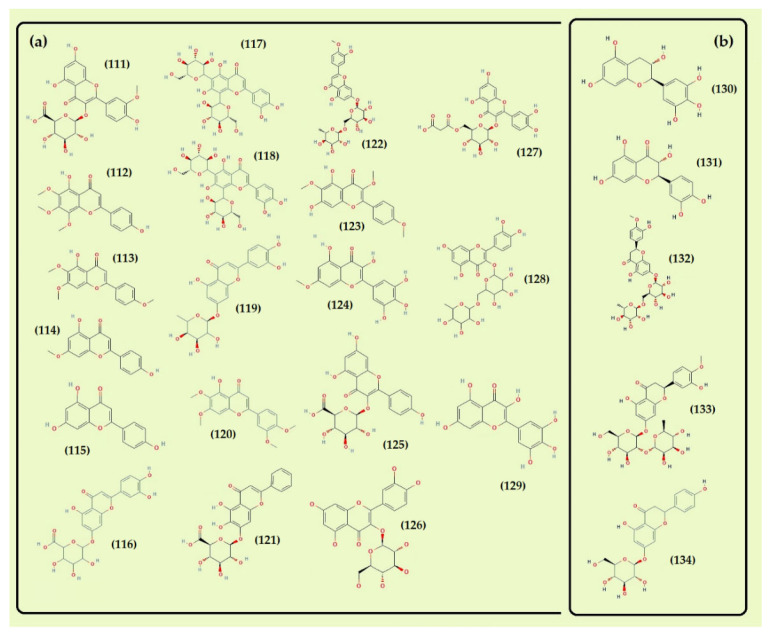
**Part I.** Structure of the flavonoids detected in *Thymus algeriensis* from Maghreb countries. **In blue,** the flavonoids identified in common between the countries. (**a**) Flavone and flavonol compounds: cirsimaritin (**92**), apigenin 6,8-di-C-hexosides, (**93**) apigenin-7-O-glucuronide (**94**), apigenin-8-C-glucoside (**95**), luteolin glucuronide (**96**), scutellarin (**97**), kaempferol (**98**), quercetin (**99**), rutin (**100**). (**b**) Flavanol, flavanone, and flavanone glucoside: catechin (**101**), epicatechin (**102**), eriodictyol (**103**), naringenin (**104**), eriodictyol 7-O-glucoside (**105**). **In red,** flavonoids identified in Tunisian *T. algeriensis*. (**c**) Flavone and flavonol compounds: apigenin diglucuronide (**106**), luteolin (**107**), scutellarein-O-hexoside-hexuronide (**108**), tetramethyl-scutellarein (**109**), kaempferol-O-hexoside (**110**). **Part II. In green,** flavonoids identified in Algerian *Thymus algeriensis*. (**a**) Flavone and flavonol compounds: isorhamnetin pentosyl glucuronide (**111**), xanthomicrol (**112**), salvigenin (**113**), genkwanin (**114**), apigenin (**115**), luteolin feruloyl glucuronide (**116**), luteolin pentoside (**117**), luteolin pentosyl-glucoside (**118**), luteolin-7-O-rhamnoside (**119**), 5-desmethylsinensetin (**120**), baicalin (**121**), diosmin (**122**), santin (**123**), europetin (**124**), kaempferol-O-glucuronide (**125**), quercetin-3-β-D-glucoside (**126**), quercetin-O-malonyhexoside (**127**), quercetin-3-O-rutinoside (**128**), myricetin (**129**). (**b**) Flavanol, flavanonol, flavanone, and flavanone glucoside: gallocatechin (**130**), taxifolin (**131**), hesperidin (**132**), neohesperidin (**133**), naringenin-O-hexoside (**134**).

On the other hand, consumer demand for fresh herbs and natural products continuously increases, and plants from the *Thymus* genus have been considered relevant in this context [167,168]. Many species have been the subject of experimental studies confirming the effectiveness of some of their traditional applications [75]. In fact, they have well-established protective health benefits, mostly related to their highly complex phytochemistry [167,169,170,171]. Several groups and subgroups coexist within this genus, each with its own phytochemical characteristics and peculiarities. Some produce mainly lipids, volatile terpenoids (present in the essential oil), and those that are known to have principally non-volatile metabolites “phenolic compounds” in the polar fraction and are low in an essential oil [172]. Species of the genus *Thymus* are considered a high source of the latter compounds. They are characterized by many structures and functions but generally possess an aromatic ring bearing one or more hydroxyl substituents [173]. They are produced via the shikimic acid pathway and are usually involved in plant adaptation to environmental stress conditions [174]. There are different ways to classify them as they exist in many heterogeneous structures, ranging from single molecules to highly polymerized compounds. The secondary metabolite pattern of *T. algeriensis* comprises phenolic acids and flavonoids. The Figure 3, Figure 4, Figure 5 and Figure 6 show the chemical structures of these compounds identified in the different Algerian, Tunisian, and Moroccan species.

There is a wealth of published data for identifying and quantitatively determining phenolic constituents in *Thymus* plant extracts [175,176,177,178,179,180,181,182]. They have been widely investigated for their biological activities in physiological systems, e.g., antioxidant, anti-aging, anticarcinogenic, pro-apoptosis, anti-inflammation, and anti-atherosclerosis. In addition, they are effective in cardiovascular protection, improving endothelial function, and inhibiting angiogenesis and cell proliferation [75,127,128,171]. Furthermore, the content of these compounds in *T. algeriensis* plant extracts (Table 2) depends mainly on the chemotype and origin of the plant raw material, as well as the choice of solvent and extraction procedures [48,61,183,184]. Irrigation conditions, harvest time, storage conditions, and drying treatments are also factors that can affect the final phenolic composition of extracts and their respective biological activities [185]. Thus, many analytical procedures have been developed to quantify phenolic compounds in herbs [186,187].

- Phenolic acids

A wide range of phenolic acids (benzoic and cinnamic acid series) was identified in *T. algeriensis*. They are distinguished from other phenols by their acidic character [192,193]. These compounds are involved in various functions related to plant physiology, including nutrient uptake, protein synthesis, enzymatic activity, photosynthesis, structural components, and allelopathy [194,195]. Phenolic acids are widely present in plant foods (e.g., fruits, vegetables, and cereals), which comprise a significant proportion of the human diet. They exist in association with other structural (cellulose, protein, lignin), polyphenolic or terpene plant components, and smaller organic molecules (e.g., glucose, quinic, maleic, or tartaric acids). They thus constitute the aglycone part of the compound [194,196].

Phenolic acids are, after flavonoids, the most investigated secondary plant metabolites due to their extensive presence in the diet and rapid metabolism in the human body [197]. Beyond their known protective antioxidant behavior attributed to several phenolic hydroxyl groups in their chemical structure, other biological activities of phenolic acids have been reported [197,198,199]. Therefore, they have received much attention in pharmaceutical and medicinal research as they seem to play a role in preventing several human diseases [200].

Depending on the constituent carbon frameworks, phenolic acids can be divided into the following two categories: benzoic acid derivatives (i.e., hydroxybenzoic acids) and cinnamic acid derivatives (i.e., hydroxycinnamic acids) [201]. They are characterized by a 6 and 9 carbon skeleton containing a carboxyl group attached to the benzene ring with one or more hydroxyl or methoxyl groups attached. The cinnamic acids, in addition, have an unsaturated propionic acid side chain attached to the benzene ring [202]. A summary of phenolic acids and derivatives identified in the different Algerian, Tunisian, and Moroccan species of *T. algeriensis* is presented in Table 3. In addition, their structure (from 59 to 90) is identified in each country and that in common is described in Figure 4 and Figure 5.

Most hydroxybenzoic acids include a C6-C1 backbone directly obtained from benzoic acid [199]. In the Algerian *T. algeriensis* (Figure 4), we found the presence of *p*-hydroxybenzoic acid, 2,5-dihydroxybenzoic acid, 3,4-dihydroxybenzoic acid, and 2,3-dimethoxybenzoic acid, which were not detected in the Tunisian species [58,132,184,203]. There are also anisic, ellagic, and salicylic acids [55,132,183,203]. Methyl gallate is only found in Tunisian populations (Figure 4) [48,61].

Furthermore, the content of hydroxybenzoic acids (gallic acid, syringic acid, and vanillic acid) in *T. algeriensis* populations generally varies between Algeria [132,184] and Tunisia [48,57]. However, this difference can also be related to the extraction method. The efficiency of an extraction technique depends on several critical parameters. In fact, the solvent, the nature of the material, the light, the duration of the extraction period, the pH, the temperature, the size of the material, the solvent/substrate ratio, and the liquid-liquid or solid-liquid ratio can influence the chemical composition [204]. In addition to selecting the optimal extraction method, the choice of a suitable technique for detecting and quantifying phenolic compounds is also of high relevance. There is a large amount of published research on this topic, but some analyses still have difficulties [205,206]. Despite this, there is great potential for developing specific methods [207,208].

**Table 3:** Major phytochemicals isolated and characterized from Maghreb *Thymus algeriensis* Boiss. and Reut (Algeria, Morocco, and Tunisia).

* R/P	PP	SA	EX	TA	NC	MC	Ref.
Algeria							
Chelia mountain/Batna	AP	CH *n*-Bu	Mac: 1200 g in MEOH–H2O (80:20) followed by LLEx (CHCl3; *n*-BuOH)	ESI-MS; NMR	10	CH and *n*-Bu: Salvigenin; Cirsimaritin; Santin; Apigenin; Vanillic acid; *p*-hydroxybenzoic acid; Gallic acid; Rosmarinic acid; Oleanolic acid; β-sitosterol.	[58]
Bellezma National Park/Batna	S, L	MEH	Mac: 2.5 g/25 mL MEOH–H2O (80:20) RT	HPLC/UV	15	3-hydroxy-4-methoxycinnamic acid (1.5%); Ferulic acid (0.1%); Anisic acid (26.4%); Salicylic acid (0.2%); Syringic acid (1%); *Trans*-2,3-dimethoxycinnamic acid (0.9%); *Trans*-cinnamic acid (5.4%); Vanillic acid (0.2%); Catechin (0.5%); Epicatechin (0.1%); Europetin (6.1%); Kaempferol (1.5%); Myricetin (0.2%); Quercetin (17.3%); Rutin (0.2%)	[55]
Taglait/Bordj Bou Arreridj	AP	MEH	(1) 1st Mac: 100 g Pow in 1 L MEOH-H2O (85:15, *v*/*v*) for 24 h and 2nd Mac: MEOH-H2O (50:50, *v*/*v*) for 24 h (2) Purification: the extract was suspended in water/acetic acid (97.5:2.5, *v*/*v*) at a ratio of 1:5 (*w*/*v*) and centrifuged at 20,000 × *g*, followed by solid-phase extraction	UHPLC-DAD-ESI-MSn	23	Apigenin di-*C*-hexoside; Apigenin di-*O*-hexuronide; Apigenin-*O*-hexuronide; Caffeoyl rosmarinic acid (isomer 1); Caffeoyl rosmarinic acid (isomer 2); Eriodictyol-*O*-hexoside (isomer 1); Eriodictyol-*O*-hexoside (isomer 2); Kaempferol-*O*-hexuronide (isomer 2); Kaempferol-*O*-hexuronide (isomer 1); Luteolin di-*O*-glucuronide; Luteolin-*O*-hexoside; Luteolin-*O*-hexuronide; Naringenin-*O*-hexoside; Quercetin-*O*-hexoside; Rosmarinic acid; Rosmarinic acid hexoside; Sagerinic acid; Salvianolic acid B; Salvianolic acid E isomer; Salvianolic acid K isomer; Yunnaneic acid E.	[188]
Ain Demin/Ain Defla	L	MEH	Mac: 250 g/3 L MEOH–H2O (80:20) RT	HPLC-PDA-ESI-MS/MS.	35	12-Hydroxyjasmonic acid 12-*O*-hexoside; Apigenin 6,8-di-*C*-hexosides; Caffeic acid glucoside; Caffeoyl ethylrosmarinate; Carnosol; Taxifolin; Eriodictyol glucoside; Eriodictyol; Feruloyl ethylrosmarinate; Gallocatechin; Genkwanin; Isorhamnetin pentosyl; glucuronide; Luteolin feruloyl glucuronide; Luteolin feruloyl glucuronide; Luteolin glucoside; Luteolin glucuronide; Luteolin pentoside; Luteolin pentosyl-glucoside; Malic acid; Naringenin; Phloretic acid; Phloretic acid caffeoyl 3-hydroxy-3-methylglutaroyl; Quinic acid; Rosmarinic acid glucoside; Rosmarinic acid; Salvianolic Acid A; Salvianolic acid K; Schizotenuin F; Xanthomicrol.	[209]
Laghouat province	L	ET	Mac: 15 g/100 mL 100% ETOH; Wb 55 °C/6 h	HPLC	15	2,5-dihydroxybenzoic acid (778.76 µg/g); 3,4-dihydroxybenzoic acid (1.42 µg/g); 4-hydroxybenzoic acid (10.03 µg/g); Caffeic acid (33.3 µg/g); Chlorogenic acid (22.68 µg/g); Cinnamic acid (20.51 µg/g); Ellagic (374.58 µg/g); Epicatechin (824.79 µg/g); Ferulic acid (34.30 µg/g); Gallic acid (10.49 µg/g); Naringin (120.67 µg/g); *p*-coumaric acid (83.80 µg/g); Quercetin (2.84 µg/g); Rutin (280.39 µg/g); Vanillic acid (182.67 µg/g).	[132]
n.m	AP	*n*-Bu	n.m	HPLC-TOF/MS	22	4-Hydroxybenzoic acid (326.67 ng/mL); Apigenin (69.96 ng/mL); Baicalin (608.37 ng/mL); Caffeic acid (52.79 ng/mL); Catechin (tr); Chlorogenic acid (71.09 ng/mL); Diosmin (750.94 ng/mL); Fumaric acid (191.39 ng/mL); Gentisic acid (94.91 ng/mL); Hesperidin (627.14); Morin (52.35 ng/mL); Naringin (328.31 ng/mL); Neohesperidin (406.48 ng/mL); Polydatin (tr); Protocatechuic acid (77.80 ng/mL); Quercetin-3-β-*D*-glucoside (30.81 ng/mL); Rutin (11.77 ng/mL); Salicylic acid (96.57 ng/mL); Scutellarin (2725.67 ng/mL); Syringic acid (89.56 ng/mL); Vanillic acid (50.08 ng/mL).	[203]
Tebessa province	AP	INF DEC ETH	INF: 1 g/H2O (1:100 *m*/*v*); 100 °C; 5 mn RT Deco: 1 g/100 mL H2O; Boilling 5 mn Mac: 1 g/30 mL ETOH -H2O (80:20 *v*/*v*); RT; 150 rpm/1 h	LC-DAD-ESI/MS	70	Apigenin-6,8-*C*-dihexoside: INF (20.70 ± 0.10 mg/g); DEC (18.80 ± 0.10 mg/g); ETH (10.0 ± 0.50 mg/g) Apigenin-7-*O*-glucuronide: INF (12.60 ± 0.50 mg/g); DEC (11.60 ± 0.40 mg/g); ETH (5.75 ± 0.03 mg/g) Apigenin-8-*C*-glucoside: INF (7.60 ± 0.20 mg/g); DEC (6.80 ± 0.10 mg/g); ETH (3.99 ± 0.02 mg/g) Erydictiol-*O*-hexoside isomer: INF (tr); DEC (tr); ETH (tr) Kaempferol-*O*-glucuronide: INF (65.0 ± 0.40 mg/g); DEC (62.20 ± 0.90 mg/g); ETH (16.7 ± 0.20 mg/g) Lithospermic acid A isomer I: INF (12.90 ± 0.20 mg/g); DEC (12.10 ± 0.10 mg/g); ETH (4.54 ± 0.02 mg/g) Lithospermic acid A isomer II: INF (15.80 ± 0.20 mg/g); DEC (16.30 ± 0.50 mg/g); ETH (8.00 ± 0.30 mg/g) Luteolin-7-*O*-glucuronide: INF (8.90 ± 0.10 mg/g); DEC (7.80 ± 0.30 mg/g); ETH (3.06 ± 0.04 mg/g) Naringenin-*O*-hexoside: INF (tr); DEC (tr); ETH (tr) Quercetin-3-*O*-glucoside: INF (4.60 ± 0.20 mg/g); DEC (4.30 ± 0.20 mg/g); ETH (1.73 ± 0.01 mg/g) Quercetin-3-*O*-glucuronide: INF (4.59 ± 0.01 mg/g); DEC (4.40 ± 0.10 mg/g); ETH (1.44 ± 0.02 mg/g) Quercetin-*O*-malonyhexoside: INF (3.44 ± 0.02 mg/g); DEC (3.23 ± 0.04 mg/g); ETH (1.20 ± 0.02 mg/g) Rosmarinic acid hexoside: INF (6.60 ± 0.10 mg/g); DEC (7.06 ± 0.05 mg/g); ETH (2.80 ± 0.10 mg/g) Rosmarinic acid: INF (58.20 ± 0.30 mg/g); DEC (54.40 ± 0.90 mg/g); ETH (29.70 ± 0.70 mg/g) Salvianolic acid K: INF (27.20 ± 0.10 mg/g); DEC (28.60 ± 0.40 mg/g) ETH (13.30 ± 0.30 mg/g) Salvianolic acid B: INF (7.70 ± 0.20 mg/g0); DEC (7.10 ± 0.30 mg/g); ETH (n.d)	[183]
M’Sila province	L; F	EAE, CH, *n*-Bu CH fractions (F1-F31) *n*-Bu fractions (F1-F23) SFE/MAE ext	Mac: ETOH–H2O (70:30 *v*/*v*) (15 L) 24 h followed by LLEx SFE, MAE	HPLC-PDA	21	F16 (CH): Catechin (1.12 ± 0.01 µg/g); Vanillic acid (5.17 ± 0.11 µg/g); Rutin (0.57 ± 0.02 µg/g); t-Ferulic acid (0.20 ± 0.01 µg/g); 2;3-Dimethoxybenzoic acid (6.51 ± 0.59 µg/g); Naringenin (8.97 ± 0.74 µg/g); Carvacrol (0.43 ± 0.01 µg/g) F24 (CH): Vanillic acid (0.23 ± 0.01 µg/g); t-Ferulic acid (0.23 ± 0.01 µg/g); Naringin (0.16 ± 0.01 µg/g); Benzoic acid (10.92 ± 1.21 µg/g); Naringenin (0.90 ± 0.03 µg/g) F30 (CH): Epicatechin (6.78 ± 0.12 µg/g) F13 (EAext): Epicatechin (0.55 ± 0.01 µg/g); *p*-Coumaric acid (1.26 ± 0.81 µg/g); Naringin (4.02 ± 0.39 µg/g); Benzoic acid (5.71 ± 0.47 µg/g) F22 (EAE): 4-Hydroxybenzoic acid (16.31 ± 0.91) µg/g; Vanillic acid (0.22 ± 0.01 µg/g); *p*-Coumaric acid (40.62 ± 3.01 µg/g); t-Ferulic acid (1.46 ± 0.13 µg/g); Naringin (0.46 ± 0.01 µg/g); 2;3-Dimethoxybenzoic acid (7.51 ± 0.47 µg/g) F27 (EAE): Catechin (6.23 ± 0.05 µg/g); 4-Hydroxybenzoic acid (3.61 ± 0.30 µg/g); *p*-Coumaric acid (1.63 ± 0.88 µg/g); t-Ferulic acid (0.84 ± 0.01 µg/g); *o*-Coumaric acid (1.03 ± 0.09 µg/g) *n*-Bu: 4-Hydroxybenzoic acid (0.66 ± 0.02 µg/g); Epicatechin (48.03 ± 2.98 µg/g); Syringic acid (1.93 ± 0.11 µg/g); *p*-Coumaric acid (1.70 ± 0.58 µg/g); Rutin (4.52 ± 0.41 µg/g); t-Ferulic acid (0.55 ± 0.01 µg/g); 2;3-Dimethoxybenzoic acid (3.52 ± 0.20 µg/g); *o*-Coumaric acid (9.83 ± 0.87 µg/g); Naringenin (0.47 ± 0.01 µg/g) MAE ext: Gallic acid (37.97 ± 0.25 µg/g); Catechin (359.80 ± 1.98 µg/g); Chlorogenic acid (1745.98 ± 5.65 µg/g); Vanillic acid (23.92 ± 0.66 µg/g); Epicatechin (2462.75 ± 2.00 µg/g); Syringic acid (615.20 ± 4.03 µg/g); 3-Hydroxybenzoic acid (166.73 ± 1.02 µg/g); Isovanillin (40.42 ± 0.78 µg/g); *p*-Coumaric acid (106.99 ± 0.77 µg/g); Rutin (196.89 ± 1.00 µg/g); Sinapinic acid (46.20 ± 0.63 µg/g); t-Ferulic acid (140.64 ± 0.73 µg/g); Naringin (376.60 ± 2.77 µg/g); Benzoic acid (4157.75 ± 4.67 µg/g); *o*-Coumaric acid (341.55 ± 1.17 µg/g); Quercetin (180.72 ± 0.77 µg/g)	[184]
Jijel province	AP	MEH *n*-Bu	85 g in MEOH-H2O (70:30 *v*/*v*); extraction with solvents with increasing polarities (EA; *n*-BuOH)	UV-visible; NMR	3	5-hydroxy-6,7,3′,4′-tetramethoxyflavone (5-desmethylsinensetin); Quercetin-3-*O*-rutinoside; Luteolin-7-*O*-rhamnoside	[88]
**Morocco**							
Oujda province	AP	AQ	Deco: 50 g/1 L water; 15 mn	HPLC	7	Apigenin; Cinnamic acid; Coumaric acid; Luteolin; Quercetin; Rutin; Syringic acid	[59]
Ta1: Imizar- Azilal/high Atlas of Morocco Ta2: Ait AatabAzilal/high Atlas of Morocco	L, F	EA ET PEE	n.m	GC-MS	18 13 18 20 12 24	Ta1 (EA): Carvacrol (72.69%); *p*-Cymene (6.82%); γ-Terpinene (3.24%); Bornyl acetate (2.02%); Thymol (2.02%) Ta2 (EA): *p*-Cymene (7.53%); Thymol (1.06%) Ta1 (EtOHext): Carvacrol (76.03%); Thymol (3.36%); Camphene (1.21%) Ta2 (EtOHext): Carvacrol (69.54%); *trans*-Caryophyllene (1.65%); Carvacrol methyl ether (1.48%); Borneol (1.46%); Thymol (1.39%) Ta1 (PEext): Carvacrol (69.09%); Thymol (2.54%); *trans*-Caryophyllene (1.16%) Ta2 (PEext): Carvacrol (48.76%); Camphene (5.78%); 1-Octen-3-ol (2.54%); *p*-Cymene (2.19%); β-Linalool (2.15%); γ-Terpinene (1.92%); Epoxylinalol (1.59%); Borneol (1.38%); 4-Isopropyl-1M-2cyclohexane-1-ol (1.20%); Thymol (1.08%)	[56]
**Tunisia**							
Orbata Gafsa Mount	AP	AQ	Deco: 250 g/2 L H2O, 4 h	UHPLC-HRMS/MS	18	12-hydroxyjasmonic acid; 12-hydroxyjasmonic acid sulphate; Apigenin diglucuronide; Apigenin glucoside glucuronide; Apigenin-7-*O*-β-glucuronide; Citric acid; Luteolin; Luteolin glucoside derivative; Luteolin glucuronide derivative; Luteolin-7-*O*-β-glucuronide; Quinic acid; Rosmarinic acid; Scutellarin; Succinic acid; Trihydroxyoctadecedienoic acid isomer; Trihydroxyoctadecenoic acid; Vicenin-2	[50]
Orbata Gafsa Mount	AP	ME	Mac: in MEOH 24 h	HPLC	9	Caffeic acid (26.00 ± 14.00 μg/g); Catechin (16.00 ± 5.00 μg/g); Cinnamic acid (0.00 ± 0.00 μg/g); Coumaric acid (124.00 ± 11.00 μg/g); Epicatechin (136.00 ± 11.00 μg/g); Ferulic acid (42.00 ± 6.00 μg/g); Flavone (0.00 ± 0.00 μg/g); Gallic acid (745.00 ± 12.00 μg/g); Quercetin (126.00 ± 16.00 μg/g); Rutin (89.00 ± 3.00 μg/g); Vanillic acid (615.00 ± 4.00 μg/g)	[57]
Korbous (Ta1) Essabahia (Ta2) Dj Mansour (Ta3) Jendouba (Ta4) Dj chahid (Ta5) Makther (Ta6) Kesra (Ta7) Siliana (Ta8) Sers (Ta9) Sousse (Ta10) Toujene (Ta11) Matmata (Ta12)	L	ME	Mac: 1 g/10 mL MEOH, 24 h	UHPLC-DAD-ESI/MSn	18	Apigenin-di-*C*-hexoside; Apigenin-*O*-hexuronide; Caffeoyl rosmarinic acid; Cirsimaritin; Eriodictyol; Eriodictyol-*O*-hexoside; Kaempferol-*O*-hexoside; Kaempferol-*O*-hexuronide; Luteolin-*O*-hexuronide; Monomethyl lithospermate; Naringenin; Rosmarinic acid; Salvianolic acid E; Salvianolic acid K; Scutellarein-*O*-hexoside-hexuronide; Tetramethyl-scutellarein	[62]
						*Phenolic acids* Rosmarinic acid: Ta1 (531.30 ± 0.50 µg/mL); Ta2 (383.80 ± 0.50 µg/mL); Ta3 (410.40 ± 0.70 µg/mL); Ta4 (1157.80 ± 2.70 µg/mL); Ta5 (593.60 ± 2.10 µg/mL); Ta6 (410.90 ± 0.70 µg/mL); Ta7 (756.30 ± 0.70 µg/mL); Ta8 (391.30 ± 0.50 µg/mL); Ta9 (596.40 ± 0.30 µg/mL); Ta10 (1083.20 ± 3.50 µg/mL); Ta11 (957.00 ± 1.00); Ta12 (807.20 ± 3.00 µg/mL) Caffeoyl rosmarinic acid: Ta1 (39.20 ± 0.10 µg/mL); Ta2 (64.90 ± 0.10 µg/mL); Ta3 (78.90 ± 0.10 µg/mL); Ta4 (85.50 ± 0.10 µg/mL); Ta5 (73.90 ± 0.20 µg/mL); Ta6 (45.80 ± 0.00 µg/mL); Ta7 (232.20 ± 0.20 µg/mL); Ta8 (74.30 ± 0.10 µg/mL); Ta9 (101.60 ± 0.11 µg/mL); Ta10 (86.10 ± 0.30 µg/mL); Ta11 (206.60 ± 1.10 µg/mL); Ta12 (183.00 ± 0.50 µg/mL) *Flavanones* Eriodictyol hexoside: Ta2 (6.30 ± 0.10 µg/mL); Ta3 (31.90 ± 0.10 µg/mL); Ta4 (40.00 ± 0.10 µg/mL); Ta5 (39.10 ± 0.10 µg/mL); Ta 6 (3.50 ± 0.20 µg/mL); Ta7(5.70 ± 0.10 µg/mL); Ta8 (28.70 ± 0.10 µg/mL); Ta9 (52.80 ± 0.10 µg/mL); Ta11 (9.10 ± 0.20 µg/mL); Ta12 (6.00 ± 1.10 µg/mL) Eriodictyol: Ta1 (4.10 ± 0.10 µg/mL); Ta2 (16.90 ± 0.10 µg/mL); Ta3 (4.40 ± 0.30 µg/mL); Ta4 (12.40 ± 0.70 µg/mL); Ta5 (4.40 ± 0.80 µg/mL); Ta6 (8.20 ± 0.10 µg/mL); Ta7 (11.40 ± 0.10 µg/mL); Ta8 (1.10 ± 0.10 µg/mL); Ta9 (5.50 ± 0.20 µg/mL); Ta10 (9.90 ± 0.40 µg/mL); Ta11 (14.70 ± 0.10 µg/mL); Ta12 (42.00 ± 0.10 µg/mL) Kaempferol-*O*-hexoside: Ta2 (83.90 ± 0.30 µg/mL); Ta3 (228.10 ± 0.10 µg/mL); Ta4 (326.30 ± 0.20 µg/mL); Ta5 (360.40 ± 0.50 µg/mL); Ta7(118.40 ± 0.20 µg/mL); Ta8 (95.30 ± 0.10 µg/mL); Ta9 (439.60 ± 0.30 µg/mL); Ta10 (10.0 ± 0.20 µg/mL); Ta12 (108.40 ± 0.10 µg/mL) Kaempferol-*O*-hexuronide: Ta1 (256.30 ± 0.30 µg/mL); Ta2 (363.20 ± 1.90 µg/mL); Ta3 (202.90 ± 1.70 µg/mL); Ta4 (552.00 ± 0.60 µg/mL); Ta5 (213.20 ± 12.70 µg/mL); Ta6 (216.50 ± 0.70 µg/mL); Ta7 (526.40 ± 0.50 µg/mL); Ta8 (225.20 ± 0.40 µg/mL); Ta9 (446.60 ± 9.40 µg/mL); Ta10 (297.10 ± 0.30 µg/mL); Ta11 (862.80 ± 1.20 µg/mL); Ta12 (655.70 ± 2.60 µg/mL) *Flavones* Luteolin-*O*-hexuronide: Ta4 (25.30 ± 0.10 µg/mL); Ta5 (20.6 ± 0.10 µg/mL); Ta7 (12.70 ± 0.10 µg/mL); Ta8 (3.00 ± 0.10 µg/mL); Ta9 (27.90 ± 0.10 µg/mL); Ta10 (5.70 ± 0.10 µg/mL) Apigenin-*C*-di-hexoside: Ta1 (18.40 ± 2.70 µg/mL); Ta2 (10.70 ± 0.10 µg/mL); Ta3 (21.70 ± 0.10 µg/mL); Ta4 (54.10 ± 0.10 µg/mL); Ta5 (53.30 ± 0.10 µg/mL); Ta6 (10.40 ± 0.10 µg/mL); Ta7 (55.30 ± 0.30 µg/mL); Ta8 (53.40 ± 0.10 µg/mL); Ta9 (62.60 ± 0.10 µg/mL); Ta10 (37.70 ± 0.20 µg/mL); Ta11 (40.20 ± 0.10 µg/mL); Ta12 (54.20 ± 0.10 µg/mL) Apigenin-*O*-hexuronide: Ta1 (112.80 ± 0.10 µg/mL); Ta2 (6.80 ± 0.10 µg/mL); Ta3 (3.80 ± 0.10 µg/mL); Ta4 (6.10 ± 0.10 µg/mL); Ta5 (3.50 ± 0.10 µg/mL); Ta6 (9.80 ± 0.10 µg/mL); Ta7 (3.80 ± 0.10 µg/mL); Ta8 (6.10 ± 0.10 µg/mL); Ta9 (3.10 ± 0.10 µg/mL); Ta10 (1.40 ± 0.10 µg/mL) *Phenolic terpene* Carvacrol: Ta11 (2221.60 ± 2.50 µg/mL); Ta12 (1374.70 ± 5.00 µg/mL)	
Gafsa (S1) Tamerza (S2) Kairouan (S3)	AP	ME (S1, S2, S3)	Mac: 9 g powdered plant MEOH, 8 h (Soxhlet apparatus)	HPLC	12	S1: Hydroxyphenylacetic acid (914.26 ± 3.42 μg/g); Gallic acid (723.19 ± 4.10 μg/g); Syringic acid (119.31 ± 4.20 μg/g); Ferulic acid (250.18 ± 3.20 μg/g); Vanillic acid (1189.39 ± 973.30 μg/g); Tyrosin (5013.06 ± 934.10 μg/g); Flavone (128.6 ± 0.40 μg/g); Vanillin (1079.26 ± 57.10 μg/g); (+)-Catechin hydrate (18.01 ± 0.22 μg/g); Rutin (609.62 ± 0.60 μg/g) S2: Hydroxyphenylacetic acid (2053.42 ± 532.20 μg/g); Gallic acid (744.72 ± 12.10 μg/g); Syringic acid (148.45 ± 33.30 μg/g); Ferulic acid (41.64 ± 6.20 μg/g); Methyl gallate (229.84 ± 99.20 μg/g); Vanillic acid (614.72 ± 41.20 μg/g); Tyrosin (59.48 ± 3.90 μg/g); Flavone (65.65 ± 9.60 μg/g); Vanillin (126.08 ± 5.80 μg/g); (+)- Catechin hydrate (4.9 ± 0.70 μg/g); Rutin (88.54 ± 2.80 μg/g) S3: Gallic acid (2780.57 ± 492.10 μg/g); Ferulic acid (4657.94 ± 840.10 μg/g); Flavone (5512.01 ± 372.20 μg/g)	[48]

* R/P: region/province; PP: part of plant; P/H: plant stage/harvest time; SA: sample, EX: the extraction method; TA: technic analysis, NC: number of compounds reported; MC: main compounds AP: arial part; AQ: aqueous extract; CHCl3: chloroform; CH: chloroform extract; DEC: decoction; EA: ethyl acetate; EAE: ethyl acetate extract; Eo: essential oil; ESI-MS: electrospray ionization mass spectrometry; ETOH: ethanol; ETH: hydroethanolic extracts; ET: ethanolic extract; F: flowers; GC-MS: gas chromatography–mass spectrometry; H2O: water; HPLC-PDA: high-performance liquid chromatography photodiode array detection; HPLC-PDA-ESI-MS/MS: high-performance liquid chromatography photodiode array-electrospray ionization-mass; HPLC-TOF/MS: high-performance liquid chromatography-time of flight tandem mass spectrometry; INF: infusion; L: leaves; LLEx: extraction with solvents with increasing polarities; LC-DAD-ESI/MS: liquid chromatography-diode array detector-electrospray ionization-mass spectrometry; Mac: maceration; MAE: microwave-assisted extraction; MEOH: methanol; ME: methanolic extract; MEH: hydromethanolic extract; n.d: not determined; n.m: not mentioned; *n*-BuOH: *n*-butanol; *n*-Bu: *n*-butanol extract; NMR: resonance magnetic nuclear; PEE: petroleum ether extract; S: stems; SFE: supercritical fluid extraction; Tr: trace; UHPLC-HRMS/MS: ultra-high-performance liquid chromatography-high-resolution mass spectrometry.

Some of the hydroxybenzoic acids (Figure 4) detected can also be found in derived forms, such as amides and esters or glycosides [210]. They exhibit multiple physiological functions and high pharmacological potential, mainly attributed to the presence of multiple hydroxyl groups in their chemical structure, making them suitable free radical scavengers [197,211].

In contrast to the hydroxybenzoic acids, the group of hydroxycinnamic acids is the most important class of phenolic acids (Figure 5). They have a structure with three carbon side chains (C6–C3) originating from the phenylalanine and tyrosine pathways [199]. Their derivatives serve as precursor molecules for stilbenes, chalcones, flavonoids, lignans, and anthocyanins [212]. In Tunisian and Algerian populations of *T. algeriensis*, the common hydroxycinnamic acids detected and/or quantified are caffeic acid, cinnamic acid, ferulic acid, *p*-coumaric acid, rosmarinic acid, and salvianolic acid k [48,50,55,56,58,59,61,88,132,183,184,188,203,209,213].

Rosmarinic acid is the dominant caffeic acid derivative in the different extracts of *T. algeriensis*, a common compound in the *Thymus* plants (Table 3, Figure 5). It is more abundant in the infusion (58.20 ± 0.30 mg/g), decoction (54.40 ± 0.90 mg/g) and hydroethanolic extract (29.70 ± 0.70 mg/g) prepared from the Algerian species [183] than in the methanolic extracts of the Tunisian species (1157.80−383.80 µg/mL) [62]. According to Jaouadi et al. [62], high levels of rosmarinic acid are detected only in some Tunisian populations, notably those characterized by an upper semi-arid (1157 µg/mL extract), lower semi-arid (1083 µg/mL extract) and upper arid bioclimate (957 and 807.2 µg/mL). Rosmarinic hexoside acid (2.80−7.06 mg/g) is also found in some Algerian populations and rosmarinic caffeoyl acid (39.20–232.20 µg/mL) in Tunisian ones [61,183,188]. Caffeic acid was quantified at 52.79 ng/mL in the *n*-butanol extract by HPLC-TOF/MS [132] and at 33.3 µg/g by HPLC in the ethanolic extract of the Algerian species [203]. However, it was detected at concentrations of 26.00 ± 14.00 μg/g by HPLC in the methanolic extract of a Tunisian population [57]. Regarding ferulic acid, it is more dominant in the extracts prepared by microwaves (140.64 ± 0.73 µg/g) [184] than in the other preparations (hydro methanolic, ethanolic, *n*-butanol, and chloroform) [48,55,57,132,184]. The same observation was made for the compounds *p*-coumaric acid (106.99 ± 0.77 µg/g) and *o*-coumaric acid (341.55 ± 1.17 µg/g) [184]. Furthermore, HPLC did not detect cinnamic acid in the methanolic extract of the Tunisian species [48]. On the other hand, it was quantified at 20.51 µg/g in the ethanolic extracts of the Tunisian species by HPLC and 5.4% in the hydromethanolic extracts of the Algerian species by HPLC/UV [132]. Carvacrol, a diterpene phenol (Figure 3), was quantified between 48.76% and 76.03% by GC-MS in ethyl acetate and petroleum ether extracts of some Moroccan populations of *T. algeriensis* [56]. As for the Tunisian species, from 1374.70 ± 5.00 to 2221.60 ± 2.50 µg/mL were detected in the methanolic extracts by UHPLC-DAD-ESI/MSn [62]. On the other hand, HPLC-PDAs chloroform extract of the Algerian species revealed only 0.43 ± 0.01 µg/g of carvacrol [184].

Other phenolic acids in Algerian plants are 3-hydroxy-4-methoxycinnamic acid, *trans*-2,3-dimethoxycinnamic acid, *trans*-cinnamic acid, chlorogenic acid, feruloyl ethyl rosmarinate, quinic acid, yunnaneic acid E, lithospermic acid A (isomer I and II), monomethyl lithospermate, and salvianolic acid A [55,132,184,188,203,209]. Many of these substances are well investigated and have demonstrated multiple physiological functions, such as antioxidant, anti-inflammatory, and antimicrobial functions [197,198,214,215,216]. They play an important role in preventing and treating obesity, diabetes, and related disorders [217]. In addition, several studies have shown a relationship between their consumption and the risk of developing certain cancers [218,219].

- Flavonoids

Flavonoids are a large group of hydroxylated polyphenolics with a benzo-γ-pyrone structure found throughout plants. Their synthesis pathway is part of the broader phenylpropanoid pathway, which produces a range of other secondary metabolites, such as lignins, lignans, and stilbenes [220]. All flavonoids share a chemical structure based on the flavan system (C6-C3-C6). Plants synthesize them in response to various abiotic and biotic stresses [221,222]. They function as UV filters, signaling molecules, allelopathic agents, frost and drought protection agents, phytoalexins, detoxifiers, antimicrobials, and anti-herbivore factors [223,224]. Flavonoids are food components with health-promoting properties due to their antioxidant potential through their reducing capacities and/or possible influences on intracellular redox status [225]. These activities depend on their structural diversity. Flavonoid compounds differ structurally by their degree of hydroxylation, the presence of other substitutions and conjugations, and the degree of polymerization [226,227]. About 10,000 flavonoids have been identified, the third-largest group of natural products after alkaloids [228].

Considering the data present in Table 2 and Table 3, *T. algeriensis* appears to be a rich source of these compounds, containing large amounts of flavonoids. These include flavones, flavone glycosides, flavonols, flavanones, flavanone glycosides, flavanols, and flavanonols. The structure of each group of flavonoids (from 92 to 134) identified is represented in Figure 6. Qualitative and quantitative analysis of different extracts of *T. algeriensis* revealed several common chemical structures between the Algerian, Tunisian, and Moroccan populations. It also appears from the data in Table 2 and Table 3 that the Algerian species is more prosperous than the Tunisians [48,50,55,56,57,58,59,61,88,132,183,184,188,203,209]. However, the content of total flavonoids present in *T. algeriensis* can be affected by several factors, such as physiological, genetic, environmental, growth and storage conditions, etc., as well as the choice of solvent and extraction procedures [228,229,230,231,232]. Therefore, properly comparing the different phenolic compositions of Algerian, Moroccan, and Tunisian medicinal plants is difficult, namely, because the plant samples are of different territorial or regional origins. Moreover, the different standard methods for total flavonoid determination (by UV, IR, or NMR spectrophotometry) and the development of chromatographic methods (HPLC-DAD, HPLC-UV, HPLC-MS, LC-MS, LC-DAD, HPLC-PAD, LC-MS-MS, etc.) make it very difficult to carry out the procedure of comparing different results [99,233]. Various identification approaches allow the accumulation of a large amount of data on the composition of medicinal plant by-products. DNA markers are among the methods that can help solve this problem. In addition to morphological, anatomical, and chemical traits, they can differentiate authentic material and are thus an ideal approach for identifying medicinal plant species and populations/varieties of the same species [234].

Flavones are one of the main subgroups of flavonoids and are mainly dominant in the preparations of *T. algeriensis* (Table 3, Figure 6). Their structure has a ketone in the 4-position of the C-ring and a double bond between the 2- and 3-positions. Most of these compounds have a hydroxyl group in position 5 (Figure 6). The hydroxylation of position 7, A-ring or 3′ and 4′, B-ring can vary according to the taxonomic classification of the plants [224]. Cirsimaritin, apigenin, luteolin, and scutellarin were distinguished (Table 3). Apigenin (4′,5,7-trihydroxyflavone) is more concentrated in aqueous extracts (infusions and decoctions) than hydroethanolic or methanolic extracts. It is also more abundant in the Algerian populations than in the Tunisians [61,183,188].

Flavones are widely present in leaves, flowers, and fruits as glycosides [224]. Apigenin-*C*-dihexoside is detected in 20.70 ± 0.10 mg/g, 18.80 ± 0.10 mg/g, and 10.0 ± 0.50 mg/g, respectively, by LC-DAD-ESI/MS in the infusion, decoction, and hydroethanolic preparations of the Algerian species [183]. On the other hand, the Tunisian species showed 62.60–10.40 µg/mL of Apigenin-*C*-di-hexoside and 1.40–112.80 µg/mL of Apigenin-*O*-hexuronide in the methanolic extracts characterized by UHPLC-DAD-ESI/MSn [62]. The following other Apigenin derivatives were detected in *T. algeriensis*: Apigenin diglucuronide and Apigenin glucoside glucuronide in the Tunisian species [50] and Apigenin-8-*C*-glucoside in the Algerian species [183]. In contrast, luteolin and its derivatives (luteolin glucoside, luteolin feruloyl glucuronide, luteolin glucuronid, luteolin pentoside, luteolin pentosyl-glucoside, and luteolin-7-*O*-rhamnoside) have been reported in *T. algeriensis* plants (Algeria, Morocco, and Tunisia) [50,59,61,88,183,209]. The other flavone derivatives, isorhamnetin pentosyl glucuronide, xanthomicrol, salvigenin, genkwanin, and baicalin, are, however, only detected in the extracts of Algerian plants [55,58,203,209].

The aerial parts of *T. algeriensis* are also rich in flavonols and flavonol-glycosides based on kaempferol and quercetin skeletons (Table 3, Figure 6). They are the building blocks of proanthocyanins. In effect, anthocyanins and flavonols are derived from phenylalanine and share common precursors, dihydroflavonols, which are substrates for flavonol synthase and dihydroflavonol 4-reductase.

Flavonols have, compared to flavones, a hydroxyl group in position 3 of the C ring, which can also be glycosylated or methylated [224]. In *T. algeriensis*, methanolic extraction was more efficient in extracting kaempferol-*O*-glucuronide (3,4′,5,7-tetrahydroxyflavone) (862.80–202.90 µg/mL) from the Algerian species compared to the Tunisian ones. Indeed, the content of the flavonol-glycoside in infusion, decoction, and hydroethanolic preparations was (65.0 ± 0.40 mg/g), (62.20 ± 0.90 mg/g), and (16.7 ± 0.20 mg/g), respectively [61,183]. In contrast, quercetin (3,3′,4′,5,7-pentahydroxyflavone) is more abundantly obtained by microwave-assisted extraction (180.72 ± 0.77 µg/g) than by other techniques [55,57,132,184].

In Algerian plants, myricetin (3,5,7,3′,4′,5′-hexahydroxyflavone), diosmin (3′,5,7-trihydroxy-4′-methoxyflavone-7-rhamnoglucoside), and the two *o*-methyl flavonol compounds, europetin (7-*o*-methylmyricetin), and santin (5,7-dihydroxy-3,6,4′-trimethoxyflavone) are also identified [55,58,203].

Flavanones, also known as dihydroflavones, have a saturated C-ring because, unlike flavones, the double bond between the 2- and 3-positions is saturated [224]. The flavanone eriodictyol and its glycosyl form, eriodictyol-*O*-hexoside, have been detected in Tunisian *T. algeriensis*. They are more extensively expressed in the population under the medium semi-arid (4.10–42.00 µg/mL) and high arid (3.50–52.80 µg/mL) bioclimates, respectively [61,209].

The flavanols catechin (359.80 ± 1.98 µg/g) and epicatechin (2462.75 ± 2.00 µg/g) are more concentrated in the microwaved preparations than in the macerated ones [48,55,57,132,184,203]. Naringenin, however, was only detected in trace amounts in the Algerian species’ infusion, decoction, and hydroethanolic preparations. On the contrary, the chloroform extract (F16), contains a large amount (8.97 ± 0.74 µg/g) compared to the other chloroform fraction (F26) (0.90 ± 0.03 µg/g) and the *n*-butanol extract (0.47 ± 0.01 µg/g) [61,183,184]. The flavanol gallocatechin, flavanone glycosides (hesperidin, neohesperidin), and flavanonol Taxifolin were also identified in Algerian *T. algeriensis* [203,209].

Several studies using various in vitro and in vivo methodologies have suggested that flavonoids are the main chemical components responsible for the pharmacological activities of many medicinal plant by-products. Furthermore, it has been determined that flavonoids (60%) and phenolic acids (30%) represent the main phenolic compounds in our diet [235]. In the last decade, flavonoids’ biological, pharmacological, and medicinal properties have been widely reviewed [236,237,238,239]. These molecules have been attributed to positive effects on human and animal health, and the current interest is in disease therapy and chemoprevention [224]. Flavonoids possess antioxidant, vasculoprotective, anti-hepatotoxic, anti-inflammatory, anti-thrombotic, and anti-cholinesterase activities, and many other beneficial properties have also been investigated [224,240].

In addition, flavonoids are effective as anticancer and cardioprotective agents. These activities may be related to the abilities of these compounds to modify the activity of enzyme systems in mammals (kinases, phospholipases, ATPase, lipooxygenases, cyclooxygenases, phosphodiesterases, etc.), with a correlation being observed in some cases between the structure of the flavonoid and its enzymatic activity. Much of these effects can be attributed to the flavonoid’s ability to interact with the nucleotide-binding sites of regulatory enzymes. In addition, flavonoids can bind to Toll-like receptors in the plasma membrane and thus initiate the enzyme induction process [241]. Many protective enzymes have antioxidant functions and can scavenge oxygen and other free radicals [242]. The activity and selectivity of these compounds depend on structural factors (such as oxidation stage, substituents, and the presence of glycosylation), and the search for structural elements defining the beneficial interactions of these compounds has been explored. Using these compounds as molecular models to design selective and potent small molecule inhibitors may be a strategy to overcome the common problem encountered with the therapeutic efficacy of existing drugs [243].

- Other compounds

The organic acid profile (Table 3) revealed seven organic acids (12-hydroxyjasmonic acid 12-*O*-β-*D*-glucoside, schizotenuin F, malic acid, fumaric acid, phloretic acid, phloretic acid caffeoyl 3-hydroxy-3-methylglutaroyl, and quinic acid) in the Algerian *T. algeriensis* [203,209]. Six organic compounds were identified in the Tunisian species (4-hydroxyphenyl acetic acid, tri-hydroxyoctadecedienoic acid isomer, quinic acid, citric acid, 12-hydroxyjasmonic acid, 12-hydroxyjasmonic acid sulphate) [48,50]. Sagerinic acid, in the form of sagerinate, belongs to the cyclobutane lignans class and was also found in Algerian *T. algeriensis*. Furthermore, a phenolic aldehyde (isovanillin), a stilbenoid (polydatin), a pentacyclic triterpenoid (oleanolic acid), and phytosterols (β-sitosterol) have also been identified [57,58,184].

### 4.5. Pharmacological Reports

#### 4.5.1. Antibacterial Effects

The antimicrobial potential is the most investigated therapeutic application of *T. algeriensis* by-products. The antibacterial activity of the essential oils and extracts of the different parts of the plant by the inhibition zone diameter (IZD mm) and/or minimum inhibitory concentration (MIC) and minimum bactericidal concentration (MBC) methods are summarized in Table 4.

Crude extracts and oils isolated from Algerian, Libyan, Moroccan, and Tunisian species possess antibacterial properties, especially against gram-positive bacteria. All species showed broad to moderate inhibition zones according to the well diffusion method results. Fatma et al. [158] investigated the antibacterial activity of essential oils from three different Tunisian regions against *Staphylococcus aureus* with Penicillin as a control for the inhibitory effect. All the essences showed broad zones of inhibition [Eo1 (22.00 mm), Eo2 (63.00 mm), and Eo3 (63.00 mm)] against the bacterium [48]. The same was observed for a Moroccan essential oil, which gave an IZD equal to 51.00 ± 3.40 mm. On the other hand, in the Ben El Hadj Ali et al. [152] study, three different *T. algeriensis* essential oils collected from Tunisian regions revealed moderate activity against the same strain of *Staphylococcus aureus* bacteria ATCC 25923. The IZD of the three oils was 17.20 ± 0.20 mm, 19.40 ± 0.50 mm, and 14.8 ± 0.50 mm [152]. Algerian essential oils also revealed suitable antibacterial activities with IZD values of 18.0 ± 0.70 mm and 17.30 ± 0.58 mm [61].

The microdilution method data indicated that *S. aureus* is more sensitive to oils and extracts of Algerian species. Indeed, in a study by Rezzoug et al. [132], the essential oil extracted from *T. algeriensis* from the Saharan Atlas (Laghouat region) revealed a MIC equal to 32.00 µg/mL, and the ethanolic extract showed a MIC of 65.00 µg/mL. In the study by Messaoudi et al. [189], the ethanolic and methanolic extracts also exhibited high antibacterial potential with MICs of 165.00 µg/mL and 40.00 µg/mL, respectively. Furthermore, in a recent study by Ghorbel et al. [50], the antibacterial screening of the aqueous extract and the essential oil of the Tunisian species revealed a MIC of 83.00 µg/mL and 70.00 µg/mL, respectively.

Petroleum ether, chloroform, and *n*-butanol extracts of the aerial parts of Algerian *T. algeriensis* exhibited high inhibitory activities against *Enterococcus faecalis* ATCC29212, with MICs equal to 12.50 µg/mL, 12.50 µg/mL, and 6.25 µg/mL, respectively. The antibacterial activity of organic extracts may be due to flavonoids, phenolic compounds, and triterpenoids, which are well-known for their antimicrobial properties [58]. In the study of Zouari et al. [49], the essential oil extracted from Tunisian *T. algeriensis* revealed a high antibacterial power with an IZD of 18.50 ± 0.50 mm and a MIC equal to 3.00 μL/mL. However, the MIC and MBC values of the Moroccan essential oil were 0.07% and 0.30%, respectively [155]. In the study by Messaoudi et al. [189], methanolic and ethanolic extracts of Algerian *T. algeriensis* also significantly inhibited the growth of *E. faecalis* ATCC49452 bacterial isolates. The MICs were equal to 80.00 µg/mL and 105.00 µg/mL, respectively [189]. Similarly, the aqueous extract (MICs >83.00 µg/mL) and essential oil (MIC = 140.00 µg/mL) of a Tunisian species exerted moderate inhibitory activity against *E. faecalis* strain ABC3 [50].

In vitro, *T. algeriensis* essential oil exerted significant antimicrobial effects on pathogenic *Bacillus subtilis* bacteria. The highest activity was observed by three Tunisian essential oils (Eo1, Eo2, and Eo3) from different regions of the *B. subtilis* 166 strains [48]. They exhibited antibacterial activity with significant IZD values ranging from 36 to 63 mm and MICs equal to 5.50, 4.00, and 4.50 mg/mL, respectively. Their antibacterial potential was compared to that of the tested antibiotics, chloramphenicol (IZD = 22 mm, MIC = 5 μg/μL) and streptomycin (IZD = 20 mm, MIC = 1 μg/μL). Their antibacterial potential was attributed to monoterpene alcohols such as terpinen-4-ol and linalool. Fatma et al. [57]. showed that a methanolic extract of *T. algeriensis* from another Tunisian region had an IZD equal to 24.00 mm [57]. In addition, the volatile Algerian oil showed an IZD of 42 mm and MIC of 0.50 µL/mL against *B. subtilis* ATCC6633.

The anti-infective activities of volatile oils extracted from the aerial parts of *T. algeriensis* from the north-eastern part of Morocco were evaluated on clinical isolates of *Listeria monocytogenes* 4b (CECT 935) and *L. monocytogenes* EGD-e [158]. The oils significantly inhibited bacteria with IZD of 33.70 ± 0.40 mm and 26.70 ± 2.30 mm, respectively. MICs and MBCs were < 0.5 µL/mL against both strains. According to the results, *T. algeriensis* showed the best bacteriostatic and bactericidal effect, followed by *Eucalyptus globulus* and *Rosmarinus officinalis*, which were also tested in the study. Indeed, its essential oil contained high amounts of borneol, linalool, camphene, or β-caryophyllene, and other components that could exert this antimicrobial effect [158].

Ben El Hadj Ali et al. [152] also evaluated the antibacterial activity of essential oils against *L. monocytogene* with Gentamycin as a positive control. *T. algeriensis* was collected at the vegetative stage from three different localities in the eastern region of Tunisia. The specimens belonged to the following different bioclimates: sub-humid, upper semi-arid, and lower semi-arid. The oils, when tested, had a strong inhibitory effect, with an IZDs between 20 and 45 mm and MICs between 2.00 and 7.50 mg/mL. However, Bukvicki et al. [244] investigation showed remarkable antibacterial activity of Lybian essential oils with MIC = 0.04 ± 0.00 mg/mL and MBC = 0.09 ± 0.02 mg/mL. The oil demonstrated better activity than the positive controls, ampicillin and streptomycin. Phytochemical screening isolated carvacrol and *p*-cymene and it was suggested that they were responsible for the observed antibacterial effects [244].

**Table 4:** Summary of studies on antimicrobial activities carried on Maghreb *Thymus algeriensis* Boiss. and Reut (Algeria, Libya, Morocco, and Tunisia).

SA	TA	Bacteria Strains	Results			Ref.
			IZD (mm)	MIC	MBC/MFC	
Algeria						
L/Eo	DDM	*Escherichia coli* ATCC10536 *Micrococcus luteus* *Staphylococcus aureus* CIP7625 *Candida albicans* IPA200 *C. tropicalis* *C. glabrata* *Saccharomyces cerevisiae* ATCC4226	13.0 ± 0.90 18.0 ± 0.60 18.0 ± 0.70 13.0 ± 0.40 2.04 ± 0.80 18.0 ± 0.60 17.0 ± 0.50	n.m n.m n.m n.m n.m n.m n.m	n.m n.m n.m n.m n.m n.m n.m	[61]
AP/Eo	DDM MBS	*E. coli* SB3 (ESBL) *Enterobacter xiangfangensis* SB2 *Hafnia paralvei* SB1 *Klebsiella pneumoniae* SB4 (ESBL) *K. pneumoniae* SB5 (ESBL) *K. pneumoniae* SB6 (ESBL)	13.54 ± 1.30 13.27 ± 0.12 16.11 ± 1.00 10.26 ± 0.17 12.39 ± 1.46 16.22 ± 2.46	12.50 mg/mL 12.50 mg/mL 6.25 mg/mL 12.50 mg/mL 3.12 mg/mL 1.56 mg/mL	25.00 mg/mL 25.00 mg/mL 25.00 mg/mL 25.00 mg/mL 25.00 mg/mL 12.50 mg/mL	[54]
AP/PEE CH *n*-Bu	DDM	*Enterococcus faecalis* ATCC29212 *E. coli* ATCC25922 *Pseudomonas aeruginosa* DMS1117 *S. aureus* ATCC29213	PEE (12.00 ± 0.30); CHCl3ext (n.i); *n*-Bu (15.0 ± 0.10) PEE (9.50 ± 0.30); CH (8.00 ± 0.30); *n*-Bu (8.00 ± 0.30) n.i n.i	PEE (12.50); CH (12.50); *n*-Bu (6,25) µg/mL PEE (25.00); CH (25.00); *n*-Bu (25.00) µg/mL n.i n.i	n.m n.m n.m n.m	[58]
AP/ME PS	DDM	*Bacillus cereus* ATCC10876 *E. coli* ATCC25922 *M. luteus* NRLL B-4375 *Proteus mirabilis* ATCC35659 *Salmonella typhimurium* ATCC1331*1*	n.m n.m n.m n.m n.m	MEH (n.i); PS (2.34 ± 0.00) mg/mL MEH (n.i); PS (9.37 ± 0.00) mg/mL MEH (n.i); PS (7.03 ± 3.30) mg/mL MEH (37.50 ± 0.00); PS (4.68 ± 0.00) mg/mL MEH (n.i); PS (7.06 ± 3.27) mg/mL	MeH2Oext (n.i); PS (18.75 mg/mL) MeH2Oext (n.i); PS (9.37 mg/mL) MeH2Oext (n.i); PS (9.38 mg/mL) MeH2Oext (>37.5); PS (4.68 mg/mL) MeH2Oext (n.i); PS (9.38 mg/mL)	[188]
L/Eo ET	MDM	*B. subtilis* ATCC11562 *E. coli* ATCC29425 *K. pneumoniae* ATCC43816 *P. aeruginosa* ATCC15442 *S. aureus* ATCC25923 *S. epidermidis* ATCC12228 *C. albicans* ATCC10231 *C. glabrata* ATCC22553	n.m n.m n.m n.m n.m n.m n.m n.m	ET (64.00); Eo (32.00) µg/mL ET (256.00); Eo (64.00) µg/mL ET (256.00); Eo (256.00) µg/mL ET (512.00); Eo (512.00) µg/mL ET (64.00); Eo (32.00) µg/mL ET (128.00); Eo (32.00) µg/mL ET (128.00); Eo (64.00) µg/mL ET (128.00); Eo (32.00) µg/mL	n.m n.m n.m n.m n.m n.m n.m n.m	[132]
AP/ET ME	DDM	*E. cloacae* ATCC49452 *E. faecalis* ATCC49452 *E. coli* ATCC25922 *K. pneumonia* ATCC4352 *P. aeruginosa* ATCC27853 *S. typhimurium* ATCC13311 *S. aureus* ATCC25923	ET (7.00); ME (n.i) ET (12.50); ME (17.00) ET (13.00); ME (10.00) n.i ET (16.50); ME (14.00) ET (9.00); ME (12.00) ET (19.00); ME (15.50)	ET (n.i); ME (160.00) µg/mL ET (105.00); ME (80.00) µg/mL ET (270.00); ME (220.00) µg/mL n.i ET (150.00); ME (185.00) µg/mL ET (130.00); ME (110.00) µg/mL ET (165.00); ME (40.00) µg/mL	n.m n.m n.m n.m n.m n.m n.m	[189]
L, F/Eo1 Eo2	DDM	*Acinetobacter spp* *E. faecalis* ATCC29212 *E. coli* ATCC25922 *P. aeruginosa* ATCC27853 *Salmonella* spp. *S. aureus* ATCC43300	Eo1 (10.12 ± 0.11); Eo2 (12.41 ± 0.08) Eo1 (9.48 ± 0.81); Eo2 (12.24 ± 0.20) Eo1 (15.14 ± 3.25); Eo2 (12.72 ± 0.59) Eo1 (6.00 ± 0.00); Eo2 (6.77 ± 0.25) Eo1 (10.29 ± 0.46); Eo2 (12.31 ± 1.20) Eo1(19.46 ± 3.22); Eo2 (33.28 ± 0.74)	Eo1 (0.05%); Eo2 (0.10%) Eo1 (0.05%); Eo2 (0.10%) Eo1 (0.05%); Eo2 (0.05%) Eo1 (0.80%); Eo2 (0.40%) Eo1 (0.05%); Eo2 (0.05%) Eo1 (0.05%); Eo2 (0.05%)	Eo1 (0.05); Eo2 (0.10) Eo1 (0.05); Eo2 (0.10) Eo1 (0.05); Eo2 (0.05) Eo1 (1.00); Eo2 (0.08) Eo1 (0.05); Eo2 (0.05) Eo1 (0.05); Eo2 (0.10)	[245]
n.m/*n*-Bu	DDM	*E. faecalis* ATCC29212 *E. coli* ATCC25922 *P. aeruginosa* ATCC27853 *S. aureus* ATCC25923	7.00 7.00 6.50 ± 0.70 8.00	n.m n.m n.m n.m	n.m n.m n.m n.m	[203]
AP/INF AP/DEC ETH	MPM	*E. faecalis* *E. coli* *E. coli* ESBL *K. pneumoniae* *K. pneumoniae* ESBL *Listeria monocytogenes* *Morganella morganii* *P. aeruginosa* *S. aureus* MSSA *S. aureus* MRSA	INF (10.00); DEC (10.00); ETH (10.00) INF (5.00); DEC (10.00); ETH (5.00) INF (5.00); DEC (10.00); ETH (5.00) INF (10.00); DEC (10.00); ETH (5.00) INF (10.00); DEC (10.00); ETH (5.00) INF (10.00); DEC (10.00); ETH(10.00) INF (10.00); DEC (10.00); ETH (5.00) INF (20.00); DEC (20.00); ETH (20.00) INF (5.00); DEC (10.00); ETH (2,50) INF (5.00); DEC (10.00); ETH (2,50)	n.m n.m n.m n.m n.m n.m n.m n.m n.m n.m	n.m n.m n.m n.m n.m n.m n.m n.m n.m n.m	[183]
S, L, F, Fr/Eo	DDM	*P. aeruginosa* ATCC27853 *S. aureus* ATCC 25923 *E. coli* ATCC25922	10.00 ± 0.50 17.30 ± 0.58 15.00 ± 0.00	1.66 mg/mL 0.20 mg/mL 2.50 mg/mL	n.m n.m n.m	[139]
AP/Eo1 Eo2 Eo3	DDM	*B. cereus* C1060 *Helicobacter pyllori* J99 *H. pylori 26695* *L. monocytogenes* EGD *Salmonella sp* *S. aureus* CFSA2 *C. albicans*	Eo1 (17.00 ± 1.00), Eo2 (9.00 ± 1.00), Eo3 (n.i) Eo1 (14.33 ± 1.15), Eo2 (13.00 ± 1.00), Eo3 (24.33 ± 0.57) Eo1 (17.00 ± 3.00), Eo2 (15.00 ± 2.00), Eo3 (30.00 ± 0.00) Eo1 (11.66 ± 1.15), Eo2 (n.i), Eo3 (n.i) Eo1 (7.00 ± 0.00), Eo2 (8.33 ± 0.57), Eo3 (n.i) Eo1 (9.33 ± 0.57), Eo2 (n.i), Eo3 (n.i) Eo1 (9.33 ± 0.57), Eo2 (9.00 ± 1.00), Eo3 (9.66 ± 0.57)	n.m n.m n.m n.m n.m n.m n.m	n.m n.m n.m n.m n.m n.m n.m	[135]
n.m/Eo1 Eo2	MDM	*C. albican*	-	Eo1 (11.379 µg/mL) Eo2 (18.037 µg/mL)	n.m n.m	[53]
AP/Eo	DDM	*B. subtilis* ATCC6633 *E. coli* CIP 54.8 *P. aeruginosa* CIPA22 *S. aureus* CIP 7625 *C. albican* *S. cerevisiae* *Fusarium oxysporum F.* ssp*. albedinis.* *Mucor ramanniamus* NRRL6606	42.00 n.i n.i n.i 32.00 46.00 34.00 28.00	0.50 µL/mL 5.00 µL/mL 2.00 µL/mL 2.00 µL/mL 1.00 µL/mL 1.00 µL/mL 1.00 µL/mL 0.50 µL/mL	n.m n.m n.m n.m n.m n.m n.m n.m	[136]
**Libya**						
AP/Eo	MDM	*B. cereus* *E. cloacae* *E. coli* ATCC35210 *L. monocytogenes* NCTC7973 *M. flavus* ATCC10240 *P. aeruginosa* ATCC27853 *S. Typhimurium* ATCC13311 *S. aureus* ATCC6538 *Aspergillus fumigates* *A. versicolor* ATCC11730 *A. ochraceus* ATCC12066 *A. niger* ATCC6275 *Trichoderma viride* IAM5061 *Penicillium funiculosum* ATCC36839 *P. ochrochloron* ATCC9112 *P. aurantiogriseum*	n.m n.m n.m n.m n.m n.m n.m n.m n.m n.m n.m n.m n.m n.m n.m n.m	0.04 ± 0.01 mg/mL 0.05 ± 0.04 mg/mL 0.08 ± 0.03 mg/mL 0.04 ± 0.00 mg/mL 0.03 ± 0.00 mg/mL 0.05 ± 0.00 mg/mL 0.09 ± 0.04 mg/mL 0.08 ± 0.03 mg/mL 0.01 ± 0.00 mg/mL 0.04 ± 0.03 mg/mL 0.01 ± 0.00 mg/mL 0.01 ± 0.00 mg/mL 0.01 ± 0.00 mg/mL 0.01 ± 0.00 mg/mL 0.01 ± 0.02 mg/mL 0.02 ± 0.01 mg/mL	0.08 ± 0.02 mg/mL 0.11 ± 0.07 mg/mL 0.11 ± 0.07 mg/mL 0.09 ± 0.02 mg/mL 0.05 ± 0.00 mg/mL 0.11 ± 0.01 mg/mL 0.18 ± 0.07 mg/mL 0.15 ± 0.05 mg/mL 0.03 ± 0.00 mg/mL 0.03 ± 0.01 mg/mL 0.03 ± 0.00 mg/mL 0.01 ± 0.00 mg/mL 0.01 ± 0.00 mg/mL 0.03 ± 0.02 mg/mL 0.03 ± 0.02 mg/mL 0.04 ± 0.01 mg/mL	[246]
AP/Eo	MDM	*E. feacalis (IBR E001)* *P. aeruginosa (IBR P001),* *Lactobacillus acidophilus (IBR L001)* *S. aureus (ATCC 25923)* *Streptococcus mutans (IBR S001)* *S. pyogenes (IBR S004)* *S. salivarius (IBR S006)* *S. sanguinis (IBR S002)*	n.m n.m n.m n.m n.m n.m n.m n.m	20.00 ± 3.40 µL/mL 80.00 ± 2.25 µL/mL 40.00 ± 0.00 µL/mL 80.00 ± 2,25 µL/mL 40.00 ± 1.15 µL/mL 40.00 ± 0.00 µL/mL 40.00 ± 3.00 µL/mL 40.00 ± 0.00 µL/mL	40.00 ± 6.75 µL/mL 160.00 ± 4.61 µL/mL 80.00 ± 0.00 µL/mL 160.00 ± 4,50 µL/mL 80.00 ± 2.25 µL/mL 80.00 ± 0.00 µL/mL 80.00 ± 4.64 µL/mL 80.00 ± 0.00 µL/mL	[245]
**Morocco**						
S, L/Eo	WDA	*E. faecalis* *E. coli* *K. pneumonia* *S. enterica* *S. aureus* *S. pneumonia* *C. albicans* *C. parapsilosis* *C. glabrata* *Trichophyton violaceum* *T. mentagrophytes* *Microsporum canis*	n.m n.m n.m n.m n.m n.m n.m n.m n.m n.m n.m n.m	0.07% 0.03% 0.07% 0.03% 0.15% 0.07% 0.03% 0.03% 0.07% n.m n.m n.m	0.30% 0.07% 0.30% 0.07% 0.30% 0.30% 0.07% 0.07% 0.30% n.m n.m n.m	[155]
L/Eo	DDM	*E. faecalis* *E. coli* O157H7 *L. monocytogenes* EGD-e *L. monocytogenes* 4b *P. aeruginosa* *S. enteritidis* *S. aureus*	14.70 ± 1.20 17.80 ± 1.70 33.70 ± 0.40 26.70 ± 2.30 15.20 ± 1.00 15.60 ± 2.40 51.00 ± 3.40	< 0.50 µL/mL 1.00 µL/mL < 0.50 µL/mL < 0.50 µL/mL 10.00 µL/mL 1.00 µL/mL < 0.50 µL/mL	< 0.50 µL/mL 2.00 µL/mL < 0.50 µL/mL < 0.50 µL/mL 30.00 µL/mL 1.00 µL/mL < 0.50 µL/mL	[158]
**Tunisia**						
AP/Eo AQ	MDM	*E. cloacae* ABC291 *E. faecalis* ABC3 *E. coli* ABC5 *K. pneumoniae* ABC42 *P. aeruginosa* ABC4 *S. aureus* ABC1 *Acinetobacter baumannii* ABC14	n.m n.m n.m n.m n.m n.m n.m	Eo (>140.00); AQ (>166.00) µg/mL Eo (140.00); AQ (>83.00) µg/mL Eo (140.00); AQ (>166.00) µg/mL Eo (>140.00); AQ (>166.00) µg/mL Eo (>140.00); AQ (>166.00) µg/mL Eo (70.00); AQ (83.00) µg/mL Eo (>140.00); AQ (>166.00) µg/mL	n.m n.m n.m n.m n.m n.m n.m	[50]
AP/ME	n.m	*B. subtilis* *E. coli* *K oxycota* *K. pneumonia* *S. aureus*	24.00 7.00 n.i 10.00 10.00	n.m n.m n.m n.m n.m	n.m n.m n.m n.m n.m	[57]
AP/Eo1 Eo2 Eo3	DDM	*B. subtilis* 166 *E. coli* GM109 *L. monocytogynes* *P. aeruginosa* *S. enteridis* ATCC 502 *S. aureus* ATCC 25923	Eo1 (36.00); Eo2 (63.00); Eo3 (63.00) Eo1 (30.00); Eo2 (50.00); Eo3 (30.00) Eo1 (20.00); Eo2 (45.00); Eo3 (32.00) Eo1 (9.00); Eo2 (74.00); Eo3 (30.00) Eo1 (9.00); Eo2 (43.00); Eo3 (65.00) Eo1 (22.00); Eo2 (63.00); Eo3 (63.00)	Eo1 (5.50); Eo2 (4.00); Eo3 (4.50) mg/mL Eo1 (4.00); Eo2 (1.80); Eo3 (4.00) mg/mL Eo1 (7.50); Eo2 (2.00); Eo3 (4.00) mg/mL Eo1 (22.00); Eo2 (9.00); Eo3 (4.50) mg/mL Eo1 (22.00); Eo2 (2.00); Eo3 (1.50) mg/mL Eo1 (4.50); Eo2 (1.50); Eo3 (1.70) mg/mL	n.m n.m n.m n.m n.m n.m	[48]
R, S, L/Eo1 Eo2 Eo3	DDM	*B. cereus* ATCC11778 *E. coli* ATCC25922 *L. monocytogynes* ATCC7644 *P. aeruginosa* ATCC9027 *S. aureus* ATTCC25923	Eo1 (20.90 ± 0.60); Eo2 (25.50 ± 0.50); Eo3 (18.20 ± 0.30) Eo1 (13.70 ± 0.30), Eo2 (16.00 ± 0.50), Eo3 (12.10 ± 0.40) Eo1 (9.10 ± 0.50), Eo2 (12.50 ± 0.50), Eo3 (8.80 ± 0.30); Eo1 (14.40 ± 0.30), Eo2 (16.80 ± 1.00), Eo3 (13.60 ± 0.50) Eo1 (17.20 ± 0.20), Eo2 (19.40 ± 0.50), Eo3 (14.8 ± 0.50)	Eo1 (2.00), Eo2 (1.00), Eo3 (2.50) µL/mL Eo1 (4.50), Eo2 (3.25), Eo3 (5.00) µL/mL Eo1 (4.50), Eo2 (1.75), Eo3 (4.50) µL/mL Eo1 (3.50), Eo2 (2.25), Eo3 (5.00) µL/mL Eo1 (2.00), Eo2 (1.25), Eo3 (2.50) µL/mL	n.m n.m n.m n.m n.m	[152]
AP/Eo	DDM	*B. cereus* ATCC11778 *E. faecalis* ATCC29212 *E. coli* ATCC25922 *K. pneumoniae* ATCC13883 *P. aeruginosa* ATCC27853 *S. typhimurium* NRRLB4420 *Aspergillus niger* *F. solani*	30.00 ± 2.00 18.50 ± 0.50 14.00 ± 1.00 13.50 ± 0.50 14.50 ± 0.50 15.00 ± 0.50 64.00 ± 3.00 31.00 ± 1.50	1.00 µL/mL 3.00 µL/mL 6.00 µL/mL 6.00 µL/mL 5.00 µL/mL 6.00 µL/mL 2.00 µL/mL 1.00 µL/mL	n.m n.m n.m n.m n.m n.m n.m n.m	[49]

PP/SA: part of plant/sample; P/H: plant stage/harvest time; TA: technic analysis, MBC: minimum bactericidal concentration; MIC: minimum inhibitory concentration; MMC: minimum microbicide concentration; MFC: minimum fungicidal concentration. AP: arial part; AQ: aqueous extract; CH: chloroform extract; DDM: disk diffusion method; DEC: decoction; Eo: essential oil; ESBL: *E. coli* extended producer of β-lactamases; ETH: hydroethanolic extracts; ET: ethanolic extract; F: flowers; Fr: fruits; INF: infusion; L: leaves; MBS: microdilution broth susceptibility; MDM: microdilution method; MPM: microdilution plate method; MRSA: methicillin-resistant *S. aureus*; MSSA: methicillin-sensitive *Staphylococcus aureus*; n.i: no inhibitions; n.m: not mentioned; *n*-Bu: *n*-butanol extract; PEE: petroleum ether extract; PS: purified sample, after the hydromethanolic extract preparation (maceration), the extract was suspended in water/acetic acid (97.5:2.5, *v*/*v*) at a ratio of 1:5 (*w*/*v*) and centrifuged at 20,000× *g*, followed by solid-phase extraction to purify the sample; R: roots; S: stems; WDA: well diffusion assay.

Nikolić et al. [247] reported the effect of *T. algeriensis* essential oil from Libya against *Streptococcus mutans* (IBR S001), *S. pyogenes* (IBR S004), *S. salivarius* (IBR S006), and *S. sanguinis* (IBR S002). The essential oil showed significant antibacterial activities, particularly against S. *mutans*, a known cariogenic species. The MICs and MBCs against all strains were 40 µL/mL and 80 µL/mL, respectively. In addition, *T. algeriensis* oil showed an antibacterial potential equal to that of Streptomycin but superior to that of Ampicillin on *S. sanguinis*. The study showed a correlation between the oil’s antimicrobial activity and chemical composition. It was suggested that the antibacterial activity could be attributed to the presence of the main constituent, thymol, described as an excellent antimicrobial agent in several studies [248]. It could also be related to the involvement of less abundant components in the oil.

The by-products of T. algeriensis, notably the essential oils, were also tested against the gram− bacteria *Acinetobacter* spp., *Enterobacter cloacae*, *E. coli*, *Klebsiella pneumoniae*, *Pseudomonas aeruginosa*, *Salmonella typhimurium* and *S. enterica* (Table 4). The results show that some are less effective against this gram− bacteria than against gram+ bacteria. This can be due to the outer lipopolysaccharides layer or sophisticated efflux pumps or channels that release any substance foreign to the bacteria to the outside. These characteristics would thus influence the penetration capacity of the active compounds in the extracts or oil in gram–bacteria. Nevertheless, some products from T. algeriensis have proven efficacy against gram-negative bacteria. Essential oils of T. algeriensis from Laghouat, Media, Oum El Bouaghi, Souk Ahras, and Biskra regions of Algeria recorded MICs equal to 64.00 µg/mL, 5.00 µL/mL, 0.05%, 2.50 mg/mL, and 12.50 mg/mL, respectively against E. coli. On the other hand, petroleum ether, chloroform, and n-butanol extracts of T. algeriensis from Chelia Mountain, Batna (Algeria), showed a better efficacy, with MICs equal to 25.00 µg/mL [58]. By contrast, ethanolic extracts of the plant collected in Bechar (southwest of Algeria) [189] and Laghouat (Algerian Saharan Atlas) [132] showed MICs equal to 270.00 µg/mL and 256.00 µg/mL, respectively. For the latter, a MIC of 220.00 µg/mL was determined for its methanolic extract [132]. In Ghorbel et al. [50] study, the essential oil and aqueous extract of T. algeriensis from Mount Orbata of Gafsa (Tunisia) had MICs against E. coli equal to 140.00 µg/mL and > 166.00 µg/mL, respectively. Other oils extracted from plants from Korbous, Jdidi Jebel Mountain, and Hammem Sousse have MICs of 4.50 µL/mL, 3.25 µL/mL, and 5.00 µL/mL, respectively [152]. In addition, the oils from the Tunisian plants from Gafsa, Tamerza, and Kairouan originated IZDs of 30.00–50.00 mm and MICs of 1.80–4.00 mg/mL [48]. However, the Libyan plant gave better antibacterial results than the Tunisian plants, with a MIC equal to 0.08 ± 0.03 mg/mL and an MBC of 0.11 ± 0.07 mg/mL [244].

The essential oils and extracts of *T. algeriensis* that showed the best antibacterial activity against *P. aeruginosa* were those from the Tunisian (MIC > 140.00 µg/mL; MIC = 5.00 μL/mL) [49,50] and Algerian regions (MIC = 0.40%; MIC = 2.00 µL/mL) [136,245]. The same is observed for *K. pneumoniae,* responsible for Nosocomial Pneumonia; the extracts and oils that were most effective against it were Algerian (MIC = 256.00 µg/mL), Moroccan (0.07%), and Tunisian (6.00 μL/mL) origin [54,132,155].

#### 4.5.2. Antifungal Effects

Screening tests of the antifungal activity of *T. algeriensis* were performed against seven different opportunistic fungi. They revealed that it had moderate to strong efficacy on these microorganisms (Table 4), and in some studies, these by-products were more effective than the drugs used as a reference in the tests.

In the study by Rezzoug et al. [132], moderate antifungal activity was associated with ethanolic extracts and essential oils of Algerian *T. algeriensis* against *Candida glabrata* (MIC = 128.00 µg/mL and 32.00 µg/mL, respectively) and *C. albicans* (MIC = 128.00 µg/mL and 64.00 µg/mL). In another study by Ouakouak et al. [61], the essential oil showed moderate to strong efficacy on fungi but was better than the reference drug (Itraconazole). It significantly reduced *C. albicans* (13.0 ± 0.40 mm), *C. glabrata* (18.0 ± 0.60 mm), and *Saccharomyces cerevisiae* (17.0 ± 0.50 mm) growth. In the study by Dob et al. [136], the growth of *Mucor ramanniamus* was completely inhibited by *T. algeriensis* oil at 0.5 µL/mL, while the growth of *Fusarium oxysporum,* F. ssp. *albedinis*, *S. cerevisiae,* and *C. albicans* was inhibited at 1 µL/mL. The same MIC was recorded by Tunisian essential oil against *Fusarium solani* [49].

Furthermore, Labiad et al. [155] evaluated the antifungal activity of two Moroccan essential oils against the three pathogenic fungi, *Trichophyton violaceum*, *T. mentagrophytes,* and *Microsporum canis,* and showed potent inhibition of fungal growth in a dose-dependent manner. A significant increase in the inhibition rate depended on increasing the concentration of the different essential oils of *T. algeriensis*. In the preliminary screening of the antimicrobial activity with the well-diffusion method, the essential oils showed significant efficacy in inhibiting the growth of the tested yeasts, with an inhibition percentage higher than 77% [155].

In the study by Bukvicki et al. [244], the antimicrobial activity of a Libyan essential oil was tested against eight fungi (Table 4), aiming to analyze *T. algeriensis* as a potential preservative in soft cheese. For antifungal activity, the oil was active at a content of 0.01–0.04 mg/mL, and a minimum fungicidal concentration of 0.01–0.04 mg/mL was determined. In the in situ treatment of sliced cheese with *T. algeriensis*, the essential oil showed antimicrobial potential against the foodborne pathogenic mold *Penicillium aurantiogriseum* in vitro and in real food systems (cheese). Indeed, the incidence of contamination of the fungus in sliced cheese decreased from 66 to 0.0% with increasing oil content from 20 to 25 µL/mL, respectively. In addition, the sensory evaluation results of the oil-sprayed cheese showed no change in the texture and color of the surface of the cheese slices after 30 days of storage at a temperature of 4 °C. The results indicated color increasing with the addition of oil, while the taste of the cheese decreased but remained above the limits of unappreciated products. With high antimicrobial capabilities, the essential oil of *T. algeriensis* could be recommended as a natural antimicrobial additive to extend the shelf life of soft cheese [244].

#### 4.5.3. Antioxidant Activity

Many different assays are commonly used to assess the antioxidant activities of specific compounds or complex mixtures such as oils or extracts. The results of these assays are generally used to select the most effective samples for further investigation [50].

Several techniques have been used to assess the antioxidant activity of *T. algeriensis* by-products (Table 5), and the DPPH (2,2-diphenyl-1-picrylhydrazyl) radical colorimetric assay is the most widely used. The values usually obtained in the assays are IC_50_ values, indicating the sample concentration required to scavenge 50% of the DPPH radicals. The results show that the essential oil from a plant harvested in Tunisia is the most antioxidant compared to the other products tested (IC_50_ = 0.04 μg/mL) [50]. The aerial parts of this plant were collected during the spring season at the flowering stage in April, in the Mont Orbata (Jebel Orbata) of Gafsa, located west of Sfax, at an altitude of 1165 m. This plant is rich in oxygenated monoterpenes, which can act as radical scavengers [50].

Other by-products have also shown a high antioxidant capacity. The methanolic and *n*-butanol extracts of *T. algeriensis* from two different regions of the Batna province (Aures and Chelia Mountain) possess significant properties with IC_50_s of 1.60 ± 0.13 μg/mL and 5.05 ± 0.12 µg/mL, respectively [58,60]. In addition, Moroccan essential oil (province of Al Hoceima) (IC_50_ = 6.88 ± 0.05 µg/mL) and hydromethanolic extract of *T. algeriensis* from Bordj bou arreridj (Algeria) (IC_50_ = 7.40 ± 0.30 µg/mL) are also effective in scavenging free radicals [155,188]. The remaining by-products cannot be considered inferior because relevant phenolic or terpenoid contents were detected in these studies, which were sufficient to justify antioxidant activity (See Table 1 and Table 2).

Table 5 shows that the methanolic extracts tested by the DPPH method have higher activity than the essential oils, with IC_50_s ranging from 1.60 to 68.80 µg/mL [55,60,150,249,250]. Their potential is because they are polar preparations allowing a better solubility of phenolic compounds with redox properties, enabling them to act as reducing agents, hydrogen donors, and singlet oxygen quenchers [60,247]. Their antioxidant activity depends on the number and position of hydrogen-donating hydroxyl groups bound to aromatic rings. The phenolic compounds can trap free radicals and prevent the oxidation of biological molecules by converting the most numerous ROS into inactive species through donating hydrogen atoms [251].

The other most common method for assessing the antioxidant activity of *T. algeriensis* is the β-carotene bleaching test (BCB). The technique involves the use of an aqueous emulsion of linoleic acid and β-carotene, which is bleached by radicals generated by the spontaneous oxidation of the fatty acid promoted by thermal induction, usually at 50 °C [252,253]. The results are expressed as a percentage of bleaching inhibition (%), which determines the ability of antioxidants to inhibit lipid peroxidation in the initiation and propagation phases. Quantification is based on the rate change at which the absorbance of β-carotene decreases (~470 nm) in the presence of increasing concentrations of the antioxidant being evaluated [252,253].

For *T. algeriensis*, some extracts and oils had a clear capacity to protect β-carotene against bleaching (Table 5). In addition to showing the best anti-free radical capacity (which was even higher than that of the synthetic compound Trolox), the methanolic extracts of the Tunisian populations (Ta11, Ta12) also showed high efficacy in the BCB test [62]. The chemical specificity could explain their efficacy compared to the other by-products tested from *T. algeriensis* in the BCB assay. In more detail, the methanolic extracts analysis of Ta11 and Ta12 leaves (from the upper arid bioclimatic zones) revealed high levels of the phenolic monoterpene carvacrol (2222 and 1375 µg/mL for Ta11 and Ta12 extracts, respectively). These high levels contrast with their absence in the extracts of the other populations studied. Caffeoyl rosmarinic acid was also observed in populations Ta11 and Ta12 at 206.6 and 183 µg/mL of extract, respectively. In addition, the Ta11 population was rich in kaempferol-*O*-hexuronide (862.80 µg/mL extract) [62]. The hydroethanolic extract, infusion, and decoction of aerial parts of *T. algeriensis* from the Biskra province (arid Eastern arias of Algeria) also showed some anti-bleaching β-carotene activity (IC_50_ = 85.00 ± 3.00, 139.00 ± 4.00 and 149.00 ± 3.00 µg/mL, respectively) [183].

The reducing power test (RP) often assesses the electron-donating capacity of antioxidant molecules, i.e., the ability to reduce Fe^3+^ to Fe^2+^. During the reaction, the ferric cyanide complex (Fe^3+^) is reduced to ferrous cyanide (Fe^2+^), changing the solution medium to different shades of green to blue, depending on the reducing power of the test sample. The solution is Perl Prussian blue when a strong reducing compound is detected, which absorbs at 700 nm [254,255].

The reducing power of *T. algeriensis* has also been assessed (Table 5) in eight extracts and one oil. The *n*-butanol extract of the plant from Chelia Mountain, Batna (Algeria), was the best reducing agent, with an IC_50_ of 4.98 ± 0.48 μg/mL [58]. The others were chloroform extract (IC_50_ = 24.5 ± 0.52 μg/mL) and petroleum ether extract (IC_50_ = 25.25 ± 0.08µg/mL). In the study by Ziani et al. [183], decoction (IC_50_ = 49.80 ± 0.40 µg/mL), infusion (IC_50_ = 54.00 ± 0.50 µg/mL) as well as hydroethanolic extract (IC_50_ = 100.20 ± 0.50 µg/mL) of *T. algeriensis* from also showed a reducing capacity. However, Libyan essential oil also showed reduced power [247].

Known as the Trolox equivalent antioxidant capacity (TEAC) assay, the ABTS (2,2′-azino-bis(3-ethylbenzothiazoline-6-sulfonic acid) test is also among the most commonly performed antioxidant studies. ABTS is a stable free radical frequently used to estimate total antioxidant capacity (TAC) [256]. During this test, the stable green-blue radical cationic chromophore, 2,2-azinobis-(3-ethylbenzothiazoline-6-sulfonate) (ABTS^•+^), is produced by oxidation [257]. It results from the reaction of a strong oxidising agent (e.g., potassium permanganate or potassium persulphate) with the ABTS salt. By adding a hydrogen-donating antioxidant to the reaction, there is a reduction of the blue-green ABTS radical and suppression of its characteristic long-wave absorption spectrum (734 nm) [256,258,259].

The scavenging activity of ABTS was measured for about ten extracts and oils obtained from *T. algeriensis* (Table 5). Their capacity to trap the ABTS^•+^ radical was expressed as IC_50_ (µg/mL). The maximum scavenging activity was found in the essential oil (IC_50_ = 6.96 ± 0.02 µg/mL) of a plant harvested in Morocco. In his study by Labiad et al. [155], the ABTS results agree with the DPPH test. They indicate that this plant was more effective in scavenging ABTS^•+^ cationic free radicals than *T. broussonetii* and *T. vulgaris,* also tested, but less effective than the synthetic antioxidant Trolox. The high antioxidant power found in this plant can be attributed to its high thymol (a phenolic compound) content (33.00%).

FRAP (ferric reducing antioxidant power) is another test adapted to quantify the ferric reducing antioxidant power of plant by-products. It is the only assay that directly measures antioxidants (or reductants) in a sample compared to other assays measuring inhibition of free radicals [260]. The reaction consists of reducing Fe^3+^-TPTZ (iron[III]-2,4,6-tripyridyl-*S*-triazine) to Fe^2+^-TPTZ in an acidic medium by the presence of an antioxidant. Tripyridyltriazine (TPTZ) is the most used iron-binding ligand in this assay. However, it can be replaced by ferrozine, for example. In the presence of an antioxidant, the reduction reaction of Fe^3+^-TPTZ results in the creation of a Prussian blue end product, whose color intensity and absorbance at 593 nm will determine the antioxidant power of the test sample [261]. Based on the FRAP assay, the total antioxidant capacity, expressed in mmol Fe^2+^/l of *T. algeriensis* by-products, was very significant. The most remarkable were the Tunisian plant’s methanolic extracts, Ta11 and Ta12 [62]. In fact, the determined values for Ta12 were 20.60 ± 0.20 mmol Fe^2+^/L and for Ta11 were 16.70 ± 0.10 mmol Fe^2+^/L, contrasting with those for the other samples (Ta1 to Ta10), which were below 7.00 mmol Fe^2+^/L. In addition, the hydromethanolic extract had the ability to reduce iron (5.30 ± 0.00 mM FeSO_4_/mg), although to a lesser degree than gallic acid (37.00 ± 0.00 mM FeSO_4_/mg), ascorbic acid (19.00 ± 0.00 mM FeSO_4_/mg), and butylated hydroxyanisole (BHA) (16.00 ± 0.00 mM FeSO_4_/mg), which were used as standard agents [188].

**Table 5:** Antioxidant activities of Maghreb *Thymus algeriensis* (Algeria, Morocco, and Tunisia).

Part of Plant	Product	Antioxidant Assay	Antioxidant Activities	Ref.
Algeria				
n.m	Eo	DPPH BCB	IC_50_ = 3 7.68 ± 0.245 mg/mL IC_50_ = 3 8.86 ± 1.13 mg/mL	[130]
L	Eo	DPPH ABTS	IC_50_ = 8.37 mg/mL IC_50_ = 10.84 mg/mL	[61]
S, L	ME	DPPH ABTS FRAP CUPRAC	IC_50_ = 18.40 ± 0.42 μg/mL IC_50_ = 11.73 ± 0.20 μg/mL A0.5 = 147.44 ± 0.191 μg/mL A0.5 = 25.04 ± 0.86 μg/mL	[55]
AP	PEE CH *n*-Bu	DPPH CUPRAC RP TAC LPAF	PEE (IC_50_ = 69.50 ± 0.68), CHCl3ext (IC_50_ = 79.92 ± 0.30), *n*-Bu (IC_50_ = 5.05 ± 0.12) µg/mL PEE (A0.5 = 22.28 ± 0.24), CH (A0.5 = 27.81 ± 3.06), *n*-Bu (A0.5 = 0.94 ± 0.06) μg/mL PEE (A0.5 = 25.25 ± 0.08), CH (A0.5 = 24.5 ± 0.52), *n*-Bu (A0.5 = 4.98 ± 0.48) μg/mL PEE (15.69 ± 0.001), CH (16.21 ± 0.02), *n*-Bu (20.79 ± 0.19) μg EAA/mg DE PEE (27.80 ± 0.37), CH (24.25 ± 0.45), *n*-Bu (47.43 ± 0.58)%	[58]
n.m	ME	DPPH BCB	IC_50_ = 1.60 ± 0.13 μg/mL AA = 64.31 ± 1.90%	[60]
AP	CH EAE ET AQ	DPPH ABTS	CHT (n.a); EAE (n.a); ET (0.052 ± 0.004 mg/mL); AQ (n.a) CH (n.a); EAE (n.a); ET(42.00 ± 0.99); AQ (152.00 ± 31.00) µg/mL	[262]
L	ME	TAC	IC_50_ = 39.27± 3.47 U/L	[209]
AP	MEH	DPPH ABTS BCB TAC FRAP RP	IC_50_ = 7.40 ± 0.30 µg/mL IC_50_ = 207.00 ± 3.00 µg/mL AA = 90.00 ± 2.00% TAA = 268.00 ± 4.00 µg EAA/mg FRA= 5.3 ± 0.0 mM FeSO4/mg IC_50_ = 512.00 ± 0.00 µg/mL	[188]
L	Eo ET	DPPH ABTS FRAP PM	Eo (IC_50_ = 1.437 ± 4.51 E-05 mg/mL); ET (IC_50_ = 1.56 ± 0.01 mg/mL) Eo (IC_50_ = 0.8960 ± 0.20); ET (IC_50_ = 1.743 ± 0.195 mg/mL) Eo (IC_50_ = 1.39 ± 0.26); ET (IC_50_ = 0.90 ± 0.06) μg/mL) Eo (IC_50_ = 0.43 ± 0.001); ET (IC_50_ = 0.007 ± 0.0006) mg/mL)	[132]
AP	INF DEC ETH	DPPH RP BCB TBARS	INF (IC_50_ = 64.80 ± 0.70); DEC (IC_50_ = 48.00 ± 2.00); ETH (IC_50_ = 131.00 ± 3.00) µg/mL INF (IC_50_ = 54.00 ± 0.50); DEC (IC_50_ = 49.80 ± 0.40); ETH (IC_50_ = 100.20 ± 0.50) µg/mL INF (IC_50_ = 139.00 ± 4.00); DEC (IC_50_ = 149.00 ± 3.00); ETH (IC_50_ = 85.00 ± 3.00) µg/mL INF (IC_50_ = 26.30 ± 0.20); DEC (IC_50_ = 22.70 ± 0.30); ETH (IC_50_ = 40.30 ± 0.30) µg/mL	[183]
S, L, F	EAE *n*-Bu	DPPH	EAE (IC_50_ = 0.30 mg/mL) *n*-Bu (IC_50_ = 1.45 mg/mL)	[190]
AP	Eo	DPPH	IC_50_ = 8379.03 ± 15.00 µg/mL	[138]
AP	HAext	DPPH ABTS TBARS ORAC RP MC HR LIPO SAS	0.235 ± 0.018 mg/mL 0.150 ± 0.002 mg/mL n.a 38.47 ± 39.71 TE/g DW 0.025 ± 0.006 mg/mL n.a n.d 0.083 ± 0.005 mg/mL n.d	[191]
AP	Eo1 Eo2 Eo3	DPPH RP HR TBARS	At 1 mg/mL Eo1 (53.40 ± 0.20); Eo2 (6.30 ± 0.30); Eo3 (7.80 ± 0.20)% n.m Eo1 (IC_50_ = 8.50 ±0.10); Eo2 (IC_50_ = 2.20 ± 0.03); Eo3 (IC_50_ = 3.30 ± 0.08) µg/mL Eo1 (IC_50_ = 106.70 ± 8.40); Eo2 (I_C50_ = n.a); Eo3 (IC_50_ = 911.60 ± 7.40) µg/mL	[135]
**Libya**				
AP	Eo	DPPH	IC_50_ = 0.132 mg/mL	[244]
AP	Eo	DPPH RP BCB TBARS assay	IC_50_ = 1.64 ± 0.05 mg/mL IC_50_ = 0.68 ± 0.01 mg/mL IC_50_ = 1.56 ± 0.12 mg/mL IC_50_ = 0.31 ± 0.01 mg/mL	[245]
**Morocco**				
S, L	Eo	DPPH ABTS	IC_50_ = 6.88 ± 0.05 µg/mL IC_50_ = 6.96 ± 0.02 µg/mL	[155]
S, L, F	Eo	DPPH	IC_50_ = 67.85 ± 1.21 µg/mL	[63]
AP	AQ	DPPH	IC_50_ = 32.40 µg/mL	[156]
L	Eo	DPPH	IC_50_ = 1800 μg/mL	[157]
**Tunisia**				
AP	Eo AQ	DPPH FRAP	AQ (IC_50_ = 0.04 μg/mL); Eo (IC_50_ = 0.06 μg/mL) AQ (IC_50_ = 0.04 μg/mL); Eo (IC_50_ = 0.06 μg/mL)	[50]
L	ME (1–12)	DPPH BCB FRAP	IC_50_ (μg/mL): Ta1 (42.70 ± 2.50); Ta2 (54.50 ± 2.10); Ta3 (52.30 ± 1.40); Ta4 (22.70 ± 0.90); Ta5 (37.80 ± 0.60); Ta6 (40.70 ± 1.00); Ta7 (26.60 ± 1.40); Ta8 (68.80 ± 1.00); Ta9 (32.40 ± 1.00); Ta10 (19.90 ± 1.10); Ta11 (8.90 ± 0.10); Ta12 (10.30 ± 0.40) IC_50_ (mg/mL): Ta1 (1.43 ± 0.00); Ta2 (1.50 ± 0.10); Ta3 (1.81± 0.00); Ta4 (1.04 ± 0.00); Ta5 (1.35 ± 0.30); Ta6 (1.60 ± 0.00); Ta7 (1.13 ± 0.00); Ta8 (1.60 ± 0.00); Ta9 (1.53 ± 0.10); Ta10 (0.40 ± 0.00); Ta11 (0.03 ± 0.00); Ta12 (0.06 ± 0.00) IC_50_ (mmolFe^2+^/L): Ta1 (2.00 ± 0.00); Ta2 (1.20 ± 0.00); Ta3 (0.30 ± 0.01); Ta4 (4.80 ± 0.00); Ta5 (6.80 ± 0.00); Ta6 (1.80 ± 0.00); Ta7 (5.10 ± 0.00); Ta8 (1.00 ± 0.00); Ta9 (4.00 ± 0.00); Ta10 (6.50 ± 0.05); Ta11 (16.70 ± 0.10); Ta12 (20.60 ± 0.20)	[62]
AP	MEt1, ME2, ME3 Eo1, Eo2, Eo3	DPPH ABTS BCB	IC_50_ (%): ME1 (93.00 ± 0.06); ME2 (84.00 ± 0.034); ME3 (81.00 ± 0.26) IC_50_ (%): Eo1 (85.00 ± 0.57); Eo2 (82.00 ± 0.52); Eo3 (83.00 ± 0.10) IC_50_ (%): ME1 (75.00 ± 0.72); ME2 (50.00 ± 0.96); MEt3 (22.00 ± 0.90) IC_50_ (%): Eo1 (16.00 ± 0.12); Eo2 (8.00 ± 0.70); Eo3 (19.00 ± 0.33) IC_50_ (%): ME1 (31 ± 0.91); ME2 (25 ± 0.08); ME3 (50 ± 0.12) IC_50_ (%): Eo1 (10.00 ± 0.52); Eo2 (4.00 ± 0.44); Eo3 (5.00 ± 0.71)	[150]
R, S, L	Eo1, Eo2, Eo3	DPPH	Eo1 (IC_50_ = 9.23 mg/mL); Eo2 (IC_50_ = 4.31 mg/mL); Eo3 (IC_50_ = 6.54 mg/mL)	[152]
AP	Eo	DPPH BCB	IC_50_ = 0.8 mg/mL IC_50_ = 0.5 mg/mL	[49]
AP	Eo	DPPH	Radical scavenging activity values (0.6–5.61%) at 200 µg/ml	[151]

A0.5: absorbance value 0.5; AA: antioxidant activity; ABTS: 2,2′-Azino-bis (3-ethylbenzothiazoline-6-sulphonic acid); AEAC: ascorbic acid equivalent antioxidant capacity; AP: arial part; AQ: aqueous extract; BCB: β-carotene bleaching assay; CHt: chloroform extract; DE: dry extract; DEC: decoction; DPPH: 2,2-diphenyl-1-picrylhydrazil; EAE: ethyl acetate extract; EAA: equivalents of ascorbic acid; ET: ethanolic extract; Eo: essential oil; F: flowers; FRA: ferric reducing ability; HAext: hydro-alcoholic extract; HR: hydroxyl radical scavenging activity; INF: infusion; L: leaves; LIPO: 5-lipoxygenase assay; LPAF: lipid peroxidation activity (ferric thiocyanate assay); MC: metal chelating assay; MEt: methanolic extract; MEHt: hydromethanolic extract; MEt: methanolic extract; n.a: not actif; n.d: not determined; n.m: not mentioned; *n*-Bu: *n*-butanol extract; ORAC: oxygen radical absorbance capacity; PEE: petroleum ether extract; PM: phosphomolybdenum assay; RP: reducing power assay; S: stems; SAS: superoxide anion scavenging activity; TAC: total antioxidant capacity; TBARS: thiobarbituric acid reactive substance; TE: Trolox equivalent.

Furthermore, the total antioxidant capacity, expressed as IC50 (μg/mL), is very high in the Tunisian aqueous extract and essential oil (IC50 = 0.04 and 0.06 μg/mL, respectively) [50], followed by the ethanolic extract (IC50 = 0.90 ± 0.06 μg/mL) of *T. algeriensis* leaves from the Algerian Saharan Atlas (Laghouat region) [132].

The Thiobarbituric acid reactive substances (TBARS) assay is a method of choice for detecting one of the major oxidative stress indicators, lipid peroxidation. This procedure detects thiobarbituric acid reactive substances (TBARS), including lipid hydroperoxides and aldehydes, which increase following oxidative stress. It, therefore, measures the protection of a lipid substrate in the presence of an antioxidant. This assay measures malondialdehyde (MDA), which is the cleavage product of an unsaturated fatty acid endoperoxide resulting from the oxidation of lipid substrates. MDA reacts with thiobarbituric acid (TBA) to form a pink chromogen (TBARS) measured at 532–535 nm. The addition of an antioxidant agent to the test solution inhibits the oxidation process, and the reduced formation of chromogen indicates antioxidant capacity. In this test, an MDA standard is used to construct a standard curve against which unknown samples can be plotted [263].

The results of TBARS analysis on *T. algeriensis* extracts and essential oils are expressed as EC or IC50, the concentration at which there is 50% inhibition of lipid damage (Table 5). The infusion (INF) and decoction (DEC) preparations, as well as hydroethanolic extracts (ETH) of Algerian plants, showed the most promising results compared to the other *T. algeriensis* by-products tested. The IC50 values for INF, DEC, and ETH were 26.30 ± 0.20 µg/mL, 22.70 ± 0.30 µg/mL, and 40.30 ± 0.30 µg/mL, respectively. In addition, Libyan essential oil was also evaluated and exhibited a higher efficiency (IC50 = 0.31 ± 0.01 μg/mL) compared to the positive control, Trolox (IC50 = 3.73 ± 1.90 μg/mL) [245].

The study by Hazzit et al. [135] showed that the three Algerian *T. algeriensis* oils tested have different levels of lipid damage inhibition. In fact, the essential oil from Chréa at 800 m showed a strong antioxidant effect (IC50 = 106.70 ± 8.40 µg/mL), superior to that of the BHT positive control (173.40 ± 4.00 µg/mL). The second oil tested was from Chréa at 1500 m and was not active. The last oil was from El-Asnam and showed only low activity (IC50 = 911.60 ± 7.40 µg/mL) [135]. In this study, *T. algeriensis* showed great chemical variation, even in samples collected in the same locality. This characteristic seems to be common to all essential oils of *Thymus* spp. [264], which could influence biological activities.

Cell culture is widely used to evaluate the antioxidant potential of plant products. In the study by Resq et al. [249], the cytotoxic effect of methanolic extract of *T. algeriensis* (from Algeria) was tested (10–100 µg/mL). After 48 h of incubation, the extract was fully biocompatible with the non-tumoral HaCaT cell model. Following this essay, the antioxidant effect of the extract was also analyzed. The cells were subjected to UVA irradiation (100 J/cm^2^). This caused an increase in the intracellular ROS content, which was subsequently analyzed by the DCFDA (2′,7′-dichlorofluorescein diacetate) test [249]. The analysis showed that incubating the cells with *T. algeriensis* extract before UVA exposure significantly changed ROS levels. Following these experiments, Resq et al. [249] investigated the involvement of the transcription factor Nrf-2 (nuclear factor erythroid 2) in the likely molecular mechanisms involved in the antioxidant effects of *T. algeriensis* methanolic extract. The cells were subjected to the extract treatment for 15–30 min, and the level of the transcription factor Nrf-2 was then analyzed by Western blot. This factor is a crucial regulator of the cellular antioxidant defense system and is highly expressed in epithelial cells, including keratinocytes. This factor is generally held in the cytoplasmic matrix by Kelch-like ECH-associated protein 1 (Keap-1), which subsequently directs it to the proteasome machinery for degradation. In a stressful environment and/or in the presence of antioxidants, the Keap-1 protein is released from Nrf-2. It is translocated into the nucleus and then activates the transcription process of antioxidant genes with sequences called ARE (antioxidant responsive elements) in their promoter region [249]. Following the cells treated with extract and incubation for 15 and 30 min, a significant increase in nuclear levels of Nrf-2 was observed.

#### 4.5.4. Anti-Inflammatory Activity

The study of the anti-inflammatory effects of extracts or essential oils of medicinal plants involves various in vitro procedures [265]. Among these methods, one is the protein denaturation technique. Several inflammatory disorders, such as Parkinson’s disease, Alzheimer’s disease, Huntington’s disease, Amyloid Lateral Sclerosis, Type II diabetes, and cystic fibrosis, are associated with protein aggregation [266,267,268]. Therefore, the inhibition of this phenomenon has been associated with anti-inflammatory activity. The study by Mokhtari et al. [58] evaluated the in vitro anti-inflammatory effect of crude petroleum ether, chloroform, and *n*-butanol extracts of *T. algeriensis* (from Algeria) by the egg albumin denaturation method. According to the results, the extracts and standard drugs prevented the denaturation of the protein (albumin) in a dose-dependent manner. At 800 µg/mL, the chloroform extract showed the highest anti-inflammatory effect with inhibition of 45.27%, followed by the petroleum ether (30.26%) and *n*-butanol (26.03%) extracts. At the same content, the positive control, diclofenac sodium, exhibited the highest inhibition (99.23%) at 800 µg/mL.

Models for in vitro studying anti-inflammatory activity involve techniques in which enzymes and inflammatory mediators are directly exposed to the tested products. In vitro models involving direct monitoring of inflammatory mediator inhibition or activity are probably preferable in terms of sensitivity, reproducibility, and reliability. They allow a better understanding of the mechanisms of action and the inflammatory mediators involved [269]. The in vitro anti-inflammatory potential of *T. algeriensis* methanolic extract (from Morocco) is being evaluated for its effects on the main enzymes involved in the inflammation pathway, namely, the following: cyclooxygenase-1 (COX-1), cyclooxygenase-2 (COX-2), 5-lipoxygenase (5-LOX), and 5-lipoxygenase activating protein (FLAP) [209]. These enzymes, particularly COX-1 and COX-2, are known targets for various non-steroidal anti-inflammatory drugs. Indeed, the arachidonic acid cascade plays a central role in lipid mediators’ biosynthesis with pro- and anti-inflammatory properties [270,271]. They are collectively known as eicosanoids and possess potent biological activities and maintain normal hemostasis, regulating blood pressure, renal function, reproduction, and host defense. When formed in excess under pathological conditions, these molecules elicit pain, fever, and inflammation and play roles in many acute and chronic diseases [271]. The arachidonic acid is released upon phospholipase stimulation and can be converted to the above-referred lipid mediators by cyclooxygenases and lipoxygenases in the subsequent inflammation phase. Cyclooxygenases catalyze the initial step in forming prostaglandins and thromboxane A2 that trigger inflammation. On their hand, lipoxygenases produce hydroperoxyeicosatetraenoic acids, leukotrienes, and lipoxins [272].

In the study of Sobeh et al. [209], it was demonstrated that a *T. algeriensis* extract was more potent against COX-2 (IC_50_ = 0.05 ± 0.01µM) than COX-1 (IC_50_ = 12.4 ± 0.49µM). Furthermore, it was evidenced a potency similar to Diclofenac in inhibiting lipoxygenase in vitro (IC50 = 2.70 ± 0.23 µM) and to the reference 5-LOX inhibitor, zileuton (IC50 = 3.20 ± 0.15 µM). *T. algeriensis* extract showed higher selectivity for COX-2 than for COX-1, with a selectivity index similar to that of the selective COX-2 inhibitor Celecoxib (SI value = 248 and 266.2, respectively) and higher than Indomethacin and Diclofenac [209].

An in silico molecular docking study was also performed to evaluate the binding affinities of some compounds identified in *T. algeriensis* extract with these main enzymes. As a result, it was predicted that compounds have docking positions in the binding sites of the relevant inflammatory proteins similar to those observed for the co-crystallized ligands. The compounds salvianolic acid A, rosmarinic acid glucoside, apigenin 6,8-di-*C*-hexosides, and quercetin pentoside are the best candidates in this in silico study. For 5-LOX and FLAP, the compounds with the highest predicted potential were salvianolic acid A, apigenin 6,8 di-*C*-hexosides, and quercetin pentoside [209]. Salvianolic acid A interacts via hydrogen bonding with Gln413 and via pi interactions with Phe177 and Leu607. It also interacts hydrophobically with Ala410. Apigenin 6,8 di-*C*-hexoside interacts via pi-interactions with Phe177 and His367. It also interacts by a hydrogen bond with Asn554 and indirectly with Ala561 through nearby water molecules. The interactions of both compounds within the active site of 5-LOX suggest their strong potential as high-affinity inhibitors [209]. However, the top scoring compounds for the cyclooxygenases were salvianolic acid A, rosmarinic acid glucoside, and apigenin 6,8 di-*C*-hexoside. Salvianolic acid A binds to COX-1 through hydrogen bonding to Arg120 and hydrophobic interactions with Ser353. The interactions between apigenin 6,8-di-*C*-hexosides and COX-1 involve several amino acids, including hydrogen bonds with Arg120, Ser 516, Ile 517, and Gln192, in addition to the hydrophobic interactions with Phe518 as well as Ile523 [209]. The complex of rosmarinic acid glucoside and COX-2 was stabilized by hydrogen bond interactions with Ser530 and Tyr385; it was further strengthened by ionic exchange of the carboxylate oxygen with Arg513 as well as hydrophobic interactions with Phe518 and Val89 [209]. These candidates represent naturally occurring hits for developing safer anti-inflammatory alternatives to currently used drugs [209].

Researchers use numerous techniques to assess the in vivo anti-inflammatory activities of test plant products [269]. Anti-inflammatory effects of *T. algeriensis* in different doses were evaluated by utilizing different animal models representing various changes associated with inflammation, namely, carrageenan-induced paw oedema, total leukocyte count in paw fluid, and acetic acid-induced vascular permeability. In the study by El Ouahdani et al. [63], the effect was evaluated in vivo in a carrageenan-induced hind paw oedema model. This assay has been increasingly used to test new anti-inflammatory drugs. Indeed, the progression of carrageenan-induced oedema generally correlates with the early exudative stage of inflammation and is a valuable tool for studying systemic anti-inflammatory pathology. The essential oil of *T. algeriensis* (from Morocco) was administered orally, alone (a dose of 150 mg/Kg) or mixed with another oil of *Artemesia herba-alba*, against the positive group, which received Diclofenac (p.o., 1%). All groups received their treatments 1 h before the injection of 1% carrageenan, which was injected under the plantar fascia of the right hind paw. The pharmacological test showed that the essential oil of *T. algeriensis* at a dose of 150 mg/kg inhibited oedema by 83.33 ± 00%. However, when mixed with *A. herba-alba* oil in the same quantity, the oedema inhibition was better (89.99 ± 4.08%). Similarly, for Diclofenac, a maximum inhibition (88.57 ± 0.81%) under the same conditions and at the sixth hour after carrageenan injection [63].

In Sobeh et al. [209] study, the same in vivo model was used. The methanolic extract of *T. algeriensis* was tested at three doses (200, 400, and 600 mg/kg). Diclofenac (20 mg/kg) or dexamethasone (2 mg/kg) were tested as positive controls. The data revealed that rats injected one h earlier with *T. algeriensis* extract (200 and 400 mg/kg, p.o.) showed a slight reduction in oedema thickness compared to control rats. Increasing the dose of the extract to 600 mg/kg did not result in further inhibition of the measured oedema thickness. Diclofenac (20 mg/kg, p.o.) reduced paw thickness by 44% of the control values. Dexamethasone (2 mg/kg, p.o.) showed a 51% reduction compared to vehicle-treated rats [209]. In the study’s second phase, the effect of *T. algeriensis* extract on leukocyte recruitment in the peritoneal cavity of mice was evaluated [209]. In the essay, the mice were treated orally with 200, 400, and 600 mg/kg of *T. algeriensis* extract. Diclofenac (p.o., 20 mg/kg) and dexamethasone (p.o., 2 mg/kg) were used as reference standards. After four hours of intraperitoneal injection of carrageenan solution, the mice treated were euthanized. The peritoneal cavity was washed, and the total number of leukocytes was determined. Before the carrageenan stimulus, mice pre-treated with *T. algeriensis* extract (200, 400, and 600 mg/kg, p.o.) showed a dose-dependent reduction in total leukocyte count. The highest doses (400 and 600 mg/kg) reduced the dose by up to 52 and 62%, respectively, compared to the vehicle. Diclofenac or dexamethasone showed only 39 and 30% reductions in leukocyte counts, respectively. *T. algeriensis* methanolic extract may decrease leukocyte migration into the peritoneal cavity by inhibiting the expression of the chemotactic substance and/or suppressing adhesion molecule production [273]. This anti-inflammatory effect may be related to its main active constituents, rosmarinic acid (and rosmarinic glucoside), a potent anti-inflammatory agent that inhibits NF-κB activation [274].

The effect of a *T. algeriensis* methanolic extract on acetic acid-induced vascular permeability was also studied [209]. This in vivo model allows evaluating the extracts’ activity against the first inflammation phase. Increased vascular permeability is a hallmark of acute inflammation, during which endothelial cells contract and separate at their boundaries to expose the basement membrane, freely permeable to plasma proteins and fluids [273,275]. After an injury, phlogistic agents increase vascular permeability at different times. This can be induced chemically (acetic acid) and causes an immediate reaction lasting 24 h [276]. The inhibition of this phenomenon suggests that *T. algeriensis* extract administered can effectively suppress the exudative phase of acute inflammation. Experimental results showed that injection of acetic acid (0.6%, i.p.) in mice increased vascular permeability, as demonstrated by Evans’ blue absorbance. This effect was attenuated in mice pre-treated one hour before acetic acid injection with *T. algeriensis* extract (400 or 600 mg/kg, p.o.) by 63 and 58%, respectively. The exudates from mice treated with diclofenac (20 mg/kg) or dexamethasone (2 mg/kg) showed Evans blue readings of 73 and 60%, respectively. The anti-inflammatory effect of the extract on the acute inflammation phase could be attributed to the inhibition of inflammatory mediator release and the inhibition of vasodilation [209].

#### 4.5.5. Anti-Pyretic and Anti-Nociceptive Activity

The effect of *T. algeriensis* methanolic extract on brewer’s yeast-induced pyrexia in mice was performed [209]. Moreover, called pathogenic fever, this model is triggered by increased prostaglandin E2 (PGE2) synthesis and release of the endogenous pyrogens IL-1β, IL-6, TNF-α, endothelin-1 (ET-1), corticotropin-releasing factor (CRF), bradykinin, and preformed pyrogenic factor (PFPF). PGE2-dependent fever results mainly from the action of cyclooxygenase enzymes activated in the preoptic area of the hypothalamus [277]. Inhibition of these pyrogens is therefore responsible for the antipyretic effect. In the present study, oral administration of *T. algeriensis* extract (200, 400, and 600 mg/kg, p.o.) did not significantly attenuate the rectal temperature of febrile yeast-induced mice. A trend towards a decrease in body temperature was observed at the highest dose used (600 mg/kg) [209].

The analgesic potential of *T. algeriensis* was evaluated using the abdominal contortion test induced by intraperitoneal injection of acetic acid in mice (the writhing test). This method is based on counting contortions and contractions caused by acetic acid. The model helps to screen for peripherally and centrally acting agents [278]. In addition, it allows the evaluation of anti-nociceptive responses to different types of drugs (i.e., anticholinergics and antihistamines). Acetic acid induction of spasm triggers the activation of various events that determine nociception, such as the release of mediators like histamine, bradykinin, and prostaglandins [279]. Their decrease suggests that the substance administered has an analgesic effect. In an in vivo study, Moroccan *T. algeriensis* essential oil was administered alone at 150 mg/Kg or mixed with the essential oil of *Artemisia herba-alba* to mice, and tramadol was used as a positive control [63]. One hour after *T. algeriensis* essential oil administration, the animals were given acetic acid intraperitoneally. The agitation numbers were recorded afterward. They are manifested by extension of the hind legs, constriction of the abdomen, or rotation of the trunk [209]. The *T. algeriensis* essential oil administered alone showed 52.40 ± 3.10 contractions, a slightly lower analgesic effect than that observed for Tramadol (42.00 ± 2.70) and the mixture with Artemisia (29.80 ± 1.92). This effect is also inferior to that of the methanolic extract of Algerian *T. algeriensis* [209]. At both doses tested (p.o., 200 and 400 mg/kg), the extract showed significant peripheral analgesic activity, represented by a dose-dependent decrease in acetic acid-induced writhes in mice. It showed a 94% reduction in writhing response at the highest dose (400 mg/kg). This effect was greater than that observed with diclofenac (20 mg/kg) or dexamethasone (2 mg/kg).

The hot plate method was used to evaluate the central anti-nociceptive mechanism of *T. algeriensis* methanolic extract in the production of acute analgesia [209]. The pain and hot plate response involves higher brain centers and is a supraspinal organized response [280]. The extract showed a significant analgesic effect, suggesting the presence of centrally acting anti-nociceptive components [209]. Animals pre-treated with the extract (200 and 400 mg/kg, i.p.) showed a longer response latency in a dose-dependent manner. Furthermore, at the high dose of 400 mg/kg, Algerian *T. algeriensis* showed a similar effect to nalbuphine. This centrally active narcotic analgesic was used as the reference standard in this experiment [209].

The analgesic activities of *T. algeriensis* were evaluated using two animal models [62,209]. According to the results, it may have potential clinical applications for treating inflammatory disorders. In the first model, acetic acid indirectly induces the release of endogenous substances that are involved in the modulation of nociception [281]. Resident macrophages and basophils release other factors such as the cytokines IL-1β, TNF, and IL-8 in the abdominal cavity [282,283]. The results of these studies lead to the hypothesis that the anti-nociceptive activity of *T. algeriensis* may result from direct or indirect inhibition of the pro-inflammatory mediator’s release as well as the participation of resident macrophages [62,249]. The hot plate test is the other model used to assess the central anti-nociceptive activity of *T. algeriensis*. The latter significantly increased the reaction time in the trial. The effect can be due to flavonoids in the extract, which are known to cross the blood-brain barrier and affect opioid and other receptor types in the CNS [284,285]. The antinociception exerted by the extract presumably relates to the opioid system [209].

#### 4.5.6. Cytotoxic Activities

Five essential oils and six extracts of *T. algeriensis* (from Algeria, Libya, Tunisia, and Morocco) were tested on 21 normal and cancerous cell models. Interestingly, its by-products were found to have inhibitory activity in vitro on certain cancers.

In the study by Rezzoug et al. [132], the anticancer effects of ethanolic extract and essential oils of *T. algeriensis* (from the Algerian Saharan Atlas, Laghouat region) were evaluated on five human cancer cell lines. These were human breast (MCF-7 and MDA-MB-231), cervical (HeLa), prostatic (PC3), and leukaemia (K562) cancer cell lines. The MTT (3-[4,5-dimethylthiazol-2-yl]-2,5-diphenyltetrazolium bromide) assay was used to investigate the cytotoxic effect of both by-products [132]. The observed antiproliferative activities, presented in the LD50 form, were different between the two. In fact, essential oils (LD50 = 300–746 μg/mL) were much more effective in suppressing cancer cell growth than ethanolic extracts (> 10,000 μg/mL). The essential oil was most effective in inhibiting the growth of K562 cells (LD50 = 300.00 ± 13.00 μg/mL), followed by MCF-7 (647.00 ± 16.00 μg/mL), MDA-MB-231 (715.00 ± 22.00 μg/mL), HeLa (746.00 ± 19.00 μg/mL) and PC3 cells (1067.00 ± 96.00 μg/mL) [132]. On the other hand, the essential oil of the aerial parts of Libyan *T. algeriensis* showed a better effect against MCF7 (62.53 ± 1.88 μg/mL) and HeLa (64.79 ± 1.51 μg/mL) adenocarcinoma cell lines [245]. The same oil was also tested on the growth of the human tumor cell lines NCI-H460 (non-small cell lung cancer), HCT-15 (colon carcinoma), AGS (gastric adenocarcinoma cell line) as well as on normal PLP2 (porcine liver cells) [245]. The determined LC50s were approximately the same (63.94 ± 0.68 μg/mL, 64.13 ± 1.33 μg/mL, and 64.79 ± 1.51, respectively). However, it showed no efficacy against the non-tumor cell line PLP2 at concentrations above 400 μg/mL [245].

In the study by Resq et al. [249], the cytotoxic effect of methanolic extract of *T. algeriensis* (from Algeria) was tested (10–100 µg/mL). Four cell lines were used, two cancerous (A431 and SVT2 cells) and two non-tumoral (HaCaT and BALB/c-3T3 cells). After 48 h of incubation, the extract was fully biocompatible with the two non-tumoral cell lines tested and was slightly toxic to the cancer cells, but only at the highest concentration tested.

In a study by Ouakouak et al. [61], the cytotoxic activity of the essential oil of *T. algeriensis* from the El-Guetfa region, M’sila province (Algeria), was evaluated on the following two human cell lines: HCT116 (colon) and HepG2 (hepatic). Cytotoxicity was evaluated for concentrations between 12.5 and 100 μg/mL of oil using an MTT assay. According to the results, the oil was moderately active against the HepG2 cell line (L50 > 100 μg/mL). On the other hand, it showed cytotoxic activity against HCT116 cells after 48 h of incubation, with LC50 = 39.8 μg/mL and LC90 = 59.6 μg/mL, respectively. At a 100 μg/mL concentration, the essential oil caused 100% inhibition of cells. Moreover, this efficacy is almost equal to that of the reference compound (Doxorubicin). However, in another study by Nikolic et al. [245], Lybian essential oil was tested on the same type of HCT15 cell line but only showed an LC50 equal to 64.13 ± 1.33 μg/mL.

The methanolic extract of the Tunisian *T. algeriensis* can act as a chemopreventive agent, bearing antioxidant properties [57]. In addition, it effectively protects against the in vitro proliferation of U266 cells in a dose-dependent manner. Indeed, it showed a potential to inhibit cell viability by significantly increasing apoptosis in tumor cells. These results suggest that treatment with methanolic extracts rich in phenolic acids may provide an enhanced therapeutic response in human multiple myeloma cells. However, 50 μg/mL did not affect tumor cell proliferation significantly. Furthermore, the induction of apoptosis in tumor cells was associated with the extract’s natural compounds [57].

Another study evaluated the antitumor activity of essential oil of *T. algeriensis* from Morocco against the tumor cell line P815 (mastocytoma) and human peripheral blood mononuclear cells (PBMC). Carvacrol was also tested [56]. The MTT results show that all products have a significant and dose-dependent cytotoxic effect against the P815 cell line. The essential oil was most cytotoxic at 0.0625% (*v*/*v*) with 100% cell lysis, and its LD50 was 0.01%. In addition, at a concentration capable of inducing cytotoxic activity against tumor cells (P815), no antiproliferative effect was observed against normal PBMC for the essential oil. However, on the contrary, a proliferative effect was observed [56]. This phenomenon was lower for carvacrol (115–133% viability after 48 h of treatment), while for the essential oil it was higher (200% viability). Probably, the oil efficiency on tumoral cells is due to the presence of carvacrol in high concentrations (76%). Indeed, the tested pure carvacrol can induce a very high cytotoxic activity, with an LD50 lower than 0.004% *v*/*v* [56]. These results agree with those from other investigations that report that carvacrol has a significant antitumor effect [286].

The cancer chemopreventive effect was studied by Guesmi et al. [153]. HCT116 cells were treated with Tunisian essential oil alone or with TRAIL (TNF-related apoptosis-inducing ligand), which has anticancer effects. The results showed a decrease in cell growth with an increasing oil dose. Its ability to enhance the anticancer effects of TRAIL has also been demonstrated. The essential oil was found to significantly improve the TRAIL-induced growth inhibition of human HCT116 cells in a dose-dependent manner [153].

The mechanisms of these effects were analyzed by different methods, namely, the live dead cell assay, caspase activation, and PARP cleavage [153]. The expression of the death receptor signaling pathway was also analyzed by Western blotting. HCT116 cells were treated with different concentrations of essential oil and analyzed under fluorescence microscopy showed that the volatile fractions induced dose-dependent cell death. Volatile thyme oil dose-dependently increased the number of apoptotic cells from 1.6 to ~ 92.13%. Furthermore, in another essay, the ability of the oil to enhance the apoptotic effect was evaluated. A 0.5 pg/mL concentration of the essential oil-induced moderate cell death. It was observed that 0.5 pg/mL of the oil and 25 ng/mL of TRAIL alone induced reasonable cell death [153]. However, the combination of *T. algeriensis* and TRAIL would significantly increase cell death (81%). The effect of the essential oil on the colony-forming ability of the colon cancer cell line was also performed. This assay would measure the ability of cultured cells to grow and divide into groups, a characteristic present in tumor cells in vivo [153]. According to the results, HCT116 colonies were susceptible to thyme oil and showed a dose-dependent inhibition of colony formation. *T. algeriensis* essential oil, even at 0.5 and 1 μg/mL, showed a ~30% and 100% reduction in colony forming ability, respectively [153]. Furthermore, further in vitro experiments showed that co-treatment of HCT116 cells with either the highest concentration (0.5 or 1 μg/mL) of the essential oil or TRAIL/Apo2L (25 ng/mL) alone significantly increased the activation of caspase 8 (5-fold), caspase 9 (2-fold) and PARP proteolysis (1.5-fold) in a dose and time-dependent manner. In addition, the results showed that oil increases the expression of death receptors (DR) and reduces the expression of TRAIL decoy receptors (DcR). It also up-regulates MAPK pathway signaling molecules (p38 kinase, ERK, and JNK), down-regulates c-FLIP, and over-expresses SP1 and CHOP [153]. The last part of this study was to determine the effect of *T. algeriensis* in the animal model with LPS-induced colon carcinoma [153]. Eight groups of animals (*n* = 6 mice/group) were used in the study. Two main treatment groups of *T. algeriensis* are organized, namely, the (1) group treated with *T. algeriensis* essential oil (oral 12.5 mg/mL) and the other (2) treated with *T. algeriensis* essential oil (oral 50 mg/mL). The others are (3) saline-treated sham group, (4) LPS-treated group (oral 10 µg/mL), (5) 5-FU-treated group (oral 20 mg/kg/day), (6) TS and LPS-treated group (oral 12.5 mg/mL and 10 µg/mL, respectively), (7) TS and LPS (50 mg/mL and 10 µg/mL, respectively), (8) 5-FU and LPS-treated group (20 mg/kg and 10 µg/mL, respectively). The essential oil and the comparator were administered intragastrically daily one hour before LPS treatment for one week. Observations of the mice’s spontaneous behavior and physical parameters were recorded daily before drug administration. The mice were sacrificed at the end of the experiments. The organ tissues (colon, liver, heart, spleen, kidney, and lung) were immediately collected and processed for macroscopic and microscopic analysis. The colon length of the different groups was also determined [153]. After seven days of treatment with a low dose, the mice showed no toxicity and no spontaneous behavioral changes. In contrast, groups of mice treated with LPS alone or in combination showed a loss of body weight and lower consumption of food and water. On the other hand, the mice treated with *T. algeriensis* essential oil gained weight. After macroscopic and microscopic study of the tissues, it was found that the LPS-treated colonies suffered damage and a shortening of length. In contrast, administration of *T. algeriensis* essential oil significantly inhibited colonie shortening. The appearance of the colon in the untreated group treated with *T. algeriensis* essential oil had a typical architecture. In contrast, the colon tissues in the LPS-induced group were significantly marked by inflammation, lesions, and carcinogenesis of tumor buds. Furthermore, it was observed that the lesions were reduced in the group treated with increasing doses of *T. algeriensis* essential oil [153].

#### 4.5.7. Neuroprotective Effect

Acetylcholinesterase (AChE) is a key enzyme of the cholinergic nervous system. It is a serine hydrolase whose primary function is to stop neurotransmission by degrading acetylcholine [287,288]. Other non-neuronal functions are attributed to this enzyme, such as involvement in cell growth, apoptosis, drug resistance pathways, response to stress signals, and inflammation [288]. During the development of Alzheimer’s disease, cholinergic neurons in the forebrain become particularly vulnerable. They are accompanied by a progressive decrease in acetylcholine [289,290]. The main drugs developed for this disease are based on the improvement of this cholinergic function by AChE inhibition. However, their therapeutic effects are moderate and of relatively short duration [291,292]. Despite this and due to the lack of validated alternative mechanisms of action, the vast majority of studies are associated with treatments based on the inhibition of this enzyme. In this context, the use of medicinal plants represents one of the alternative strategies to conventional treatments. In fact, some natural compounds, notably phenolic derivatives, are known to modulate intracellular events involved in various neurodegenerative diseases, including the inhibition of AChE, a central therapeutic target in Alzheimer’s disease [48]. One of the strategies for screening potential inhibitors is to evaluate AChE activity in vitro and measure the associated enzyme kinetics. To our knowledge, only two studies have investigated the ability of *T. algeriensis* by-products to modulate AChE activity.

In the study by Jaouadi et al. (2019), twelve methanolic extracts (from Ta1 to Ta12) of *T. algeriensis* from Tunisia were tested. They showed a moderate ability to inhibit AChE with significant variations between populations. The lowest activity was observed for extracts from population Ta 3 (IC50 = 3.00 mg/mL) from the upper semi-arid bioclimatic zone. The intermediate activity was determined for populations Ta 1, 8, and 10, with IC50 values ranging from 1.00 to 1.20 mg/mL, while populations Ta 11 and Ta 12, from the upper arid zone, showed the highest activity (IC50 of 0.20 and 0.10 mg/mL, respectively). Nevertheless, the inhibitory activity was lower than that observed for donepezil (IC50 = 18.00 ± 0.10 μg/mL), which is a specific acetylcholinesterase inhibitor drug, used as a positive control [62].

Another study on anticholinesterase activity was carried out on methanolic extracts of an Algerian species [55]. The increase in inhibition percentages was remarkably noted with increasing concentrations of the extract. The inhibition rate exceeded 50%, and the IC50 of the extract was deduced to be 154.47 ± 3.55 μg/mL. The methanolic extract of *T. algeriensis* recorded an IC50 of 161.53 ± 22.65 μg/mL against butyrylcholinesterase. Again, the percentages of inhibition increased proportionally to the concentrations. From the concentration value of 25 μg/mL, the difference in the performance of the extract compared to the standard became significantly high [55].

An in vivo research work investigated the effect of *T. algeriensis* on neuropathic pain. According to the International Association for the Study of Pain (IASP), it is caused by injury or disease of the somatosensory nervous system. Primary injury, dysfunction, or transient peripheral or central nervous system disturbance are the leading causes of this pain. Chronic pain can also be caused by systemic diseases such as diabetes, viral infections, multiple sclerosis, and cancer. It is characterized by dysaesthesia (i.e., abnormal unpleasant sensations) and hyperalgesia (an increased response to painful stimuli). Pain in response to a stimulus that does not usually cause pain (allodynia) is also neuropathic pain [293,294]. Several conventional drug treatments for neuropathic pain are available. However, these drugs provide only partial relief in most patients, and specific side effects limit their use [294]. Therefore, further studies are needed to explore and develop new and better therapeutic strategies for treating this type of pain.

The Rezq et al. [249] study examined the possible protective effects of Algerian *T. algeriensis*, and the CCI-induced neuropathic pain model was used. The molecular mechanisms involved at the peripheral (sciatic nerve) and supraspinal (brainstem) levels were studied. In rats exposed to CCI, the mechanical hyperalgesia test (Pinprick test), the acetone drop test (cold allodynia of the paw), as well as the paintbrush test (dynamic mechanical allodynia) were performed. Oral administration of the Algerian *T. algeriensis* methanolic extract (200 mg/kg and 400 mg/kg) significantly altered the neuropathic pain behavior in the CCI model [249].

The effect of the extract on structural changes in the sciatic nerve and brainstem was investigated by performing a histopathological analysis of the tissues in all groups of CCI rats. Based on microscopic observations, structural disturbances of the sciatic nerve and brain stem were reduced after treatments with the methanolic extract. The result was comparable to that observed with pregabalin (positive control) at low doses and better than at high doses [249].

Following the histological analyses, an immunohistochemical study of brain stem tissue was performed. An apoptotic marker, caspase-3, was used to determine the apoptotic neurons in the different samples studied. *T. algeriensis* extract-enhanced synaptophysin expression in the brainstem and suppressed the apoptotic marker caspase-3 in the brainstem. In addition, the effect of *T. algeriensis* (p.o., 200–400 mg/kg) on synaptophysin (SYN) expression was also studied. The utilization of low or high doses of the extract improved sciatic nerve integrity (19.12 ± 0.55% and 16.04 ± 0.44%, respectively). The myelin sheath was also maintained in the low dose (53.22 ± 1.17%) and high dose (62.00 ± 1.67%) CCI rats compared to the normal nerve group (65.55 ± 1.59%) [249].

The effect of the extract on CCI-induced oxidative stress was investigated. The oxidative and nitrosative stress markers iNOS, NADPH oxidase (NOX1), and catalase (CAT) were analyzed. The CCI groups treated with both doses of *T. algeriensis* improved their oxidative status by a decrease in NOX1 levels and a dose-dependent increase in CAT activity compared to the values of the sham rats. Pro-inflammatory enzymes (COX-2 and LOX) and mediators (TNF-α, NF-κB, and PGE 2) were also assessed. They were reduced after administration of the extract to the brainstem and sciatic nerve of rats in the CCI model. Indeed, the extract (between 200 and 400 mg/kg) attenuated the inflammatory response after 14 days of treatment. Rats showed a significant reduction of COX2 (58 and 65%, respectively), LOX (40 and 52%, respectively), and PGE2 (17 and 53%, respectively) compared to the CCI control values. In addition, an increase in TNF-α (a pro-inflammatory cytokine) and NF-κB was observed in both brainstem and sciatic nerve on day 14 post-surgery, indicating a neuroinflammatory response. *T. algeriensis* abolished their increase at all dose levels tested [249].

According to all these results, *T. algeriensis* can effectively protect against painful peripheral neuropathy. The underlying mechanisms could be the suppression of oxidative stress-induced neuroinflammation and apoptosis. The methanolic extract of *T. algeriensis* can be a promising therapeutic option for managing neuropathic pain and related diseases [249].

#### 4.5.8. Effect on the Gastrointestinal Tract

In the study by Beyi et al. [59], the aqueous extract of *T. algeriensis* was tested for its possible spasmolytic and relaxing effects in spontaneously contracting rabbit jejunum preparations. Quantification of relaxation is more accessible in this model than in rat jejunum. Animals were subjected to adrenergic and calcium channel blockade. The cholinergic receptor, the NO, and guanylate cyclase pathways were also tested to assess the extract’s spasmodic and relaxing effects. The results suggest that the activity of the aqueous extract of *T. algeriensis* is probably mediated by Ca^++^ and cholinergic receptor antagonism, and does not involve adrenergic receptors, NO, and guanylate cyclase. The relaxing and antispasmodic effects may be due to apigenin, luteolin, and quercitin, which are present in *T. algeriensis* aqueous extract [295,296]. These components could act alone or with other unidentified compounds [59].

The ulcer protective and antioxidant activity of *T. Algeriensis* essential oil on HCl/ethanol-induced ulcers in rats was also investigated. In addition, the influence of gender on changes in wound healing, gastric acid secretion, and blood flow at the ulcer margin was also studied. Male and female rat groups were treated with essential oil at a concentration of 54, 117, and 180 mL/kg, p.o. HCl/ethanol was administered after one hour to induce ulcers. Macroscopic evaluation of gastric lesions and measurement of mucus production were investigated. In addition, the gastroprotective effect of the essential oil was assessed by evaluating lipid peroxides and reduced glutathione. The activities of the antioxidant enzymes superoxide dismutase (SOD), catalase (CAT), glutathione peroxidase (GPx), and glutathione-*S*-transferase (GST) in the gastric mucosa were also assessed [150].

Oil administration to female rats reduced gastric ulceration induced by acidified ethanol. In fact, treatment with the essential oil (54–180 mg/kg, p.o.) resulted in a dose-dependent reduction of HCl/ethanol-induced gastric lesions, decreasing the ulcer index and the percentage of inhibition mainly at doses of 180 mg/kg for male rats (88.00%) and between 117 and 180 mg/kg for female rats (between 96.25 and 98.85%). In this study, omeprazole could protect the gastric mucosa against HCl/ethanol-induced ulceration (65.95%) [150].

The acidity of the gastric contents in experimental animals pre-treated with the essential oil (female rats) was significantly reduced compared to the ulcer control group. Mucus production of the gastric mucosa was also considerably increased in male rats treated with 180 mg/kg and in female rats (between 117 and 180 mg/kg) compared to the ulcer control group. The results showed protection of the gastric mucosa and inhibition of leukocyte infiltration in the gastric wall in oil-pretreated rats. These results were confirmed by histological observations, which revealed a marked reduction in gastric mucosal damage and cell influx. Some treatment-related histopathological abnormalities were observed in both sexes of rats [150].

The study results indicate that essential oil is protective against HCl/ethanol-induced ulcerogenesis in rats. Females showed higher resistance to ulcers, and gastric lesions occurred less often than in males. The SOD, CAT, GPx, GST, and GSH activities were significantly increased by the administration of the oil to treated rats, suggesting that it can restore these enzymes [150].

#### 4.5.9. Insecticide and Phytotoxic Effects

Plant insecticides have been used to control pests for centuries [297], and they are the main alternative to synthetic insecticides due to their advantages over conventional products [298]. More than 283 plant species belonging to 44 plant families have been involved in habitat manipulation and biological control studies [299]. Fifteen of these plant families have species that have been exploited for their insecticidal properties. The Lamiaceae family is one of the families with the most significant number of species used for habitat manipulation and botanical insecticides [299]. Although plant substances are relatively less effective than modern synthetic substances, their relative safety has opened up a new avenue in plant insecticide research [300].

The insecticidal activity of *T. algeriensis* essential oils has been tested against different insect species to promote them as biopesticides. In the study by Adouane et al. [51], the essential oil of the Algerian species was tested against different development stages of the date moth *Ectomyelois ceratoniae* Zeller (Lepidoptera: Pyralidae). This insect species is known as the carob moth and is the primary pest of dates in Algeria, one of the world’s largest date-producing countries [301]. Its proliferation alters the quality of dates, making them unfit for human consumption and marketing [51]. This study involved the following three assays: contact ovicide toxicity test, fumigant toxicity test (against the adult form), and antifeedant toxicity (against the larval form).

Five doses of 0.5, 1, 1.5, 2, and 2.5 mg/mL of oil were tested on egg hatchlings. The hatching rate of the insects decreased with increasing oil content. The hatching rate was 15% when the eggs were exposed to *T. algeriensis* vapor at 2.5 mg/mL. However, most unhatched eggs had a dead embryo [51]. All applied concentrations of *T. algeriensis* showed a toxic effect on *E. ceratoniae* adults after 3, 6, 12, and 24 h of exposure. This toxic effect increased progressively as the content increased. Insect adults treated with *T. algeriensis* oil reached 80% mortality. In addition, the results of the LC_50_ bioassay also showed an efficacy of the oil of 0.19 mg/mL [51]. In the antifeedant toxicity bioassay, the results showed a significant effect. The ability of the oil to kill L1 first instar larvae was enhanced with increasing doses and exposure time. The essential oil of *T. algeriensis* showed moderate antifeedant functions, resulting in 63.33% efficacy after nine days and 66.67% after 12 days when applied at 10 mg/mL content mixed with the diet [51].

The ovicidal activity of *T. algeriensis* essential oil has been attributed to its monoterpenes. They exert a direct toxic effect on the nervous system of the developing embryo [302]. Essential oils also interfere with the egg hatching process by causing an alteration of oxygen and surface tension within the eggs [303]. Upon contact with adult insects, essential oils alter the physiology and behavior of the insects. They penetrate their respiratory system and cause asphyxiation, leading to final death [304]. In addition, the insecticidal effect of *T. algeriensis* can be due to its aromatic properties that disgust the insects from the food and reduce or stop feeding [305].

In Morocco, the main pests of stored foodstuffs belong to the *Coleoptera* order, and these are *Sitophilus oryzae* (L.), *Rhyzopertha dominica* (Fab.), *Trogoder magranarium* (Everts), and *Tribolium castaneum* (Hbst.). In the study by Labiad et al. [155], the following two of these species were used: *R. dominica* and *S. oryzae*. A volume of 3 μL, 12 μL, and 50 μL, respectively, of essential oil, was tested on adults of both species [155]. According to the results, *T. algeriensis* proved to be potent against both beetle species within the first 24 h for all volumes of oil used (3, 12, and 50 μL) [155]. The main constituents of the tested *T. algeriensis* oil, such as thymol, γ-terpinene, and *p*-cymene, were considered responsible for the insecticidal function [306]. A synergistic effect of thymol and carvacrol on the *Coleoptera* and *Lepidoptera* orders has been reported in some studies [307,308]. They cause disorganization of the cell membrane of the target insects, resulting in a loss of permeability [309]. In addition, other minor constituents of the oil may also participate in this efficacy through critical synergistic effects.

The insecticidal activity of three *T. algeriensis* essential oils from Tunisia was also tested on the larvae of the cotton caterpillar, *Spodoptera littoralis* Boisd. (Lepidoptera: Noctuidae) [152]. It was studied against the third larval stage by a biological fumigation test. The three essential oils of *T. algeriensis* (Eo1, Eo2, and Eo3), at different essential oil concentrations of 0, 25, 50, 100, and 200 μL/L, were highly toxic. The study showed that as the concentration of essential oils increased, the percentage of mortality increased. However, the lowest concentration tested, 25 μL/L of air, gave 0% mortality for Eo1 (Korbous region) and Eo3 (Hammem Sousse region) and 13% mortality for Eo2 (Dj. Jdidi region) [152]. The mortality percentages increased to 100% when the concentration increased to 100 μL/L of air for Eo2 and Eo3. The LC_50_ value ranged from 44.25 μL/L air for Eo3 (with limits of 41.75 and 46.5 μL/L air for the lower and upper limits, respectively) to 112.75 μL/L air for Eo1 (with limits of 86.5 and 131 μL/L air for the lower and upper limits, respectively) [152]. The LC_90_ values were 157.00, 74.25, and 56.25 μL/L of air for Eo1, EO2, and Eo3, respectively. The insecticidal activity of Tunisian *T. algeriensis* oils is probably related to the following main constituents: α-pinene, 1,8-cineole, caryophyllene oxide, camphor, linalool, camphene, and *p*-eugenol, which were associated with toxicity in many investigations [300,310,311]. However, the insecticide activity is inherently variable between oils for several reasons, such as plant age, plant tissues or organs, interactions between structural components, etc. [152].

In the same study [152], phytotoxic activity was also investigated. The inhibitory potential of *T. algeriensis* oils on seed germination, hypocotyl and root length, and dry weight of *Medicago sativa* L. and *Triticum æstivum* L. seedlings was investigated. Different essential oil contents (0.10, 0.25, 0.50, and 1.00 mg/mL) were tested, and their allelopathic activity on the germination of *M. sativa* L. and *T. æstivum* L. seeds was found to be locally variable. The oils significantly inhibited the germination of *M. sativa* seeds. The germination percentage varied between 62.00 and 0.00% on the seventh day of germination. Maximum inhibition of seed germination was 100% at 1 mg/mL. At a low content of 0.10 mg/mL, they were also effective with 20.50, 27.00, and 27.50% for Eo3, Eo2, and Eo1, respectively. Rootlet growth is also inhibited. Inhibition ranged from 36.5 (at 0.10 mg/mL; EO3) to 100% (at 1.00 mg/mL, all oils) and hypocotyl from 28.8 to 100%. Biomass production was slightly inhibited in the presence of different oils at 0.10 mg/mL of oil, and the dry weight of seedlings treated with 1.00 mg/mL of different samples was strongly reduced (100%).

#### 4.5.10. Other Effects

The acaricidal effect of the essential oil of *T. algeriensis* was tested on the *Varroa destructor* [134]. The acaricide treatment used was spraying the oil at different doses (0.1, 0.3, and 0.5%, *v*/*v*). It was applied to the top of the hive frames using sprayers to ensure contact with the treatment with *Varroa* mites in the four batches of hives (A, B, C, and D). The results of the treatments showed that the oils did not have a negative effect on the activity of the bee colony and the egg laying of the queen. On the other hand, applying the oils reduced the infestation of the different batches infected by *V. destructor*. The mortality rates obtained are A (4.1%), B (24.0%), C (32.4%), and D (32.6%). The average number of dead mites was for lot A (173), lot B (435), lot C (1274), and lot D (1366) [134].

The antileishmanial activity of *T. algeriensis* was evaluated in a cytotoxic model involving murine macrophage cells RAW264.7 [151]. Two species of Leishmania were used, *L. major* and *L. infantum*, and the classical thiazolyl blue tetrazolium bromide [3-(4,5-dimethyl thiazol-2-yl2,5-diphenyltetrazolium) bromide] MTT test was applied. *T. algeriensis* essential oil was effective against parasites and non-cytotoxic to cells after 24 h of incubation. It had a leishmanicidal activity against both Leishmania species, with an IC50 value equal to 0.43 µg/mL for L. major and 0.25 µg/mL for *L. infantum*. In addition, the oil showed low cytotoxicity when incubated with macrophages [151].

Inhibition of angiotensin I-converting enzyme (ACE, EC 3.4.15.1) activity is a practical therapeutic approach to combat hypertensive disorders. The activity of *T. algeriensis* against this enzyme was tested as it is traditionally used in Tunisian folk medicine to treat hypertension [49]. The essential oil showed a dose-dependent ACE inhibitory activity (50, 100, 150, or 200 μg/mL). The IC50 value was 150 μg/mL, suggesting that *T. algeriensis* could be used as an ACE inhibitor to prevent and remedy hypertension.

Using the erythrocyte osmotic fragility model, all extracts (petroleum ether, chloroform, and *n*-butanol) of *T. algeriensis* showed an anti-haemolytic effect in a concentration-dependent manner [58]. The highest protective effect was noted for the *n*-butanol extract, with a 50% haemolysis inhibitory concentration value of 322.85 ± 0.87 µg/mL, followed by the chloroform extract at 443.25 ± 0.52 µg/mL. Petroleum ether extract showed the lowest activity with 19.51 ± 0.17% at 800 µg/mL.

#### 4.5.11. Toxicity

Extracts and essential oils of *T. algeriensis* have been studied for various pharmacological activities; however, data on their potential toxicity are limited.

In a 14-day acute toxicity study in adult male and female Wistar rats, the essential oil collected orally (between 300 and 500 mg/kg body weight) showed no toxicity. Anatomical results showed no abnormal organ damage in the rats. Neither female nor male rats showed toxicity or mortality, and there were no abnormal physiological or behavioral changes or alterations in body weight at any time during the 14 days of observation. Histological examination of the liver and kidneys showed no difference from the control group [150].

In another study, acute oral toxicity was evaluated in two-month-old mice. After 14 days of treatment with *T. algeriensis* essential oil (150 mg/kg body weight) or combined with *Artemisia herba-alba*, no abnormal behavior (signs of toxicity) or death of the mice was observed. All the animals survived after the test. Their body weight remained almost stable over time, and there was no significant difference in the body weight variation between treated and control mice over 14 days [63].

## 5. Conclusions

The botanical characteristics, traditional uses, chemical composition, and pharmacological properties of *Thymus algeriensis* Boiss. and Reut have been summarised in this review. It can be seen that this plant is rich in various phenolic and terpenic compounds. So far, all these extracts and oils have antitumor, anti-inflammatory, antioxidant, antibacterial, and other pharmacological activities. It is widely used in popular medicine in Northwest Africa, precisely in the Maghreb, where it is endemic. This region is represented by Algeria, Libya, Morocco, Mauritania, and Tunisia. It is used there traditionally as an astringent, expectorant, stimulant, hypertonic, etc. Most of the reported research was carried out with various extracts of different parts of the plant, and it confirms some of its traditional uses. Nevertheless, this extensive knowledge of this species still has some gaps, and further investigations are needed. First of all, given its particular endemism in the Maghreb region, this species should be explored in more detail. This is particularly the case for the two Moroccan and Libyan species, whose phytochemistry has only been examined by two investigations for Morocco and none for Libya. As for the pharmacological research, both species have been evaluated only for their antimicrobial (six studies in total), anti-inflammatory (one study for the Moroccan, none for the Libyan), antitumor/cytotoxic (two studies), antioxidant (six studies), and insecticide (only one study for the Moroccan species) activities. Only one toxicological report and two investigations into the effect of Moroccan *T. algeriensis* on the intestinal tract (spasmolytic and gastroprotective effects) have been conducted. Therefore, future research should focus on these two so far side-lined species, which in combination with the high degree of endemism, the probability of finding new active molecules is quite high. Secondly, this species has only been extensively assessed for its in vitro antimicrobial activity, whereas a study of its antiviral and antiparasitic activities could provide new interesting bioactive compounds. Thirdly, several traditional claims for *T. algeriensis* remain unexplored. Indeed, while its anti-inflammatory, antipyretic, antinociceptive, and neuroprotective uses have been scientifically investigated, its use against diabetes and cardiovascular disease has not. Therefore, future studies should be conducted to demonstrate the usefulness of this species against these types of diseases. Fourthly, despite the pharmacological activities performed, little has been performed to progress the various co-products of *T. algeriensis* toward clinical trials. Studies on some biological activities have been conducted in vitro/in vivo, and some data have not been more thoroughly analyzed. Thus, more research is needed on the phytochemical, pharmacological, and toxicological properties of the co-products of this species.

In conclusion, *Thymus algeriensis* Boiss. and Reut can be considered as a natural product with potential for future use as medicine, but further investigations are needed. Numerous pharmacological studies have highlighted its therapeutic potential as an antimicrobial, antioxidant, anti-inflammatory, anticancer, antipyretic, antinociceptive, etc., which could therefore serve as a basis for further research. However, future studies should be carried out based on traditional uses. They should be developed on different cell lines and animal models with appropriate controls and doses, for pharmacological and toxicological evaluations, as well as elucidation of the mechanisms of action of the identified bioactive compounds.

## Figures and Tables

**Table 2:** Total phenolic, flavonoid, phenolic acids, flavonol, anthocyanin, and tannin content of Maghreb *Thymus algeriensis Boiss*. and Reut (Algeria, Morocco, and Tunisia) plant parts extract.

* PP	Product	Preparation Method	TPC	TFC	TPA	FLC	TAC	TTCs	Ref.
Algeria									
**AP**	PEE CH *n*-Bu	Mac: 1200 g in MEOH-H2O (80:20 *v*/*v*) at RT, Liquid-liquid extraction by PE, CHCl3, *n*-BuOH	*n*-Bu (318.07 ± 0.88 µg GAE/mg Ext) CH (161.78 ± 0.09 µg GAE/mg Ext) PEE (62.65 ± 0.56 µg GAE/mg Ext)	*n*-Bu (198.17 ± 0.12 µg QE/mg Ext) CH (9.77 ± 0.14 µg QE/mg Ext) PEE (8.57 ± 0.27 µg QE/mg Ext)	–	–	–	–	[58]
**n.m**	ME	3 g in MEOH for 30 mn	8.33 ± 1.15 mg GAE/g DW	2.95 ± 0.25 mg QE/g DW	–	–	–	–	[60]
**AP**	MEH PS	(1) 1st Mac: 100 g Pow in 1 L MEOH-H2O (85:15, *v*/*v*) for 24 h and 2nd Mac: MEOH-H2O (50:50, *v*/*v*) for 24 h (2) Purification: the extract was suspended in water/acetic acid (97.5:2.5, *v*/*v*) at a ratio of 1:5 (*w*/*v*) and centrifuged at 20,000× *g*, followed by solid-phase extraction	MEH (304.00 ± 3.00 µg GAE/mg Ext) PS (451.00 ± 4.00 µg GAE/mg Ext)	MEH (16.00 ± 1.00 µg QE/mg Ext) PS (40.00 ± 2.00 µg QE/mg Ext)	–	MEHext (60.00 ± 2.00 (µg RE/mg Ext) PS (107.00 ± 2.00 µg RE/mg Ext)	–	MEH (105.00 ± 2.00 µg TA/mg Ext) PS (71.00 ± 9.00 TA/mg Ext)	[188]
**L**	ET	Mac: 15 g/100 mL ETOH (100%), Incubation in a water bath at 55 °C for 6 h	125.00 ± 1.00 mg GAE/g DW	118.00 ± 1.00 mg RE/g DW	–	–	–	–	[132]
**AP**	ET ME	Mac: 50 g/500 mL. ETOH 100% + agit Mac: 50 g/500 mL. MEOH 100% + agit	67.13 mg GAE/g DW 79.45 mg GAE/g DW	25.04 mg QE/g DW 36.18 mg QE/g DW	–	–	8.14 mg C3 G/g DW 6.98 mg C3 G/g DW		[189]
**AP**	INF DEC ETH	INF: 1 g/H2O 1:100 *m*/*v* (100 °C), 5 min at RT DEC: 1 g/100 mL H2O boiling 5 min ETH: Mac 1 g/30 Ml ETOH-H2O (80:20, *v*/*v*) at 25 °C (150 rpm 1 h)	INF (256.0 ± 0.2 mg/g Ext) DEC (245 ± 4 mg/g Ext) ETH (102.30 ± 0.50 mg/g Ext)	INF (127.50 ± 0.40 mg/g Ext) DEC (119.00 ± 2.00 mg/g Ext) ETH (43.80 ± 0.80 mg/g Ext)	INF (128.50 ± 0.20 mg/g Ext) DEC (126 ± 2.00 mg/g Ext) ETH (58.50 ± 0.30 mg/g Ext)	–	–	–	[183]
**L**	MEH	Mac (1): 200 g/25 mL MEOH-H2O (80:20, *v*/*v*) Mac (2): 2.5 g/2 L of MEOH	ME1 (1.38 ± 0001 mg GAE/g DW)	ME2 (0.34 ± 0.001 mg QE/g DW)	–	–	–	–	[190]
**n.m**	n.m	1 g Pow in 7 mL of a hydro-alcoholic solution (70%), After sonication, the samples were centrifuged for 5 min, at 2000 g at 20 °C	18.73 ± 4.59 mg GAE/g DW	–	(A) 8.07 ± 2.68 mg/g DW	(B) 2.10 ± 0.54 mg QE/g DW (C) 3.24 ± 0.60 mg/g DW	–	–	[191]
**Morocco**									
**AP**	AQ	n.m	117.50 ± 6.30 mg GAE/g Pow	17.31 ±0.08 mg QE/g Pow	–	5.38 ± 0.08 mg QE/g Pow	–	–	[156]
**Tunisia**									
**AP**	MEH	Mac: 1 g/10 mL MEOH, 24 h	34.40 ± 0.80 mg GAE/g DW	10.60 ± 0.20 mg RE/g DW	–	–	–	–	[62]
**AP**	MEH	Mac 24 h	500.00 ± 11.00 µg GAE/mg Ext	180.00 ± 12.00 µg QE/mg Ext	–	–	–	94.00 ± 8.00 µg CE/mg Ext	[57]
**AP**	MEH	9 g in MEOH, 8 h (Soxhlet apparatus)	ME1 (7.08 ± 0.70 mg GAE/g DW) ME2 (8.70 ± 0.59 mg GAE/g DW) ME3 (8.81 ± 0.12 mg GAE/g DW)	ME1 (1.08 ± 0.80 mg RE/g DW) ME2 (1.95 ± 0.40 mg RE/g DW) ME3 (2.25 ± 0.43) mg RE/g DW)	–	–	–	–	[48]

* PP: part of plant; TPC: total phenolic compounds; TFC: total flavonoids compounds; TPA: total phenolic acids; FLC: flavonols contents; TAC: total anthocyanins content; TTCs: total tannins contents. (A): hydroxycinnamic acid derivatives content; AP: arial part; AQ: aqueous extract; (B): flavonols and flavones content; (C): flavanones and di-hydroflavonols content; C3G: cyanidin-3-glucoside; CE: catechin equivalents; CHCl3: chloroform; CH: chloroform extract; DEC: decoction; DW: dry weight of plant; ETH: hydroethanolic extracts; ETOH: ethanol; ET: ethanolic extract; GAE: gallic acid equivalents; INF: infusion; L: leaves; Mac: maceration; MAE: microwave-assisted extraction; MEOH: methanol; ME: methanolic extract; MEH: hydromethanolic extract; MOR: Morocco; n.m: not mentioned; *n*-BuOH: *n*-butanol; *n*-Bu: *n*-butanol extract; PE: petroleum ether; PEE: petroleum ether extract; Pow: powder; PS: purified sample; QE: quercetin equivalents; RE: rutin equivalents; TA: tannic acid equivalents; TUN: Tunisia.

## Data Availability

Not applicable.
